# Antibiotic Resistomes and Microbiomes in the Surface Water along the Code River in Indonesia Reflect Drainage Basin Anthropogenic Activities

**DOI:** 10.1021/acs.est.2c01570

**Published:** 2022-07-01

**Authors:** Johanna Muurinen, Windi I. Muziasari, Jenni Hultman, Katariina Pärnänen, Vanny Narita, Christina Lyra, Lintang N. Fadlillah, Ludhang P. Rizki, William Nurmi, James M. Tiedje, Iwan Dwiprahasto, Pramono Hadi, Marko P. J. Virta

**Affiliations:** † Department of Microbiology, University of Helsinki, Viikinkaari 9, 00014 Helsinki, Finland; ‡ Resistomap Oy, Viikinkaari 4, 00790 Helsinki, Finland; § PT. AmonRa, Jalan Panti Asuhan 37, 13330 Jakarta Timur, Indonesia; ∥ Center for Environmental Studies (PSLH), Universitas Gadjah Mada, Jalan Kuningan, 55281 Yogyakarta, Indonesia; ⊥ Faculty of Geography, Universitas Gadjah Mada, Jalan Kaliurang, 55281 Yogyakarta, Indonesia; # Faculty of Medicine, Universitas Gadjah Mada, Jalan Farmako, 55281 Yogyakarta, Indonesia; ∇ Center for Microbial Ecology, Department of Plant, Soil and Microbial Sciences, Michigan State University, East Lansing, Michigan 48824, United States

**Keywords:** Antimicrobial resistance, bacterial communities, river health, quantitative PCR, 16S rRNA amplicon sequencing

## Abstract

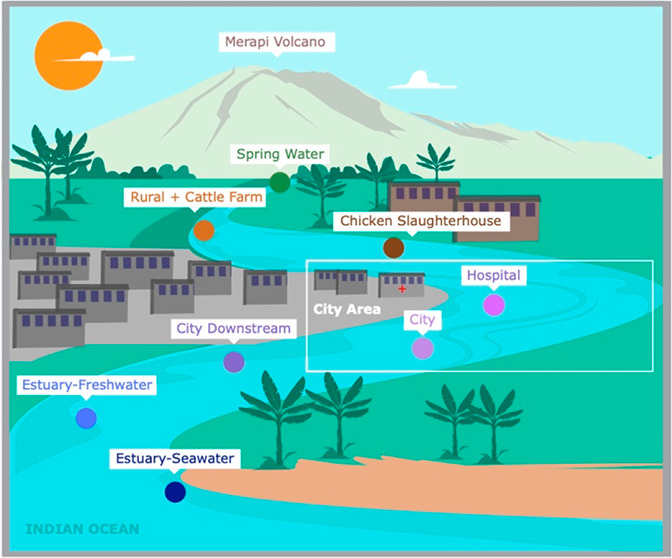

Water and sanitation are important factors in the emergence of antimicrobial resistance in low- and middle-income countries. Drug residues, metals, and various wastes foster the spread of antibiotic resistance genes (ARGs) with the help of mobile genetic elements (MGEs), and therefore, rivers receiving contaminants and effluents from multiple sources are of special interest. We followed both the microbiome and resistome of the Code River in Indonesia from its pristine origin at the Merapi volcano through rural and then city areas to the coast of the Indian Ocean. We used a SmartChip quantitative PCR with 382 primer pairs for profiling the resistome and MGEs and 16S rRNA gene amplicon sequencing to analyze the bacterial communities. The community structure explained the resistome composition in rural areas, while the city sampling sites had lower bacterial diversity and more ARGs, which correlated with MGEs, suggesting increased mobility potential in response to pressures from human activities. Importantly, the vast majority of ARGs and MGEs were no longer detectable in marine waters at the ocean entrance. Our work provides information on the impact of different influents on river health as well as sheds light on how land use contributes to the river resistome and microbiome.

## Abstract 2

Genes related to antimicrobial resistance and transfer were analyzed together with bacterial communities along the Code River in Indonesia. Genes were disseminated by fecal bacteria, and pollution increased their mobility potential.

## Introduction

The world is currently facing the threat of the “postantibiotic” era, in which antibiotics are no longer efficient for treating bacterial infections. Low- and middle-income countries carry most of the burden of infectious diseases and thus are strongly affected by the increased prevalence of antibiotic-resistant bacterial pathogens.^1^ The sources of bacterial infections can often be traced to waters,^2^ and contaminated watercourses are known disseminators of antibiotic resistance.^3−5^ Sanitation and water have been recognized as important factors in the spread and management of antimicrobial resistance,^1,3,4,6^ and pollution of rivers often acts as the driver for many life-threatening infections;^2^ however, the role of antibiotic resistance in water environments still has many open questions.^7^ We know that rivers can disseminate resistance and bacteria from different sources further downstream^8^ and that in anthropogenically impacted water environments, bacteria from different origins (agriculture, city, and industry) are mixed in the presence of multiple pollutants such as nutrients, agrochemicals, metals, antibiotic residues, and personal care products.^3,7^ These types of environments have been identified as vehicles for resistance evolution^3,9^ with possible circulation back to people.^10^ However, it is less known how much the intrinsic resistome,^11^ agricultural effluents, and pollution from humans and industry^12^ each contribute to the resistance problem disseminated through rivers.

Indonesia, a home of 261.4 million people (2017), is the fourth most populous country in the world. Despite its population size being large enough to have a considerable role in the global antibiotic resistance crisis, the dynamics between the environmental resistome and pollution caused by different anthropogenic factors has not been investigated. Overall, fragmented antibiotic resistance monitoring in low- and middle-income countries is considered as one of the key problems in tackling the spread of antibiotic resistance.^13^ It has been suggested that surveillance of waters receiving effluents from households could be utilized to gather information on the resistance burden within the population;^14^ however, this type of surveillance would require prior knowledge of the sources of resistant bacteria as well as their genes and how different human activities contribute to these.

Contaminants and the role of the environment in the evolution of antibiotic resistance are under increasing research,^7^ and the impact of human activities on ARGs found in surface waters has been previously investigated.^15−20^ While this work has been important for instance to understand the influences of wastewater treatment plant effluents on the water resistome, those studies commonly consider only one contributing factor at a time. In this study, we investigated the impact of multiple sources of antibiotic resistance contamination as well as the dynamics between the microbiome and resistome along the Code River starting from its pristine source, a spring at the Merapi volcano, to the estuary at the coast of the Indian Ocean. The investigated samples included surface water from the river in rural areas as well as the city of Yogyakarta. For profiling the river resistome and microbiome, we used a high-throughput SmartChip quantitative PCR (qPCR) array^21−23^ with 382 primers^24−26^ targeting ARGs or MGEs together with 16S rRNA gene amplicon sequencing to characterize the bacterial community compositions. Our work describes the contribution of rural and urban areas as well as the intrinsic resistome to the river resistance burden, provides information on the impact of different human activities on river health, as well as gives suggestions how the resistance load could be reduced.

## Materials and Methods

### Sampling Sites and Sample Collection

Sampling was done in the Code River in Java Island, Indonesia. The Code River is approximately 63 km long, starting from the spring at the Merapi volcano to the estuary at the Indian Ocean; 30% of the drainage basin of the river is used for agriculture, 16% of the area is forest with residents, and 52% of the area is considered urban. Besides the city of Yogyakarta with a population of 422 732 (2017), the urban area also has hospitals, laboratories, and industry. Two percent of the drainage basin is classified as other.^27^ We chose Code River for this study, since it is heavily impacted by both agriculture in the rural area as well as a large city, which makes it a suitable model for studying the impacts of different human activities on microbiomes and resistomes in river water. For instance, wastewater management in the settlement along the riverside of Code is deficient. There are only a few communal wastewater treatment plants in the Yogyakarta area, and residents of the riverside tend to flush their domestic waste directly to the river without any treatment. Due to this, many waterborne and water-deprived diseases are endemic in the area.^28^ The river belongs to the tropical rain belt and has high rainfall, with precipitation >100 mm in the wet season and <60 mm in dry season. The samples were taken in 3 days during the dry season in May 2017. All non-Indonesian researchers that participated in sampling have received the Foreign Research Permit approved by The Ministry of Research, Technology and Higher Education, Republic of Indonesia.

**Figure 1:**
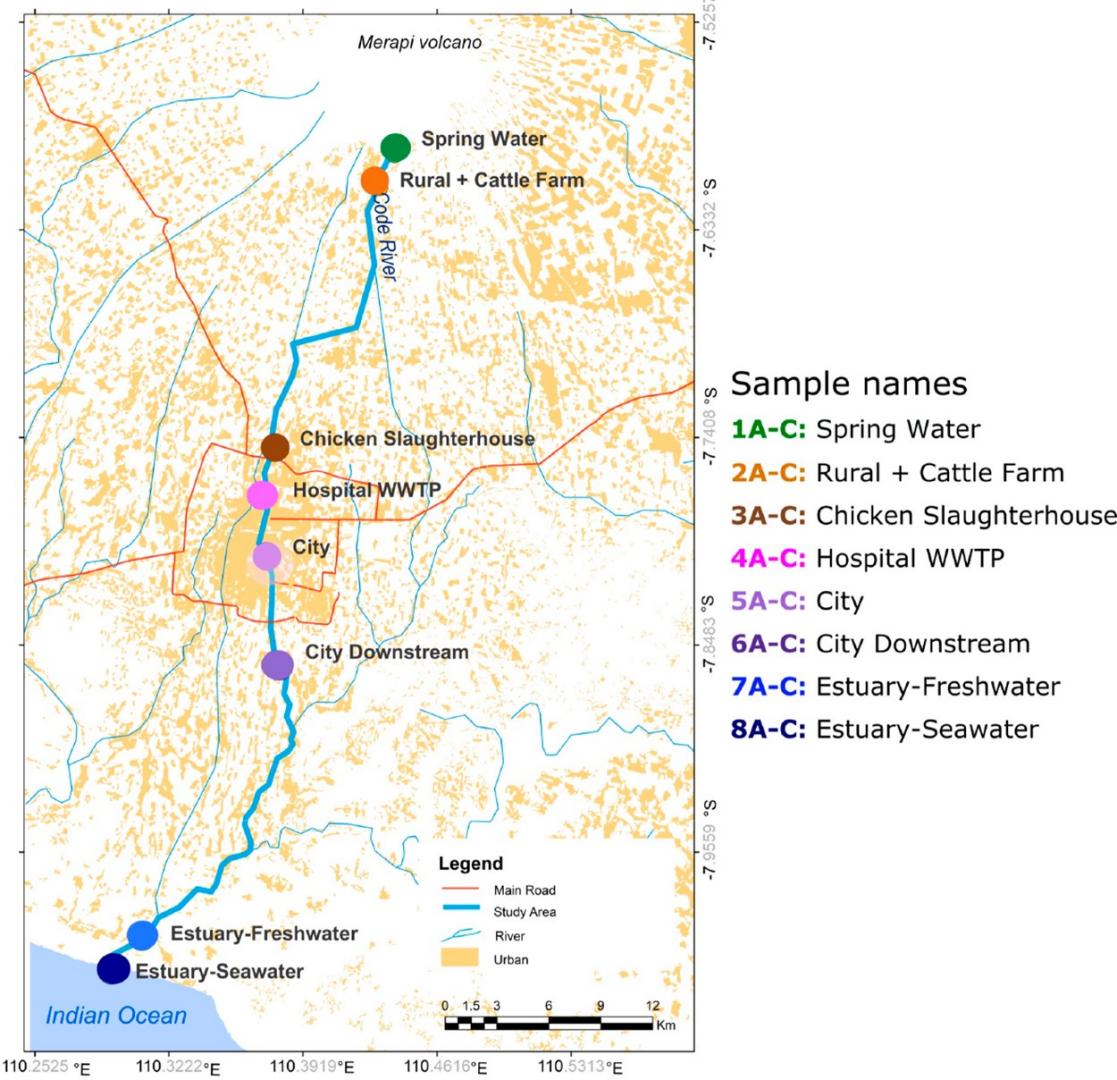
Map of the Code River displaying the sampling sites.

We took samples at eight locations along the river: (1) Spring Water, a pristine site at the spring where the river originates (7°32′39.5″S 110°26′45.8″E); (2) Rural + Cattle Farm, a site that was impacted by untreated wastewater from a local dairy farm (cattle consisting of less than 20 head) (7°35′32.6″S 110°26′31.0″E); (3) Chicken Slaughterhouse, a site that was impacted by a small chicken slaughterhouse (processing approximately 100 chickens daily) (7°44′52.3″S 110°22′32.0″E); (4) Hospital, a site that was impacted by effluent from a nearby hospital wastewater treatment plant (7°46′07.3″S 110°22′24.6″E); (5) City, a site that was impacted by untreated wastewater from riverside households (7°48′05.4″S 110°22′16.5″E); (6) City Downstream, a site after several kilometers below the city (7°50′17.0″S 110°22′37.1″E); (7) Estuary-Freshwater, a site at the estuary where the water salinity was under 0.5 ‰ (7°53′34.7″S 110°23′08.3″E); and (8) Estuary-Seawater, an estuary area with brackish water with tidal influence (8°00′50.3″S 110°17′34.2″E) (Figure 1). During sampling, floating trash was observed in the water, starting from the Hospital sampling site, after which the amount of trash increased until the coast of the Indian Ocean. At the Spring Water sites, the river flows only during a certain time each year; however, downstream of these sites to the estuary, the river flow is continuous all year. The average river flow rate across all sampling sites in May 2017 was 2.7 m^3^/s, and the range of river flow rates varied from 1 m^3^/s in November to 9 m^3^/s in February.

Three biological replicate samples were collected at each site. The volume of collected surface water was 1 L per each replicate, except for the Spring Water site where 3 L of water was collected. The samples were filtered on site using a sterile disposable filter unit with a 0.2 μm pore size PES membrane (Nalgene Rapid-Flow, Thermo Fisher Scientific). The filter membranes were transported on dry ice to the laboratory at Universitas Gadjah Mada, where they were placed in a −20 °C freezer within 3 h of sample collection and stored until DNA extraction.

### DNA Extraction and Material Transfer of Agreement (MTA)

The DNA was extracted from filter membranes using DNeasy PowerWater kits (QIAGEN), and the concentration was measured with NanoDrop One (Thermo Fisher Scientific). The transfer of the extracted DNA samples from Indonesia to Finland, which was approved by the Ministry of Health, Republic of Indonesia, was done based on the Nagoya Protocol on Access to Genetic Resources and the Fair and Equitable Sharing of Benefits Arising from their Utilization to the Convention on Biological Diversity, Material Transfer of Agreement (MTA) between Universitas Gadjah Mada in Indonesia and the University of Helsinki in Finland.

### High-Throughput Quantitative PCR Array^23^ System

DNA (1 μg) from each replicate sample was analyzed using the SmartChip Real-Time PCR (Takara Bio) with 384 primer sets (Table S1), which were validated in earlier studies^21,22,24−26,29,30^ as well as by the analysis provider before the runs with our DNA samples, and false-positive amplification was not observed. The targeted genes (Table S1) included 16S rRNA genes, four other reference genes in case 16S rRNA primers would not have amplified, antibiotic resistance genes (ARGs), mobile genetic elements (MGEs), integrons, and other genes associated with antibacterial compounds described in previous reports.^21,22,24−26^ Briefly, 5184 parallel 100 nL reactions were dispensed into the SmartChip using Multisample Nanodispenser (Takara Bio). PCR reactions, cycle conditions, and initial data processing were conducted as previously reported.^30^

### Illumina Sequencing and Analysis

PCR amplification of the V3–V4 region of the 16S rRNA gene was performed in two steps as in ref (31). Briefly, the first round of amplification with 15 cycles was done with the primers 341F1–4 and 785R1–4, which contain partial Illumina TruSeq adapter sequences at the 5′ ends using Phusion polymerase with GC buffer and 2.5% DMSO (New England Biolab, MA, USA). A second PCR round with 18 cycles was performed with full-length TruSeq P5 and Index containing P7 adapters. The final PCR products were purified with Agencourt AMPure XP magnetic beads (Beckman Coulter, CA, USA), pooled, and sequenced on the Illumina MiSeq platform at the Institute of Biotechnology, University of Helsinki, Finland.

The 16S rRNA reads were joined with Pear^32^ with default options and quality trimmed using USEARCH -fastq_filter command with -fastq_maxee 1 and -fastq_minlen 350 parameters. Unique sequences were identified with the UPARSE pipeline^33^ with -fastx_uniques command. OTUs were clustered with 97% identity, chimeras were removed, and reads were mapped to reference sequences with the --cluster_otus command with --minsize 2 parameter and -usearch_global command with -id 0.97 parameter. Taxonomic classification of OTUs was done using the classify.seqs command in mothur^34^ using the RDP naïve Bayesian Classifier^35^ against the Silva 132 database^36^ with classifier cutoff = 60.

### Data Analysis

In the SmartChip data analysis, the cycle threshold (Ct) value of 27 was used as the cutoff for detection (all samples that had higher Ct values were set to NA). The ΔCt values, ΔΔCt values, and relative gene abundances were calculated from the Ct values as previously described.^23,25^ RStudio 2021.9.0.351^37^ with R version 4.1.2 (2021-11-01)^38^ was used for data exploration,^39^ graphics, and analysis together with the ggplot2 package.^40^ Analyses of differential abundances of ARGs and MGEs were carried out using gamma distributed GLMs. *p*-values were obtained with Tukey’s posthoc test and were adjusted with false discovery rate control^41^ using *glht* function in the multcomp package.^42^ The community and resistome compositions were analyzed using the vegan package.^43^ Nonmetric multidimensional scalings (NMDSs) (Figure 4) were completed using the Bray–Curtis dissimilarity index with function *metaMDS*. Shannon diversity indexes for sampling sites were calculated by applying the function *diversity* to matrices of OTUs and ARGs and MGEs. Mantel’s test and Spearman’s rank correlation were used to analyze the interactions of bacterial community structure and resistome structure by first obtaining the Bray–Curtis dissimilarity indexes of matrices of OTUs and ARGs and MGEs with the function *vegdist*. The *mantel* function was then applied on the Bray–Curtis dissimilarity indexes. Mantel’s tests between the OTU dissimilarity matrix and dissimilarity matrix of ARGs and MGEs were also run for rural, city, and estuary sampling areas separately. Significance levels of Student’s *t* test and the Wilcoxon rank-sum test shown in Figures 5–7 were obtained with the ggpubr package.^44^ To examine if the resistance genes were associated with mobile genetic elements differently in different sampling areas, a correlation matrix between ARG and MGE relative abundances was visualized using the corrplot package^45^ (Figure S2). Spearman’s rank correlations between ARGs and MGEs within sampling areas and their *p*-values were obtained with package psych^46^ using false discovery rate control.^41^ Only ARG-MGE-pairs that were detected at least in half of the samples in a sampling area with a strong positive correlation (ρ > 0.8, adjusted *p*-value <0.05) were included.

### Data Availability

The Illumina sequencing data of the 16S rRNA genes have been deposited in the NCBI Sequence Read Archive (SRA) database under project no. PRJEB51169. The SmartChip qPCR array results are in available in the Supporting Information (Table S2) as well as at https://github.com/sjmuurine/CodeRiverIn, together with raw SmartChip qPCR array results and all data sets used in the statistical analyses, including the R code. The material transfer agreement (MTA) is available from the corresponding author upon reasonable request.

## Results and Discussion

### Microbial Community and Resistome Structures along the Code River

**Figure 2:**
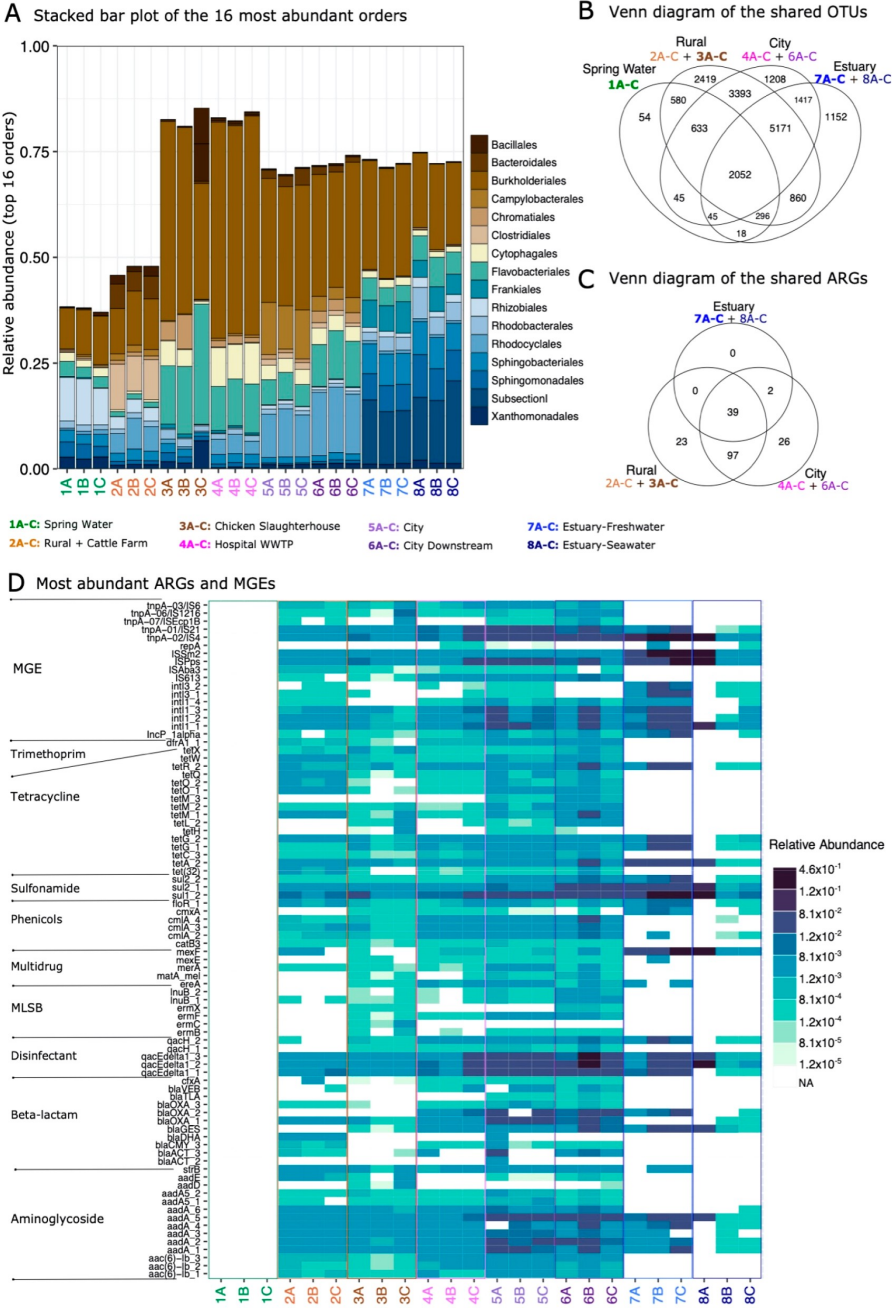
Most abundant bacterial orders and ARGs and MGEs in different sampling sites. Samples on the *x*-axis are grouped according to the sampling sites and color-coded. Sample names are shown in the legend in the middle. (A) Stacked bar plot showing 16 most abundant bacterial orders. (B) Venn diagram showing the OTUs that are shared between samples belonging to different sampling areas. (C) Venn diagram showing the ARGs and MGEs that are shared between samples belonging to different sampling areas. (D) Most abundant ARGs and MGEs (*n* = 85). Each row represents the results of each primer set (assay) (Supplementary Table S1) displayed on the *y*-axis. Assays are grouped according to the antibiotic group to which the target genes confer resistance. MLSB is the abbreviation for Macrolide, Lincosamide, Streptogramin B, and MGE for mobile genetic elements.

To analyze the contribution of different sources of effluents to the microbiome and resistome of the Code River, we collected 24 surface water samples from 8 sampling sites from the source of the river at the Merapi volcano, through the rural and city areas to the estuary at the coast of the Indian Ocean. The majority of reads clustered to OTUs were affiliated with 16 orders; however, they accounted for less than 50% of the total relative abundance of OTUs in the first two sampling sites (Figure 2A), which denotes their high bacterial diversity. *Burkholderiales* and *Rhizobiales* were the most abundant orders in the Spring Water sampling site. Members of these orders include known pathogens but also common environmental heterotrophs, bacteria capable of extracting nutrients from minerals and species able to fix atmospheric nitrogen,^47,48^ indicating oligotrophic or mesotrophic conditions at the Spring Water site. *Clostridiales*, *Bacteroidales*, *Bacillales*, *Rhodobacterales*, and *Campylobacterales* became abundant at the second sampling site (Rural + Cattle Farm) (Figure 2A), implying more available nutrients^49^ and fecal contamination,^50^ possibly due to runoff from agricultural settings. *Burkholderiales* remained the most abundant order in all sampling sites from the Spring Water site to the estuary area; however, the abundance of order *Flavobacteriales* clearly increased in the Chicken Slaughterhouse sampling site and remained elevated until the estuary (Figure 2A). Members of *Flavobacteriales* thrive in environments that are rich in carbon and other nutrients,^51^ and their increased abundance suggests that the nutrient load of the river increased in the Chicken Slaughterhouse sampling site and remained elevated until the estuary area. Cyanobacterial order SubsectionI became abundant in the estuary. SubsectionI consists of *Synechococcus*, which is dominant in marine and coastal environments.^52,53^ Interestingly, only 11% of the OTUs (2052 out of 19 433) were found in all sampling areas (Figure 2B). All four sampling areas harbored several distinct OTUs (Figure 2B), so even though a few orders were dominant in all sampling areas, the community composition varied considerably, documenting that strong source composition and selection determined the microbiome along the river.

Altogether, 187 assays targeting genes related to resistance and transfer were positive. ARGs or MGEs were not detected in the Spring Water samples. However, already at the second sampling site (Rural + Cattle Farm), 159 assays were positive (Figures 2C,D and S1). From the Rural + Cattle Farm site, the number and relative abundance of detected ARGs and MGEs mostly increased along the river until the estuary, where only a few genes remained abundant (Figure 2C,D). Interestingly, a few beta-lactam resistance genes, transposases, integrases, and some ARGs that were first found in the Rural + Cattle Farm site were either undetected or less abundant in the Chicken Slaughterhouse sampling site (Figure 2D). The river received more water from multiple side streams between these sampling sites, and thus, the bacterial load in agricultural effluents was diluted with microbiomes from different sources. Multidrug resistance genes and genes conferring resistance to antibiotics belonging to the MLSB group were mostly detected from the Chicken Slaughterhouse sampling site and thereafter. Also, the community composition changed between these sampling sites (Figure 2A). MGEs and the ARGs commonly associated with them^9,54,55^ were more abundant in the city area than in the rural area (Figure 2D). Only a few genes related to resistance and transfer remained detectable in the estuary samples, in which these genes had the highest abundances (Figure 2C,D). Since the bacterial communities of the estuary sites differed from the upstream sites (Figure 2A), it was most likely the change of conditions in the estuary that suppressed many of the bacteria carrying ARGs or MGEs, while other environmental bacteria that are not hosts for known ARGs or MGEs became more abundant, and thus, the relative abundance of genes our assays targeted decreased.

### Changes in the Abundance of ARGs and MGEs along the River

**Figure 3:**
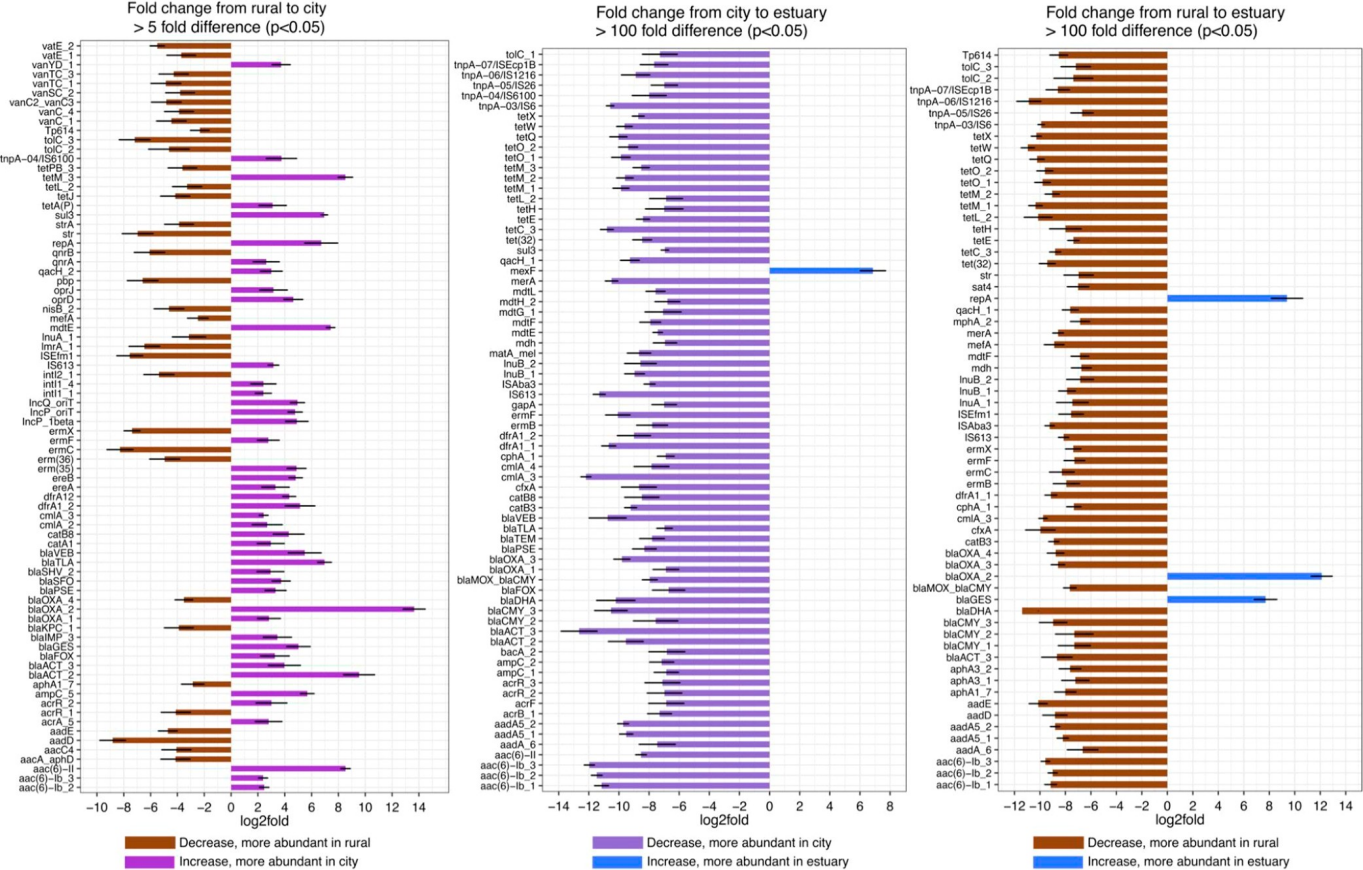
Bar plots showing the ARGs and MGEs with highest and statistically significant fold changes between sampling areas. Gamma distribution generalized linear models (GLMs) with false discovery rate control for *p*-value adjustment were used in statistical testing. (A) Comparison between rural and city areas. (B) Comparison between city and estuary areas. (C) Comparison between rural and estuary areas. See Supplementary Table S3 for fold changes of all differently abundant ARGs and MGEs.

Animal agriculture is a known disseminator of ARGs to the environment,^22,56,57^ and we know that manure was used as a fertilizer for crops in the drainage basin. A total of 38 genes related to resistance and transfer were significantly more abundant in the rural sampling sites than in the city sampling sites (adjusted *p*-value < 0.05, gamma distribution GLMs) (Figure 3, Table S3). Many of these genes have been previously found in agroecosystems under restricted antimicrobial use and in environments that were not heavily human impacted,^24,25^ like the environment of the Rural + Cattle farm site. The *ISEfm1* element together with intrinsic penicillin resistance gene *pbp* were enriched in the rural sampling sites compared to city sampling sites (Figure 3), indicating the presence of *Enterococcus*.^58,59^ Thus, there is evidence of fecal contamination,^50^ possibly originating from the production animals. The *ISEfm1* element in the rural sampling sites also significantly correlated with many ARGs that are commonly carried by fecal bacteria (adjusted *p*-value < 0.05, ρ > 0.8) (Figure S2), suggesting that runoff from fields fertilized with manure from production animals could be the source of these genes.

The abundance of most ARGs and MGEs increased significantly from rural sampling sites to the city sampling sites (66 vs 38, adjusted *p*-value <0.05, gamma distribution GLMs) (Figure 3, Table S3). It is plausible that discharges of various wastes (e.g., metals, use of personal care products, drug residues, and feces)^60^ caused the elevation of these genes in the city area.^10,61−64^ However, even though the abundance of MGEs and related ARGs increased in the city sampling sites, the increase can be caused by a shift in the taxa from rural agroecosystem-adapted bacteria to bacteria that are better adapted to humans and built environments.^64,65^ It is common that human-associated bacteria carry MGEs that are packed with multiple features, including various resistance genes.^10,63,66^ There were more ARGs co-occurring with MGEs in the city area than in the rural area sites (24 MGEs and 107 ARGs in the city area vs 19 MGEs and 70 ARGs in the rural area, adjusted *p*-value <0.05, ρ > 0.8) (Figure S2). There were also more ARGs that correlated with integrons in the city area (Figure S2); thus, the city area resistome had increased mobility potential compared to the rural area resistome. Many of the MGE-ARG pairs that co-occurred in the city area (Figure S2) have been previously found to co-occur in production animal manures in Finland and in China,^25,29^ indicating that increased fecal contamination could explain the elevated abundance of ARGs and MGEs and that ARGs persist in globally emerging antimicrobial resistance units.^67^

The vast majority of ARGs and MGEs were significantly more abundant in the city area sites and in the rural area sites than in the estuary (adjusted *p*-value <0.05, gamma distribution GLMs) (Figure 3, Table S3). This was partly due to a change in the river water conditions from fresh water to seawater and consequently a shift in the bacteria community composition. The few genes that were abundant in estuary samples can be linked to certain widely disseminated mobile elements.^54^ Therefore, although many of the genes related to resistance and transfer were abundant in the river water, especially in the city area, most of these genes did not seem to spread to the marine ecosystems.

### Bacteria Carrying ARGs and MGEs Were Sustained Especially in the City Area

**Figure 4:**
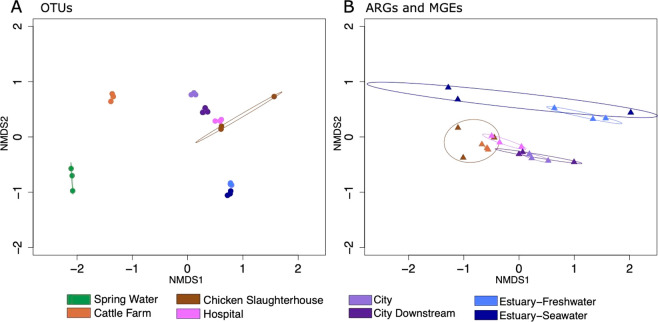
Clustering of different samples in nonmetric multidimensional scaling (NMDS) plots. (A) NMDS ordination of OTUs. (B) NMDS ordination of ARGs and MGEs. Circles show 95% confidence area for standard error of the centroids of the sampling sites. Samples in the different sampling sites are not significantly different if these confidence areas overlap.

The bacterial community compositions were distinct at different sampling sites, although the Chicken Slaughterhouse and city area sampling sites clustered close to each other (Figure 4A). On the contrary, the resistome structures were somewhat similar in rural and city sampling sites as well as within estuary sampling sites (Figure 4B). The different behaviors of the sampling sites in the ordination analysis of OTUs and the resistome together with the most abundant orders (Figure 2A) and shared OTUs, ARGs, and MGEs (Figure 2B,C) shows that the river bacterial communities were diverse and that only a small proportion of the river bacteria carried the ARGs and MGEs that our analysis targeted. The similarity of resistome structures across sampling sites is most likely explained by the fact that most of the ARGs our assays targeted are commonly embedded in MGEs that are often carried by several taxonomic groups.^10,54^

**Figure 5:**
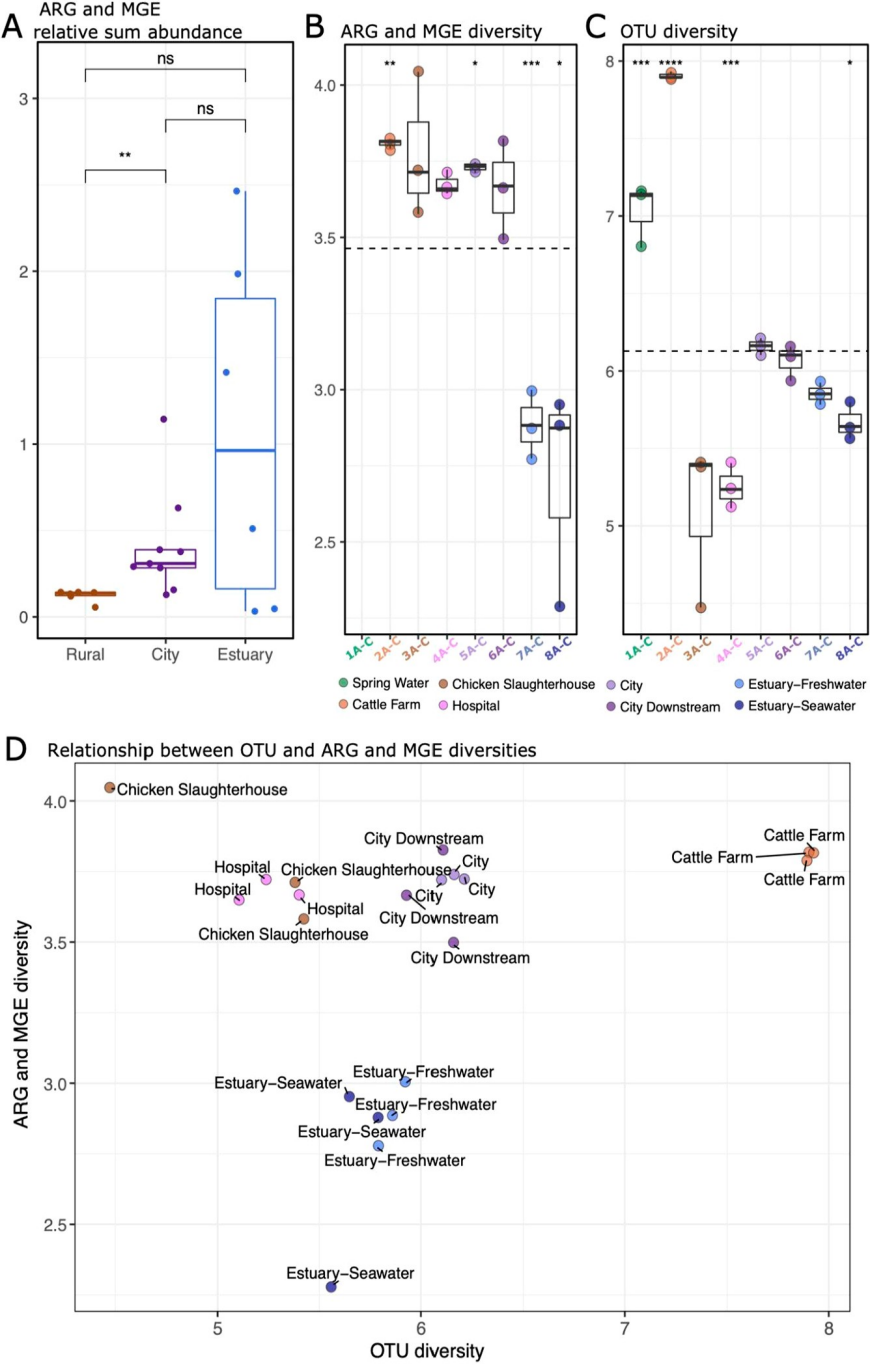
Comparisons of sum abundances and Shannon’s diversity indexes of different sampling areas. The asterisks “*”, “**”, “***”, and “****” denote statistical significance levels at *p* < 0.05, *p* < 0.01, *p* < 0.001, and *p* < 0.0001, respectively. (A) Comparison of sum abundance of ARGs and MGEs in different sampling areas using the Wilcoxon rank-sum test. (B) Comparison of Shannon’s diversity indexes of ARGs and MGEs in different sampling sites against the mean Shannon’s diversity index (dashed line) using Student’s *t* test. ARGs’ and MGEs’ r were not detected in the Spring Water sampling site. (C) Comparison of Shannon’s diversity indexes of OTUs in different sampling sites against the mean Shannon’s diversity index (dashed line) using Student’s *t* test. (D) Relationships between Shannon’s diversity indexes of OTUs and ARGs and MGEs in different sampling sites.

To get a better understanding of the dynamics between the river bacteria and genes related to resistance and transfer, the sum abundances of all ARGs and MGEs in different sampling areas and Shannon diversity indexes of all sampling sites using ARGs and MGEs as well as OTUs were compared. The sum relative abundances of ARGs and MGEs varied considerably between sampling sites and areas and increased significantly from rural area to the city area (Wilcoxon rank-sum test, *p-*value <0.01) (Figure 5A). The relative sum abundance was high particularly in the estuary samples (Figure 5A). However, the difference between estuary and other samples was not statistically significant (Wilcoxon rank-sum test, *p-*value >0.05) (Figure 5A), and only a few genes related to resistance and transfer were detected in the estuary (Figure 2D). Generally, the sampling areas that had the highest relative sum abundance of ARGs and MGEs had also the lowest diversity of OTUs (Figure 5A,C), indicating selective conditions. With both ARGs and MGEs as well as OTUs, the highest Shannon diversity index was in the Rural + Cattle farm site (Wilcoxon rank-sum test, *p-*value <0.01 and <0.0001, respectively) (Figure 5A). Interestingly, the diversity of ARGs and MGEs remained elevated until the estuary, while the diversity of OTUs dropped in the Chicken Slaughterhouse site and remained close to or lower than the mean until the estuary (Figure 5B–D). The conditions of the river since the Chicken Slaughterhouse were favorable to fewer bacterial taxa, possibly due to increased nutrient load or pressures caused by contaminants from the city, or both. Since the diversity of ARGs and MGEs remained high from the Rural + Cattle farm site through the city area and dropped only in the estuary, it seems that bacteria carrying ARGs and MGEs were the ones the city conditions favored.

Mantel’s tests with Spearman’s rank correlation were used to analyze if the bacterial community structure determines the resistome structure. The correlation coefficient between OTU and ARG and MGE distance matrices was moderate (ρ = 0.30, *p* < 0.05), which suggests that the taxonomic composition did not explain the resistome composition very well when all samples were included in the analysis. Interestingly, when rural, city, and estuary samples were analyzed individually, the correlation coefficient between OTU and ARG and MGE distance matrices was high in the rural area (ρ = 0.65, *p* < 0.05) and low in the city and estuary areas (ρ = 0.22, *p* < 0.05 and ρ = 0.18, *p* > 0.05, respectively). This supports the hypothesis that the ARGs and MGEs were brought into the river by bacteria in runoff from agricultural settings in the rural area. Also, the ARGs in the city area could be maintained at elevated levels by bacteria carrying MGEs, in which these ARGs were embedded.

### Severity of the Resistance Load and Its Relationship with Previously Analyzed Water Quality Parameters

**Figure 6:**
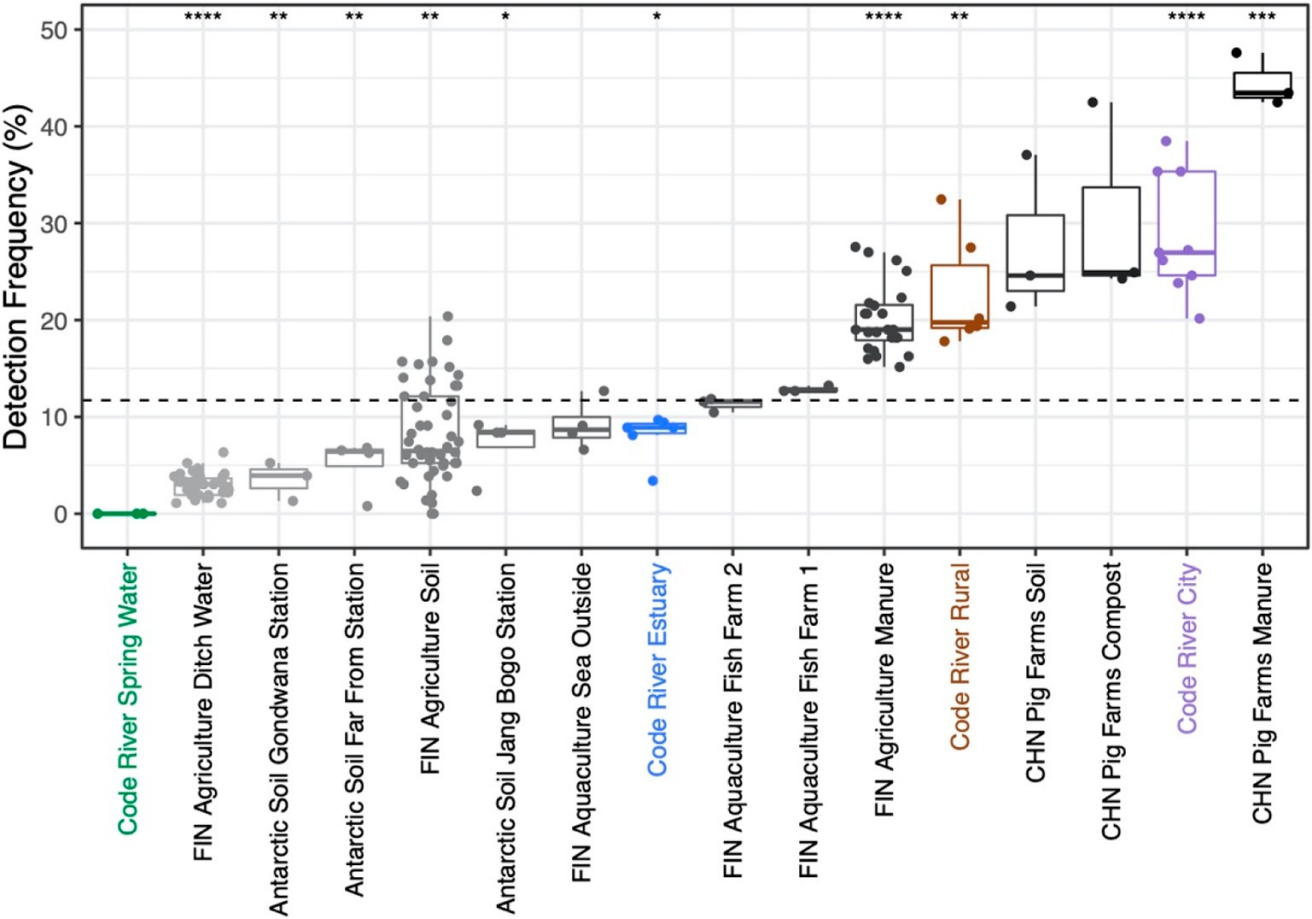
Comparison of detection frequencies (proportion of qPCR positive assays to the total number of targeted assays) of ARGs and MGEs in different sampling areas (color-coded) against published data from other studies (shades of gray) (Table S4). Samples are in increasing order, and the asterisks “*”, “**”, “***”, and “****” denote statistical significance levels at *p* < 0.05, *p* < 0.01, *p* < 0.001, and *p* < 0.0001, respectively, in the detection frequency of ARGs and MGEs against the mean detection frequency (dashed line) using Student’s *t* test. The sample names are as follows: FIN Agriculture Ditch Water = water from ditches receiving leachate and runoff from agricultural fields in Finland,^25^ Antarctic Soil Gondwana Station, Antarctic Soil Farm From Station, and Antarctic Soil Gondwana Station = soil samples from Antarctic research stations,^24^ FIN Agriculture Soil = Finnish soils before and after manure fertilization,^25^ FIN Aquaculture Sea Outside, FIN Aquaculture Fish Farm1, and FIN Aquaculture Fish Farm2 = sediments from Baltic sea outside fish farm and from under fish nets in two fish farms, respectively,^26^ FIN Agriculture Manure = Finnish production animal manure,^25^ CHN Pig Farms Soil, CHN Pig Farms Compost, and CHN Pig Farms Manure = manure fertilized soil, composted manure, and fresh manure from Chinese pig farms.^22^

To assess the severity of the river resistance load, detection frequencies of ARGs and MGEs were compared against other SmartChip qPCR studies using the same sets of primers (Table S4). Comparison revealed that rural and city area belonged to a group of sample types with detection frequencies above average, whereas the estuary area belonged to a group with detection frequencies below average (Figure 6). The group with detection frequencies above average included samples of manure and manure-fertilized soil from Chinese pig farms^22^ and manure samples from Finnish production animal farms.^25^ The detection frequency of ARGs and MGEs in the city sampling area was the second highest of all compared samples, and in the rural sampling area, the detection frequency was higher than in Finnish manure samples (Figure 6).

Water quality parameters of the Code River were analyzed in previous studies, and it was found that both rural and city areas contribute to the bacterial load of the river.^60,68^ One of the previous analyses^60^ revealed differences between the rural and city areas: A rural area roughly corresponding to Spring Water and Rural + Cattle Farm sites had lower concentrations of total dissolved solids, NO_2_^–^, NO_3_^–^, Zn, Cu, Pb, and lower biological and chemical oxygen demand than downstream sampling sites, which correspond to the city sampling area of this study. Detergent concentration and the abundance of fecal coliforms were higher in the city area (covering Hospital and City sites) than upstream (Spring Water and Rural + Cattle Farm sites) or downstream of the city (City Downstream). The increased metal and detergent concentrations^60^ could partly explain the elevation of MGEs and related ARGs we observed in the city area, since MGEs commonly carry metal and quaternary ammonium compound resistance genes.^9,10,62^ Concentrations of PO_4_^3–^ and total suspended solids were higher in the rural area (Spring Water and Rural + Cattle Farm sites) than in the city area, possibly due to soil erosion.^60^ Although making statistical connections between previously analyzed water quality parameters^60,68^ and the data of this study was not possible due to different numbers of samples and sampling times, the river quality parameters revealed that different sources contributed to the river contaminant load through different mechanisms. In the rural area, due to higher concentrations of PO_4_^3–^ and total suspended solids,^60^ it can be postulated that ARG contamination arises from soil erosion and agricultural runoffs containing feces of the production animals, whereas in the city area, the ARG contamination is most likely increased due to direct flushing of wastes to the river^28,68^ with elevated NO_2_^–^, NO_3_^–^, and detergent concentrations.^60^ Thus, decreasing the resistance load of the river could be possible with measures reducing soil erosion in the rural area and by improving wastewater treatment in the city area.

### Shifts in the Abundance of Environmental Health Indicator Bacteria along the River

**Figure 7:**
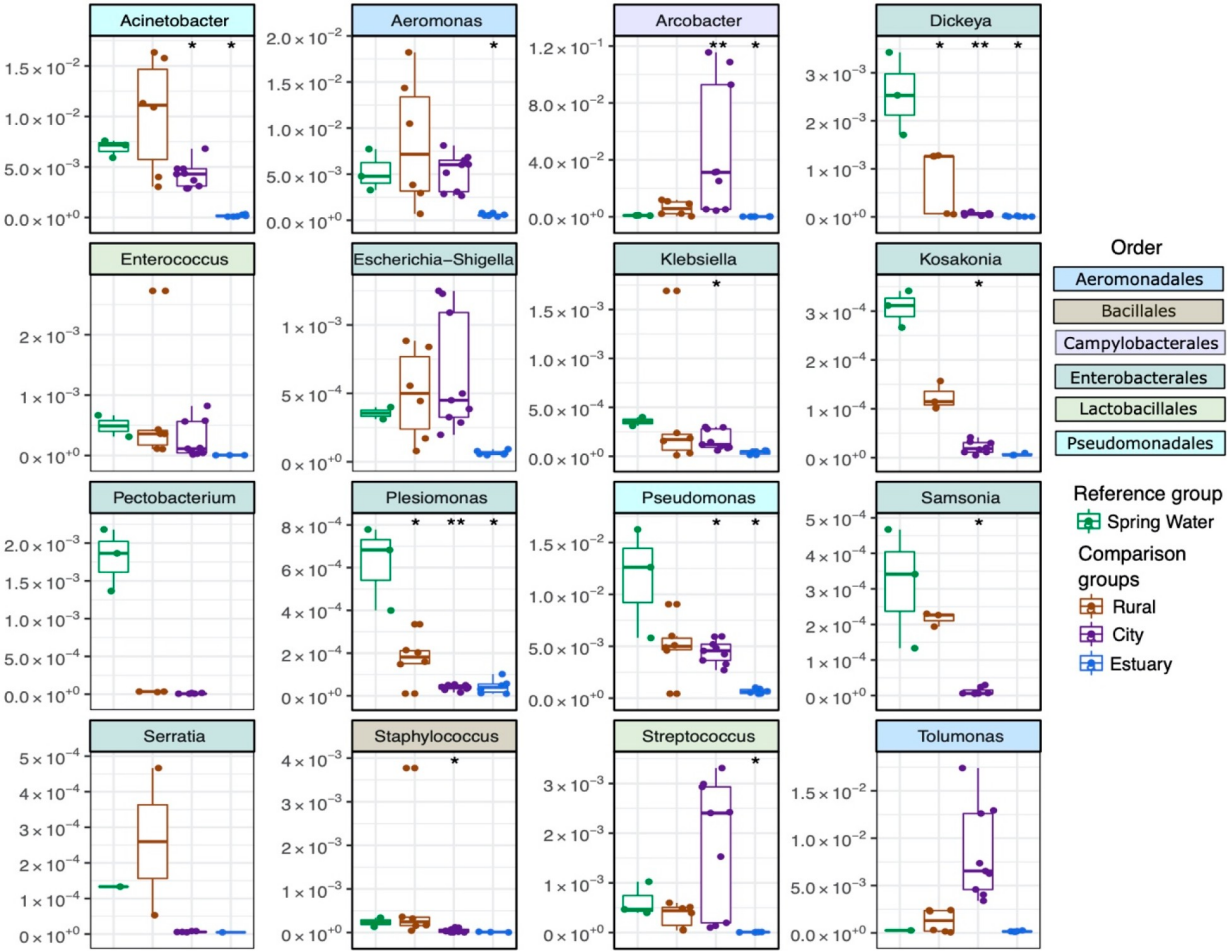
Comparison of potential pathogens (human, animal, or plant) and possible AMR traffickers in different sampling areas. The relative abundance (*y*-axis) of each genus in sampling areas (comparison groups “Rural”, “City”, and “Estuary”) was compared against the relative abundance of each genus in the Spring Water site (reference group) using the Wilcoxon rank-sum test. The asterisks “*” and “**” denote statistical significance levels at *p* < 0.05 and *p* < 0.01, respectively. Genera are color-coded according to the order displayed on the right.

Although antibiotic resistance is widely disseminated in the environment,^9,69^ most of the environmental bacteria are not associated with the genes our assays targeted, and only a small proportion of all bacterial species are responsible for the majority of clinically emerging resistant infections.^70^ To discover the sources of possible ARG traffickers as well as potential human, animal, or plant pathogens, genera belonging to families of *Enterobacteriaceae*, *Streptococcaceae*, *Staphylococcaceae*, *Campylobacteraceae*, *Moraxellaceae*, *Enterococcaceae*, *Pseudomonadaceae*, *Clostridiaceae*, and *Aeromonadaceae* were filtered from the OTU data and analyzed separately. The changes in the relative abundance of these genera in rural, city, and estuary sampling areas were compared against the pristine Spring Water sampling site. However, in most cases, the differences were not statistically significant using the Wilcoxon rank-sum test, likely due to low number of observations and high variability in abundances within the sampling areas (Figure 7).

In two sites of the rural sampling area, the abundance of *Enterococcus* was higher than in the Spring Water site, which agrees with the detection of the *ISEfm1* element and *pbp*; however, this difference was not statistically significant (Figure 7) (Wilcoxon rank-sum test, *p-*value >0.05). Other genera that were more abundant in rural area samples than in the Spring Water site were *Acinetobacter*, *Aeromonas*, *Arcobacter*, *Eschericia-Shigella*, *Klebsiella*, *Staphylococcus*, and *Tolumonas* (Figure 7). Many of these genera are known ARG traffickers and have been adapted to multiple habitats, such as feces, soil, as well as aquatic environments.^69−73^ Genera that were more abundant in the city area compared to the Spring Water site included *Arcobacter* (Wilcoxon rank-sum test, *p-*value <0.05), *Eschericia-Shigella*, *Streptococcus*, and *Tolumonas* (Figure 7). *Arcobacter* and *Tolumonas* have been previously found to possess ARGs and MGEs in wastewater and aquatic environments,^74−76^ while *Eschericia-Shigella* and *Streptococcus* are indicators for fecal contamination.^50^ From these, especially *E. coli* is known to carry a large variety of ARGs associated with MGEs that were also enriched in the city area^65,77^ (Figures 3 and S2). *Dickeya*, *Kosakonia, Pectobacterium, Plesiomonas*, and *Samsonia* were more abundant in the Spring Water site than in rural or city areas (Figure 7). Except for fish-associated *Plesiomonas*,^78^ these genera consist of plant-associated bacteria, among which *Dickeya* and *Pectobacterium* contain plant pathogens that have multiple hosts.^79,80^ It seems possible that agricultural practices aiming to suppress pathogens that can cause diseases for food crops (such as crop rotation and use of agrochemicals) could have caused the decreased abundance *Dickeya* and *Pectobacterium*. Although antimicrobials are commonly recommended for protecting food crops from these bacteria in southeast Asian countries,^81^ according to our inquiries, antimicrobials were not used for food crops in the Code River drainage basin.

The Code River resistomes and bacterial communities reflected different human activities in the drainage basin. In rural area sampling sites, the microbiome and resistome profiles supported our hypothesis that runoff including fecal contamination brought bacteria, ARGs, and MGEs into the river. Controlling soil erosion could perhaps limit the spread of agricultural ARGs and MGEs to the river. Interestingly, our results show that the potential role of MGEs in keeping ARGs elevated became more important in the city area, indicating selection toward bacteria commonly found in human impacted environments and a higher ARG mobility potential. Since wastewater treatment plants can be efficient in reducing ARG pollution,^4,16^ building up infrastructure for wastewater treatment could decrease the potential risks caused by the combination of fecal bacteria, ARGs, and MGEs observed in the city area. Despite that ARGs, MGEs, and bacteria trafficking them were abundant in the river, only a few of them remained detectable in the marine environment, most likely due to drastic changes in the water conditions at the estuary, although dilution due to tidal cycles could also contribute to their status. In summary, our work shows that rivers with drainage basins covering both rural and urban areas can be utilized in surveillance for gaining information on the sources of contaminants with potential for public health threats.

## Supporting Information Available

Venn diagram showing shared and distinct ARGs and MGEs in different sampling areas, heatmaps showing correlation between ARGs and MGEs in rural, city, and estuary sampling areas, primer sets (assays) used in this study and their target classification, relative abundances of ARGs and MGEs in all samples, results of statistical analysis of fold changes in compared samples, summary of comparison samples (PDF)
