# Supplementary material for: Antibiotic Resistomes and Microbiomes in the Surface Water along the Code River in Indonesia Reflect Drainage Basin Anthropogenic Activities
Source: Environ Sci Technol. 2022 Jul 1;56(21):14994–5006. doi: 10.1021/acs.est.2c01570 (PMC9631996; doi:10.1021/acs.est.2c01570)
Supplement: Supplementary file 1 — es2c01570_si_001.pdf [file es2c01570_si_001.pdf]

Supporting information for:

Antibiotic resistomes and microbiomes in the surface water along the Code River in Indonesia reflect drainage basin anthropogenic activities

Johanna Muurinen[1], Windi I. Muziasari[1,2], Jenni Hultman[1], Katariina Pärnänen[1], Vanny Narita[3], Christina Lyra[1], Lintang N. Fadlillah[4,5], Ludhang P. Rizki[4,6], William Nurmi[2], James M. Tiedje[7], Iwan Dwiprahasto[6]†, Pramono Hadi[4,5], Marko P. J. Virta*[1]*

[1]Department of Microbiology, Viikinkaari 9, 00014 University of Helsinki, Helsinki, Finland

[2]Resistomap Oy, Viikinkaari 4, 00790 Helsinki, Finland

[3]PT. AmonRa, Jalan Panti Asuhan 37, 13330 Jakarta Timur, Indonesia

[4]Center for Environmental Studies (PSLH), Universitas Gadjah Mada, Jalan Kuningan, 55281 Yogyakarta, Indonesia

[5]Faculty of Geography, Universitas Gadjah Mada, Jalan Kaliurang, 55281 Yogyakarta, Indonesia

[6]Faculty of Medicine, Universitas Gadjah Mada, Jalan Farmako, 55281, Yogyakarta, Indonesia

[7]Center for Microbial Ecology, Department of Plant, Soil and Microbial Sciences, Michigan State University, East Lansing, MI 48824, USA

† Died March 24, 2020

Corresponding author contact information:

Johanna Muurinen

Phone: +358 504652249

Email: johanna.muurinen@onehealth.fi

Marko P. J. Virta

Phone: +358 50448 0000

Email: marko.virta@helsinki.fi

Number of figures: 2
Number of tables: 4
Number of pages: 44

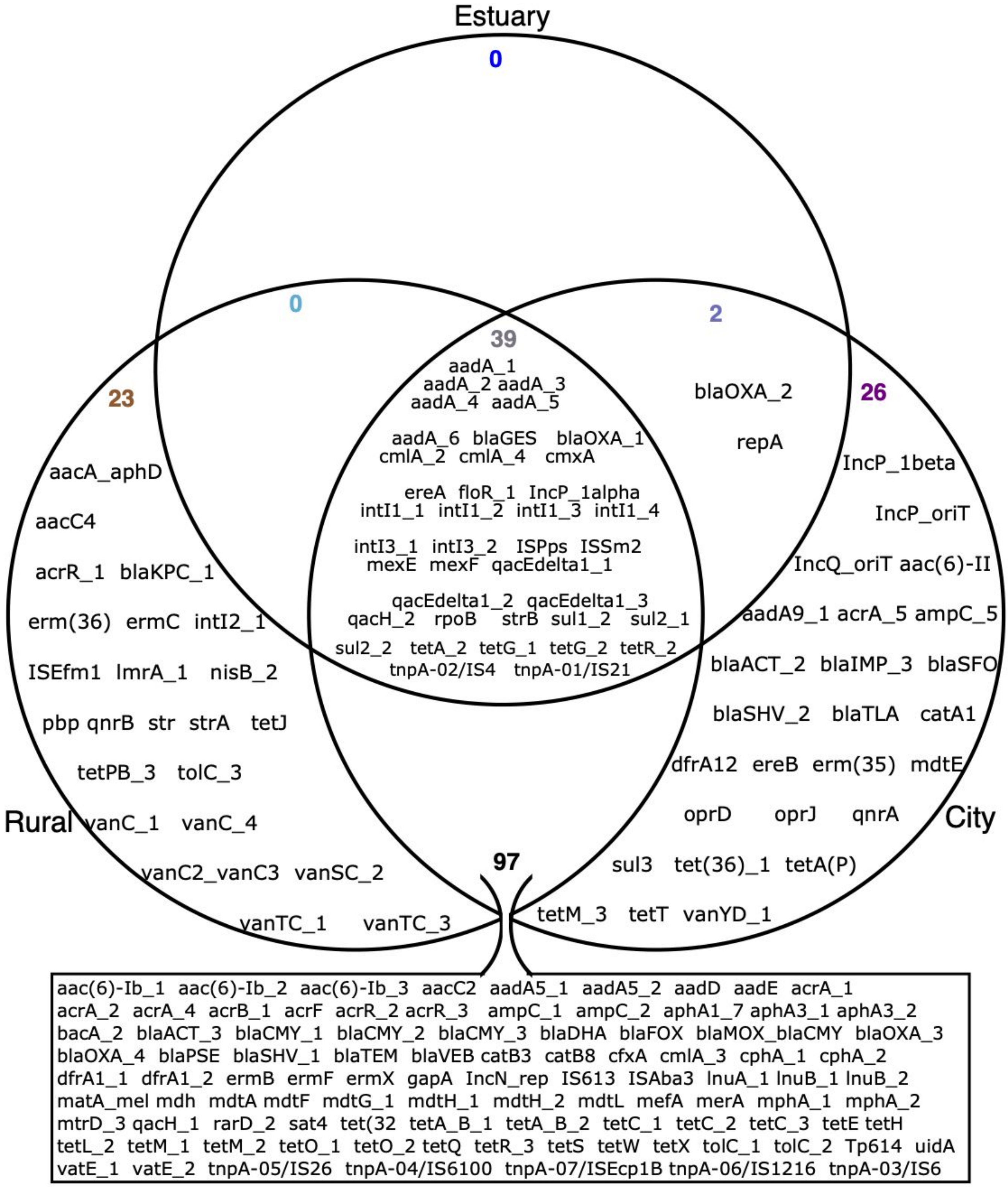

Figure S1. Venn diagram showing shared and distinct ARGs and MGEs between different sampling areas

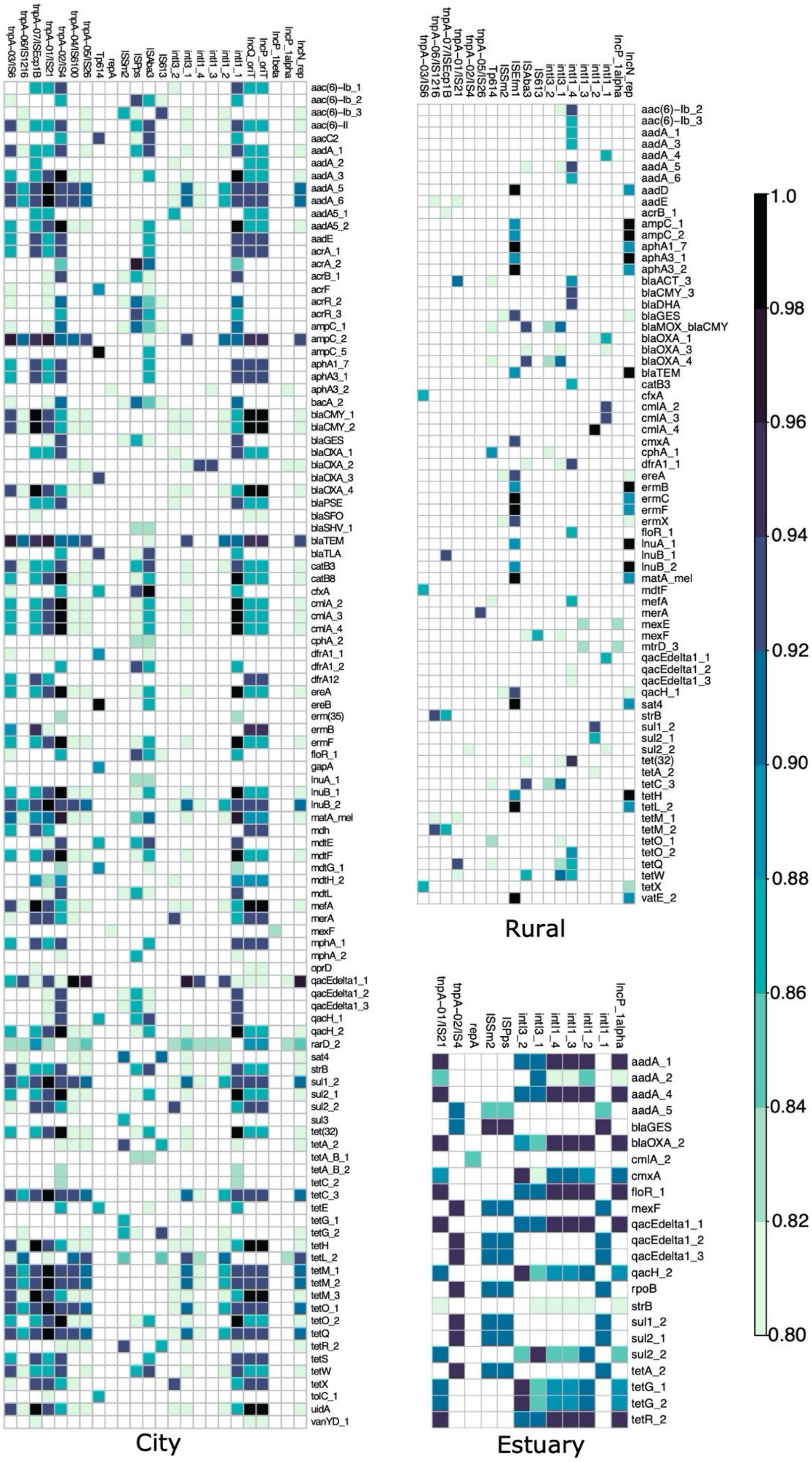

Figure S2. Heatmaps showing Spearman's correlation coefficients between ARGs and MGEs in different sampling areas. Only significant ($p<0.05$) and high (>0.8) correlation coefficients are shown

Table S1. List of the used primer sets.

Assay	Assay Name	Gene name	Forward Primer	Reverse Primer	Target antibiotic (major)	Functional classification
AY1	16S rRNA_1	16S rRNA	GGGTTGCGCTCGTTGC	ATGGYTGTCGTCAGCTCGTG	16S rRNA	16S rRNA
AY94	aac(6')-Ib_1	*aac(6')-Ib*	CGTCGCCGAGCAACTTG	CGGTACCTTGCCTCTCAAACC	Aminoglycoside	deactivate
AY95	aac(6')-Ib_2	*aac(6')-Ib*	GTTTGAGAGGCAAGGTACCGTAA	GAATGCCTGGCGTGTTTGA	Aminoglycoside	deactivate
AY172	aac(6')-Ib_3	*aac(6')-Ib*	AGAAGCACGCCCGACACTT	GCTCTCCATTCAGCATTGCA	Aminoglycoside	deactivate
AY8	aac(6')-II	*aac(6')-II*	CGACCCGACTCCGAACAA	GCACGAATCCTGCCTTCTCA	Aminoglycoside	deactivate
AY7	aac(6')-Iy	*aac(6')-Iy*	GCTTTGCGGATGCCTCAAT	GGAGAACAAAAATACCTTCAAGGAAA	Aminoglycoside	deactivate
AY5	aac(6')I1	*aac(6')I1*	GACCGGATTAAGGCCGATG	CTTGCCTTGATATTCAGTTTTTATAACCA	Aminoglycoside	deactivate
AY93	aac3-VI	*aac3-VI*	CGTCACTTATTCGATGCCCTTAC	GTCGGGCGCGGCATA	Aminoglycoside	deactivate
AY6	aacA/aphD	*aacA/aphD*	AGAGCCTTGGGAAGATGAAGTTT	TTGATCCATACCATAGACTATCTCATCA	Aminoglycoside	deactivate
AY3	aacC2	*aacC2*	ACGGCATTCTCGATTGCTTT	CCGAGCTTCACGTAAGCATTT	Aminoglycoside	deactivate
AY4	aacC4	*aacC4*	CGGCGTGGGACACGAT	AGGGAACCTTTGCCATCAACT	Aminoglycoside	deactivate
AY96	aadA_1	*aadA*	AGCTAAGCGCGAACTGCAAT	TGGCTCGAAGATACCTGCAA	Aminoglycoside	deactivate
AY97	aadA_2	*aadA*	ACGGCTCCGCAGTGGAT	GGCCACAGTAACCAACAAATCA	Aminoglycoside	deactivate
AY99	aadA_3	*aadA*	GTTGTGCACGACGACATCATT	GGCTCGAAGATACCTGCAAGAA	Aminoglycoside	deactivate
AY101	aadA_4	*aadA*	CTTGTCGTGCATGACGACATC	TCGAAGATACCCGCAAGAATG	Aminoglycoside	deactivate
AY167	aadA_5	*aadA*	CAATGACATTCTTGCGGGTATC	GACCTACCAAGGCAACGCTATG	Aminoglycoside	deactivate
AY169	aadA_6	*aadA*	CGAGATTCTCCGCGCTGTA	GCTGCCATTCTCCAAATTGC	Aminoglycoside	deactivate
AY98	aadA5_1	*aadA5*	ATCACGATCTTGCGATTTTGCT	CTGCGGATGGGCCTAGAAG	Aminoglycoside	deactivate
AY100	aadA5_2	*aadA5*	GTTCTTGCTCTTGCTCGCATT	GATGCTCGGCAGGCAAAC	Aminoglycoside	deactivate
AY168	aadA9_1	*aadA9*	CGCGGCAAGCCTATCTTG	CAAATCAGCGACCGCAGACT	Aminoglycoside	deactivate
AY173	aadA9_2	*aadA9*	GGATGCACGCTTGGATGAA	CCTCTAGCGGCCGGAGTATT	Aminoglycoside	deactivate

AY151	aadD	*aadD*	CCGACAACATTTCTACCATCCTT	ACCGAAGCGCTCGTCGTATA	Aminoglycoside	deactivate
AY174	aadE	*aadE*	TACCTTATTGCCCTTGGAAGAGTTA	GGAACTATGTCCCTTTTAATTCTAC AATCT	Aminoglycoside	deactivate
AY382	acc	acc	CCCTGCGTTGTGGCTATGT	TTGGCCACGCCAATCC	Aminoglycoside	deactivate
AY166	aph	*aph*	TTTCAGCAAGTGGATCATGTTAAAAT	CCAAGCTGTTTCCACTGTTTTTC	Aminoglycoside	deactivate
AY104	aph(2')-Ib	*aph(2')-Ib*	TGAGCAGTATCATAAGTTGAGTGAAA AG	GACAGAACAATCAATCTCTATGGA ATG	Aminoglycoside	deactivate
AY105	aph(2')-Id	*aph(2')-Id*	TAAGGATATACCGACAGTTTTGGAAA	TTTAATCCCTCTTCATACCAATCCA TA	Aminoglycoside	deactivate
AY15	aph6	*aph6*	CCCATCCCATGTGTAAGGAAA	GCCACCGCTTCTGCTGTAC	Aminoglycoside	deactivate
AY170	aphA1/7	*aphA1/7*	TGAACAAGTCTGGAAAGAAATGCA	CCTATTAATTTCCCCTCGTCAAAAA	Aminoglycoside	deactivate
AY14	aphA3_1	*aphA3*	AAAAGCCCGAAGAGGAACTTG	CATCTTTCACAAAGATGTTGCTGTC T	Aminoglycoside	deactivate
AY171	aphA3_2	*aphA3*	CGGAATTGAAAAAACTGATCGAA	ATACCGGCTGTCCGTCATTT	Aminoglycoside	deactivate
AY286	spcN_1	*spcN*	AAAAGTTCGATGAAACACGCCTAT	TCCAGTGGTAGTCCCCGAATC	Aminoglycoside	deactivate
AY287	spcN_2	*spcN*	CAGAATCTTCCTGAAAAGTTTGATGA A	CGCAGACACGCCGAATC	Aminoglycoside	deactivate
AY284	speA	*speA*	GCAAGAGGTATTTGCTCAACAAGA	CAGGGTCACCCTCATAAAGAAAA	Aminoglycoside	deactivate
AY175	str	*str*	AATGAGTTTTGGAGTGTCTCAACGTA	AATCAAAACCCCTATTAAAGCCAAT	Aminoglycoside	deactivate
AY176	strA	*strA*	CCGGTGGCATTTGAGAAAAA	GTGGCTCAACCTGCGAAAAG	Aminoglycoside	deactivate
AY177	strB	*strB*	GCTCGGTCGTGAGAACAATCT	CAATTTCGGTCGCCTGGTAGT	Aminoglycoside	deactivate
AY130	catA1	*catA1*	GGGTGAGTTTCACCAGTTTTGATT	CACCTTGTCGCCTTGCGTATA	Amphenicol	deactivate
AY51	catB3	*catB3*	GCACTCGATGCCTTCCAAAA	AGAGCCGATCCAAACGTCAT	Amphenicol	deactivate
AY52	catB8	*catB8*	CACTCGACGCCTTCCAAAG	CCGAGCCTATCCAGACATCATT	Amphenicol	deactivate
AY53	ceoA	*ceoA*	ATCAACACGGACCAGGACAAG	GGAAAGTCCGCTCACGATGA	Amphenicol	efflux
AY20	cmlA_1	*cmlA*	AATTTTGCCGATTATTGCTGAAA	GATTGTCATCATTCGTTTATCACCA A	Amphenicol	efflux

AY127	cmlA_2	*cmlA*	TAGGAAGCATCGGAACGTTGAT	CAGACCGAGCACGACTGTTG	Amphenicol	efflux
AY128	cmlA_3	*cmlA*	AGGAAGCATCGGAACGTTGA	ACAGACCGAGCACGACTGTTG	Amphenicol	efflux
AY375	cmlA_4	*cmlA*	GCGCTCTTCGAGGATTCG	CCGCCCAAGCAGAAGTAGAC	Amphenicol	efflux
AY129	cmxA	*cmxA*	GCGATCGCCATCCTCTGT	TCGACACGGAGCCTTGGT	Amphenicol	efflux
AY88	mdtL	*mdtL*	TGCTGATCGGGATTCTGATTG	CAGGCGCGACGAACATAAT	Amphenicol	efflux
AY131	rarD_1	*rarD*	GCGGGTGTGGTCACTACGAT	AGCGTTGGGCCGATATACTG	Amphenicol	efflux
AY132	rarD_2	*rarD*	TGACGCATCGCGTGATCT	AAATTTTCTGTGGCGTCTGAATC	Amphenicol	efflux
AY87	yidY/mdtL	*yidY/mdtL*	GCAGTTGCATATCGCCTTCTC	CTTCCCGGCAAACAGCAT	Amphenicol	efflux
AY56	floR_1	*floR*	ATTGTCTTCACGGTGTCCGTTA	CCGCGATGTCGTCGAACT	Amphenicol	efflux
AY335	floR_2	*floR*	TCGTCATCTACGGCCTTTTC	TCGTCATCTACGGCCTTTTC	Florfenicol	efflux
AY30	ampC_1	*ampC*	GCAGCACGCCCCGTAA	TGTACCCATGATGCGCGTACT	Beta Lactam	deactivate
AY31	ampC_2	*ampC*	AACAAAAGATCCCCGGTATGG	ACGCCCGTAAATGTTTTGCT	Beta Lactam	deactivate
AY33	ampC_3	*ampC*	TCCGGTGACGCGACAGA	CAGCACGCCGGTGAAAGT	Beta Lactam	deactivate
AY114	ampC_4	*ampC*	CCGCCCAGAGCAAGGACTA	GCTCGACTTCACGCCGTAAG	Beta Lactam	deactivate
AY258	ampC_5	*ampC*	GCAGCGAAGCGTCAGTCA	AGATCCGTGGCCGCATAA	Beta Lactam	deactivate
AY39	bla1	*bla1*	GCAAGTTGAAGCGAAAGAAAAGA	TACCAGTATCAATCGCATATACACC TAA	Beta Lactam	deactivate
AY28	blaACC	*blaACC*	CACACAGCTGATGGCTTATCTAAAA	AATAAACGCGATGGGTTCCA	Beta Lactam	deactivate
AY29	blaACT_1	*blaACT*	TGGCGTATCGGGTCAATGT	CTCCACGGGCCAGTTGAG	Beta Lactam	deactivate
AY109	blaACT_2	*blaACT*	CTGTTCGAGCTGGGTTCTATAAGTAA A	CAGTATCTGGTCACCGGATCGT	Beta Lactam	deactivate
AY110	blaACT_3	*blaACT*	CCGCTCAAGCTGGACCATAC	CCATATCCTGCACGTTGGTTT	Beta Lactam	deactivate
AY32	blaCMY_1	*blaCMY*	CCGCGGCGAAATTAAGC	GCCACTGTTTGCCTGTCAGTT	Beta Lactam	deactivate
AY108	blaCMY_2	*blaCMY*	AAAGCCTCAT GGGTGCATAAA	ATAGCTTTTGTTTGCCAGCATCA	Beta Lactam	deactivate
AY111	blaCMY_3	*blaCMY*	GCGAGCAGCCTGAAGCA	CGGATGGGCTTGTCCTCTT	Beta Lactam	deactivate
AY116	blaCTX-M_1	*blaCTX-M*	GGAGGCGTGACGGCTTTT	TTCAGTGCGATCCAGACGAA	Beta Lactam	deactivate

AY117	blaCTX-M_2	*blaCTX-M*	GCCGCGGTGCTGAAGA	ATCGGATTATAGTTAACCAGGTCA GATTT	Beta Lactam	deactivate
AY118	blaCTX-M_3	*blaCTX-M*	CGATACCACCACGCCGTTA	GCATTGCCCAACGTCAGATT	Beta Lactam	deactivate
AY119	blaCTX-M_4	*blaCTX-M*	CTTGGCGTTGCGCTGAT	CGTTCATCGGCACGGTAGA	Beta Lactam	deactivate
AY162	blaCTX-M_5	*blaCTX-M*	GCGATAACGTGGCGATGAAT	GTCGAGACGGAACGTTTCGT	Beta Lactam	deactivate
AY165	blaCTX-M_6	*blaCTX-M*	CACAGTTGGTGACGTGGCTTAA	CTCCGCTGCCGGTTTTATC	Beta Lactam	deactivate
AY324	blaCTX-M_7	*blaCTX-M*	CGTCACGCTGTTGTTAGGAA	CGCTCATCAGCACGATAAAG	Beta Lactam	deactivate
AY326	blaCTX-M_8	*blaCTX-M*	CGATGTGCAGTACCAGTAA	GCAATGGGATTGTAGTTAA	Beta Lactam	deactivate
AY112	blaDHA	*blaDHA*	TGGCCGCAGCAGAAAGA	CCGTTTTATGCACCCAGGAA	Beta Lactam	deactivate
AY113	blaFOX	*blaFOX*	GGTTTGCCGCTGCAGTTC	GCGGCCAGGTGACCAA	Beta Lactam	deactivate
AY120	blaGES	*blaGES*	GCAATGTGCTCAACGTTCAAG	GTGCCTGAGTCAATTCTTTCAAAG	Beta Lactam	deactivate
AY148	blaIMP_1	*blaIMP*	AACACGGTTTGGTGGTTCTTGTA	GCGCTCCACAAACCAATTG	Beta Lactam	deactivate
AY242	blaIMP_2	*blaIMP*	AAGGCAGCATTTCCTCTCATTTT	GGATAGATCGAGAATTAAGCCACT CT	Beta Lactam	deactivate
AY327	blaIMP_3	*blaIMP*	GGAATAGAGTGGCTTAATTC	GGTTTAACAAAACAACCACC	Beta Lactam	deactivate
AY244	blaKPC_1	*blaKPC*	TCGCCCTGGATGTACACCTT	ACCATTGCCGACATCAACAAC	Beta Lactam	deactivate
AY323	blaKPC_2	*blaKPC*	CAGCTCATTCAAGGGCTTTC	GGCGGCGTTATCACTGTATT	Beta Lactam	deactivate
AY367	blaKPC_3	*blaKPC*	GCCGCCGTGCAATACAGT	GCCGCCCAACTCCTTCA	Beta Lactam	deactivate
AY48	blaL1	*blaL1*	CACCGGGTTACCAGCTGAAG	GCGAAGCTGCGCTTGTAGTC	Beta Lactam	deactivate
AY34	blaMOX/blaC MY	*blaMOX/blaC MY*	CTATGTCAATGTGCCGAAGCA	GGCTTGTCCTCTTTCGAATAGC	Beta Lactam	deactivate
AY362	blaNDM	*blaNDM*	GGCCACACCAGTGACAATATCA	CAGGCAGCCACCAAAAGC	Beta Lactam	deactivate
AY35	blaOCH	*blaOCH*	GGCGACTTGCGCCGTAT	TTTTCTGCTCGGCCATGAG	Beta Lactam	deactivate
AY40	blaOKP	*blaOKP*	GCCGCCATCACCATGAG	GGTGACGTTGTCACCGATCTG	Beta Lactam	deactivate
AY42	blaOXY	*blaOXY*	CGTTCAGGCGGCAGGTT	GCCGCGATATAAGATTTGAGAATT	Beta Lactam	deactivate
AY44	blaOXA_1	*blaOXA*	CGCAATTATCGGCCTAGAAACT	TTGGCTTTCCGTCCCATTT	Beta Lactam	deactivate
AY45	blaOXA_2	*blaOXA*	CGCAATTATCGGCCTAGAAACT	TTGGCTTTCCGTCCCATTT	Beta Lactam	deactivate

AY115	blaOXA_3	*blaOXA*	CGGATGGTTTGAAGGGTTTATTAT	TCTTGGCTTTTATGCTTGATGTTAA	Beta Lactam	deactivate
AY322	blaOXA_4	*blaOXA*	GCAATTGCCTTTTAAACCTGA	CTGCCTTTTCAACAAAACCC	Beta Lactam	deactivate
AY36	blaPDC	*blaPDC*	CGCCGTACAACCGGTGAT	GAAGTAATGCGGTTCTCCTTTCA	Beta Lactam	deactivate
AY241	blaPER	*blaPER*	TGCTGGTTGCTGTTTTTGTGA	CCTGCGCAATGATAGCTTCAT	Beta Lactam	deactivate
AY43	blaPSE	*blaPSE*	TTGTGACCTATTCCCCTGTAATAGAA	TGCGAAGCACGCATCATC	Beta Lactam	deactivate
AY41	blaROB	*blaROB*	GCAAAGGCATGACGATTGC	CGCGCTGTTGTCGCTAAA	Beta Lactam	deactivate
AY121	blaSFO	*blaSFO*	CCGCCGCCATCCAGTA	GGGCCGCCAAGATGCT	Beta Lactam	deactivate
AY37	blaSHV_1	*blaSHV*	TCCCATGATGAGCACCTTTAAA	TTCGTCACCGGCATCCA	Beta Lactam	deactivate
AY163	blaSHV_2	*blaSHV*	CTTTCCCATGATGAGCACCTTT	TCCTGCTGGCGATAGTGGAT	Beta Lactam	deactivate
AY325	blaSHV_3	*blaSHV*	GCGTTATTTTCGCCTGTGTA	AGGTGCTCATCATGGGAAAG	Beta Lactam	deactivate
AY368	blaSME	*blaSME*	AACGGCTTCATTTTTGTTTAG	GCTTCCGCAATAGTTTTATCA	Beta Lactam	deactivate
AY259	blaSRT	*blaSRT*	CAGCCGCTGATGAAAAAATATG	CAGCGAGCCCACTTCGA	Beta Lactam	deactivate
AY164	blaTEM	*blaTEM*	AGCATCTTACGGATGGCATGA	TCCTCCGATCGTTGTCAGAAGT	Beta Lactam	deactivate
AY122	blaTLA	*blaTLA*	ACACTTTGCCATTGCTGTTTATGT	TGCAAATTTCGGCAATAATCTTT	Beta Lactam	deactivate
AY38	blaVEB	*blaVEB*	CCCGATGCAAAGCGTTATG	GAAAGATTCCCTTTATCTATCTCAG ACAA	Beta Lactam	deactivate
AY147	blaVIM	*blaVIM*	GCACTTCTCGCGGAGATTG	CGACGGTGATGCGTACGTT	Beta Lactam	deactivate
AY123	blaZ	*blaZ*	GGAGATAAAGTAACAAATCCAGTTAG ATATGA	TGCTTAATTTTCCATTTGCGATAAG	Beta Lactam	deactivate
AY24	ccrA	*ccrA*	GCAGCGTTGCTGGACACA	GTTCGGGATAAACGTGGTGACT	Beta Lactam	deactivate
AY107	cepA	*cepA*	AGTTGCGCAGAACAGTCCTCTT	TCGTATCTTGCCCGTCGATAAT	Beta Lactam	deactivate
AY106	cfxA	*cfxA*	TCATTCCTCGTTCAAGTTTTCAGA	TGCAGCACCAAGAGGAGATGT	Beta Lactam	deactivate
AY46	cphA_1	*cphA*	GCGAGCTGCACAAGCTGAT	CGGCCCAGTCGCTCTTC	Beta Lactam	deactivate
AY47	cphA_2	*cphA*	GTGCTGATGGCGAGTTTCTG	GGTGTGGTAGTTGGTGTTGATCAC	Beta Lactam	deactivate
AY65	imiR	*imiR*	CCGGACTAGAGCTTCATGTAAGC	CCCACGCGGTACTCTTGTAAA	other	regulator
AY155	mecA	*mecA*	GGTTACGGACAAGGTGAAATACTGAT	TGTCTTTTAATAAGTGAGGTGCGTT AATA	Beta Lactam	protection

AY154	pbp	*pbp*	CCGGTGCCATTGGTTTAGA	AAAATAGCCGCCCCAAGATT	Beta Lactam	protection
AY357	pbp2b	*pbp2b*	AGACGGTAACGTATAACTTTTTGAAAGA	GCGTGTAGCCGGCAATG	Beta Lactam	protection
AY237	pbp2x	*pbp2x*	TTTCATAAGTATCTGGACATGGAAGAA	CCAAAGGAAACTTGCTTGAGATTAG	Beta Lactam	protection
AY153	Pbp5	*pbp5*	GGCGAACTTCTAATTAATCCTATCCA	CGCCGATGACATTCTTCTTATCTT	Beta Lactam	protection
AY236	penA	*penA*	AGACGGTAACGTATAACTTTTTGAAAGA	GCGTGTAGCCGGCAATG	Beta Lactam	protection
AY336	intI1_1	*intI1*	CGAAGTCGAGGCATTTCTGTC	GCCTTCCAGAAAACCGAGGA	Integrase	MGE
AY337	intI1_2	*intI1*	GCCTTGATGTTACCCGAGAG	GATCGGTCGAATGCGTGT	Integrase	MGE
AY359	intI1_3	*intI1*	CGAACGAGTGGCGGAGGGTG	TACCCGAGAGCTTGGCACCCA	Integrase	MGE
AY364	intI1_4	*intI1*	CTGGATTTCGATCACGGCACG	ACATGCGTGTAAATCATCGTCG	Integrase	MGE
AY338	intI2_1	*intI2*	GACGGCTACCCTCTGTTATCTC	GCCACCACTTGTTTGAGGA	Integrase	MGE
AY360	intI2_2	*intI2*	TGCTTTTCCCACCCTTACC	GACGGCTACCCTCTGTTATCTC	Integrase	MGE
AY339	intI3_1	*intI3*	GCCACCACTTGTTTGAGGA	GGATGTCTGTGCCTGCTTG	Integrase	MGE
AY361	intI3_2	*intI3*	GCCACCACTTGTTTGAGGA	GGATGTCTGTGCCTGCTTG	Integrase	MGE
AY61	acrA_1	*acrA*	CAACGATCGGACGGGTTTC	TGGCGATGCCACCGTACT	MDR	efflux
AY62	acrA_2	*acrA*	GGTCTATCACCCTACGCGCTATC	GCGCGCACGAACATACC	MDR	efflux
AY249	acrA_3	*acrA*	CAGACCCGCATCGCATATT	CGACAATTTCGCGCTCATG	MDR	efflux
AY256	acrA_4	*acrA*	TACTTTGCGCGCCATCTTC	CGTGCGCGAACGAACAT	MDR	efflux
AY257	acrA_5	*acrA*	CGTGCGCGAACGAACA	ACTTTGCGCGCCATCTTC	MDR	efflux
AY9	acrB_1	*acrB*	AGTCGGTGTTCGCCGTTAAC	CAAGGAAACGAACGCAATACC	MDR	efflux
AY10	acrB_2	*acrB*	TGGTAGTGGGCGTCATTAACAC	GGCAACGTAATCCGAAATATCC	MDR	efflux
AY11	acrF	*acrF*	GCGGCCAGGCACAAAA	TACGCTCTTCCCACGGTTTC	MDR	efflux
AY102	acrR_1	*acrR*	GCGCTGGAGACACGACAAC	GCCTTGCTGCGAGAACAAA	MDR	regulator
AY103	acrR_2	*acrR*	GATGATACCCCCTGCTGTGAGA	ACCAAACAAGAAGCGCAAGAA	MDR	regulator
AY350	acrR_3	*acrR*	TGCAACACGCGCTTTCTC	ACGATTGCGGGCAGGTT	MDR	regulator

AY12	adeA	*adeA*	CAGTTCGAGCGCCTATTTCTG	CGCCCTGACCGACCAAT	MDR	efflux
AY27	bexA	*bexA*	GCGGATCTCTGGTCAGCAA	TGATTGATGGTTCCCCGTACA	MDR	efflux
AY277	cfr	*cfr*	GCAAAATTCAGAGCAAGTTACGAA	AAAATGACTCCCAACCTGCTTTAT	MDR	protection
AY50	cmeA	*cmeA*	GCAGCAAAGAAGAAGCACCAA	AGCAGGGTAAGTAAAACTAAGTGG TAAATCT	MDR	efflux
AY57	cmr	*cmr*	CGGCATCGTCAGTGGAATT	CGGTTCCGAAAAAGATGGAA	MDR	efflux
AY156	emrB_1	*emrB/qacA*	CTTTTCTCTAACCGTACATTATCTACG ATAAA	AGAACGTAGCGACTGATAAAATGC T	MDR	efflux
AY352	emrB_2	*emrB/qacA*	GCAGTAGAAGGAACGATTGTTAGTAC AG	TGCGTAAACCCAGCTAACAAGTT	MDR	efflux
AY64	emrD_1	*emrD*	CTCAGCAGTATGGTGGTAAGCATT	ACCAGGCGCCGAAGAAC	MDR	efflux
AY260	emrD_2	*emrD*	TTTAGGCAGCCTCGCTTCA	CCGAATCCAAATAAAACCCAATAA	MDR	efflux
AY254	marR_1	*marR*	GCGGCGTACTGGTGAAGCTA	TGCCCTGGTCGTTGATGA	MDR	regulator
AY255	marR_2	*marR*	TCTGGCGTTAGCTTCACCAGTAC	GTGCAAAGGCTGGATCGAA	MDR	regulator
AY355	marR_3	*marR*	GCTGTTGATGACATTGCTCACA	CGGCGTACTGGTGAAGCTAAC	MDR	regulator
AY80	mdetl1	*mdetl1*	ATACAGCAGTGGATATTGGTTTAATT GT	TGCATAAGGTGAATGTTCCATGA	MDR	efflux
AY253	mdsA	*mdsA*	CGGAGTCCATCGACCATTTG	ATCGTCGGCAAGGAGAATCA	MDR	efflux
AY78	mdtA	*mdtA*	CCTAACGGGCGTGACTTCA	TTCACCTGTTTCAAGGGTCAAA	MDR	efflux
AY81	mdtE	*mdtE*	CGTCGGCGCACTCGTT	TCCAGACGTTGTACGGTAACCA	MDR	efflux
AY373	mdtF	*mdtF*	CCACCATCGGGCTTTCC	CCCTTCTTTCTGCATCATCTCA	MDR	efflux
AY82	mdtG_1	*mdtG*	TGGCACAAAATATCTGGCAGTT	TTGTGTGGCGATAAGAGCATTAG	MDR	efflux
AY83	mdtG_2	*mdtG*	TTATCTGTTTTCTGCTCACCTTCTTTT	GCGTGGTGACAAACAGGCTTA	MDR	efflux
AY85	mdtH_1	*mdtH*	CGCGTGAAACCTTAAGTGCTT	AGACGGCTAAACCCCATATAGCT	MDR	efflux
AY86	mdtH_2	*mdtH*	CTGCCGTTAAATGGATGTATGC	ACTCCAGCGGGCGATAGG	MDR	efflux
AY245	mepA	*mepA*	ATCGGTCGCTCTTCGTTCAC	ATAAATAGGATCGAGCTGCTGGAT	MDR	efflux
AY89	mexA	*mexA*	AGGACAACGCTATGCAACGAA	CCGGAAAGGGCCGAAAT	MDR	efflux

AY374	mexB	*mexB*	CTGGAGATCGACGACGAGAAG	GAAATCGTTGACGTAGCTGGAA	MDR	efflux
AY90	mexD	*mexD*	TTGCCACTGGCTTTCATGAG	CACTGCGGAGAACTGTCTGTAGA	MDR	efflux
AY246	mexE	*mexE*	GGTCAGCACCGACAAGGTCTAC	AGCTCGACGTACTTGAGGAACAC	MDR	efflux
AY247	mexF	*mexF*	CCGCGAGAAGGCCAAGA	TTGAGTTCGGCGGTGATGA	MDR	efflux
AY250	mtrC	*mtrC*	GGACGGGAAGATGGTCCAA	CGTAGCGTTCCGGTTCGAT	MDR	efflux
AY230	mtrD_1	*mtrD*	TGCGCGTAGTCGTTCATCTC	CGTTCCAATTTCCTGATGATTG	MDR	efflux
AY232	mtrD_2	*mtrD*	GGTCGGCACGCTCTTGTC	TGAAGAATTTGCGCACCACTAC	MDR	efflux
AY233	mtrD_3	*mtrD*	CCGCCAAGCCGATATAGACA	GGCCGGGTTGCCAAA	MDR	efflux
AY231	mtrE	*mtrE*	CGATGTGTCGTTTTGGAAGGT	CCTGCACCATGATTCCTCAATA	MDR	efflux
AY234	oprD	*oprD*	ATGAAGTGGAGCGCCATTG	GGCCACGGCGAACTGA	MDR	efflux
AY235	oprJ	*oprJ*	ACGAGAGTGGCGTCGACAA	AAGGCGATCTCGTTGAGGAA	MDR	efflux
AY356	qac	*qac*	GGAGATTTAGCTCATGTAGCTGAAGA A	AAGCTGTTTTATCCCCGTAGCTTTA	MDR	efflux
AY262	qacA/qacB	*qacA/qacB*	TGGCAATAGGAGCTATGGTGTTT	AAGGTAACACTATTTTCGGTCCAAA TC	MDR	efflux
AY261	qacG	*qacG*	CAATAATAACCGAAATAATAGGGACA AGTT	AATAAGTGTTCCTAGTGTTGGCCAT AG	MDR	efflux
AY124	qacH_1	*qacH*	GTGGCAGCTATCGCTTGGAT	CCAACGAACGCCCACAA	MDR	efflux
AY125	qacH_2	*qacH*	CATCGTGCTTGTGGCAGCTA	TGAACGCCCAGAAGTCTAGTTTT	MDR	efflux
AY160	sdeB_1	*sdeB*	CACTACCGCTTCCGCACTTAA	TGAAAAAACGGGAAAAGTCCAT	MDR	efflux
AY353	sdeB_2	*sdeB*	GGCATGCAGAAAGTGTTTATGC	TTAAGTGCGGAAGCGGTAGTG	MDR	efflux
AY298	tolC_1	*tolC*	GGCCGAGAACCTGATGCA	AGACTTACGCAATTCCGGGTTA	MDR	efflux
AY299	tolC_2	*tolC*	CAGGCAGAGAACCTGATGCA	CGCAATTCCGGGTTGCT	MDR	efflux
AY300	tolC_3	*tolC*	GCCAGGCAGAGAACCTGATG	CGCAATTCCGGGTTGCT	MDR	efflux
AY243	ttgA	*ttgA*	ACGCCAATGCCAAACGATT	GTCACGGCGCAGCTTGA	MDR	efflux
AY84	yceL/mdtH	*yceL/mdtH*	TCGGGATGGTGGGCAAT	CGATAACCGAGCCGATGTAGA	MDR	efflux

AY341	IncN_oriT	*IncN_oriT*	TTGGGCTTCATAGTACCC	GTGTGATAGCGTGATTTATGC	plasmid incompatibility	MGE
AY340	IncN_rep	*IncN_rep*	AGTTCACCACCTACTCGCTCCG	CAAGTTCTTCTGTTGGGATTCCG	plasmid incompatibility	MGE
AY342	IncP_oriT	*IncP_oriT*	CAGCCTCGCAGAGCAGGAT	CAGCCGGGCAGGATAGGTGAAGT	plasmid incompatibility	MGE
AY343	IncQ_oriT	*IncQ_oriT*	TTCGCGCTCGTTGTTCTTCGAGC	GCCGTTAGGCCAGTTTCTCG	plasmid incompatibility	MGE
AY344	IncW_trwAB	*IncW_trwAB*	AGCGTATGAAGCCCGTGAAGGG	AAAGATAAGCGGCAGGACAATAACG	plasmid incompatibility	MGE
AY376	IS1111	*IS1111*	GTCTTAAGGTGGGCTGCGTG	CCCCGAATCTCATTGATCAGC	Insertional sequence	MGE
AY383	IS1133	*IS1133*	GCAGCGTCGGGTTGGA	ACGCGTTCGAACAACTGTAATG	Insertional sequence	MGE
AY26	IS613	*IS613*	AGGTTCGGACTCAATGCAACA	TTCAGCACATACCGCCTTGAT	Transposase	MGE
AY371	ISAba3	*ISAba3*	TCAGAGGCAGCGGTATACGA	GGTTGATTCAGTTAAAGTACGTAAAACTTT	Insertional sequence	MGE
AY372	ISEfm1	*ISEfm1*	AGGTGTCCATGACGTGAAAGTG	TCCTTTGTCCCCTAGGATATTGG	Insertional sequence	MGE
AY369	ISPps	*ISPps*	CACACTGCAAAAACGCATCCT	TGTCTTTGGCGTCACAGTTCTC	Insertional sequence	MGE
AY370	ISSm2	*ISSm2*	TGGATCGACCGGTTCCAT	GCTGACCGAGCTGTCCATGT	Insertional sequence	MGE
AY365	orf37-IS26	*orf37-IS26*	GCCGGGTTGTGCAAATAGAC	TGGCAATCTGTCGCTGCTG	Insertional sequence	MGE
AY366	orf39-IS26	*orf39-IS26*	GCGCGTCGAGCATCAATAG	CAGTTGTGCTGCTGGTGGTC	Insertional sequence	MGE

AY380	IncP_1beta	*IncP-1β*	GGTAAGATTACCGATAAACT	GTTCGTGAAGAAGATGTA	plasmid replication	MGE
AY377	pAMBL	*PAMBL*	CAGGCTCTTAATGTGATA	TTATGCTCAATACTCGTG	plasmid replication	MGE
AY379	pNI105	*pNI105*	CGCTAAGGATGTTTACAC	CTCAACCGTTCTAGGATT	plasmid replication	MGE
AY378	repA	*repA*	CCCCCAGGACTTGCGAGCG	GAGGCATGCACGCCGACCA	plasmid replication	MGE
AY384	Tn5	*Tn5*	TCAGAGGCAGCGGTATACGA	GGTTGATTCAGTTAAAGTACGTAAAACTTT	Insertional sequence	MGE
AY201	tnpA_1	*tnpA*	GCCGCACTGTCGATTTTTATC	GCGGGATCTGCCACTTCTT	Transposase	MGE
AY202	tnpA_2	*tnpA*	CCGATCACGGAAAGCTCAAG	GGCTCGCATGACTTCGAATC	Transposase	MGE
AY203	tnpA_3	*tnpA*	GGGCGGGTCGATTGAAA	GTGGGCGGGATCTGCTT	Transposase	MGE
AY204	tnpA_4	*tnpA*	CATCATCGGACGGACAGAATT	GTCGGAGATGTGGGTGTAGAAAGT	Transposase	MGE
AY205	tnpA_5	*tnpA*	GAAACCGATGCTACAATATCCAATTT	CAGCACCGTTTGCAGTGTAAG	Transposase	MGE
AY206	tnpA_6	*tnpA*	TGCAGATGGTTTAACCTTGGATATTT	TCGGTTCATCAAACTGCTTCAC	Transposase	MGE
AY207	tnpA_7	*tnpA*	AATTGATGCGGACGGCTTAA	TCACCAAACTGTTTATGGAGTCGTT	Transposase	MGE
AY25	Tp614	Tp614	GGAAATCAACGGCATCCAGTT	CATCCATGCGCTTTTGTCTCT	Transposase	MGE
AY358	trfa	*trfA*	ACGAAGAAATGGTTGTCCTGTTC	CGTCAGCTTGCGGTACTTCTC	Transposase	MGE
AY381	IncP_1alpha	*IncP-1α*	CAATCCATCGACAATCAC	GACAATCAGCTACTTCAC	plasmid replication	MGE
AY288	carB	*carB*	GGAGTGAGGCTGACCGTAGAAG	ATCGGCGAAACGCACAAA	MLSB	efflux
AY60	ereA	*ereA*	CCTGTGGTACGGAGAATTCATGT	ACCGCATTCGCTTTGCTT	MLSB	deactivate
AY63	ereB	*ereB*	GCTTTATTTCAGGAGGCGGAAT	TTTTAAATGCCACAGCACAGAATC	MLSB	deactivate
AY134	erm(34)	*erm(34)*	GCGCGTTGACGACGATTT	TGGTCATACTCGACGGCTAGAAC	MLSB	protection
AY21	erm(35)	*erm(35)*	TTGAAAACGATGTTGCATTAAGTCA	TCTATAATCACAACTAACCACTTGAACGT	MLSB	protection

AY91	erm(36)	*erm(36)*	GGCGGACCGACTTGCAT	TCTGCGTTGACGACGGTTAC	MLSB	protection
AY274	ermA	*ermA*	TTGAGAAGGGATTTGCGAAAAG	ATATCCATCTCCACCATTAATAGTAAACC	MLSB	protection
AY283	ermA/ermTR	*ermA/ermTR*	ACATTTTACCAAGGAACTTGTGGAA	GTGGCATGACATAAACCTTCATCA	MLSB	protection
AY136	ermB	*ermB*	TAAAGGGCATTTAACGACGAAACT	TTTATACCTCTGTTTGTTAGGGAATTGAA	MLSB	protection
AY275	ermC	*ermC*	TTTGAAATCGGCTCAGGAAAA	ATGGTCTATTTCAATGGCAGTTACG	MLSB	protection
AY18	ermD	*ermD*	GGACTCGGCAATGGTCAGAA	CCCCGAAACGCAATATAATGTT	MLSB	protection
AY23	ermF	*ermF*	CAGCTTTGGTTGAACATTTACGAA	AAATTCCTAAAATCACAACCGACAA	MLSB	protection
AY17	ermK_1	*ermK*	GTTTGATATTGGCATTGTCAGAGAAA	ACCATTGCCGAGTCCACTTT	MLSB	protection
AY19	ermK_2	*ermK*	GAGCCGCAAGCCCCTTT	GTGTTTCATTTGACGCGGAGTAA	MLSB	protection
AY137	ermT_1	*ermT*	GTTCACTAGCACTATTTTTAATGACAGAAGT	GAAGGGTGTCTTTTTAATACAATTAACGA	MLSB	protection
AY238	ermT_2	*ermT*	GTAAAATCCCTAGAGAATACTTTCATCCA	TGAGTGATATTTTTGAAGGGTGTCTT	MLSB	protection
AY209	ermX	*ermX*	GCTCAGTGGTCCCCATGGT	ATCCCCCCGTCAACGTTT	MLSB	protection
AY270	ermY	*ermY*	TTGTCTTTGAAAGTGAAGCAACAGT	TAACGCTAGAGAACGATTTGTATTGAG	MLSB	protection
AY77	lmrA_1	*lmrA*	TTCAGATGCAATGGCGTTTG	ATAATCGGGAACATAATGAGCATAACTAC	MLSB	efflux
AY252	lmrA_2	*lmrA*	TCGACGTGACCGTAGTGAACA	CGTGACTACCCAGGTGAGTTGA	MLSB	efflux
AY251	lnuA_1	*lnuA*	TGACGCTCAACACACTCAAAAA	TTCATGCTTAAGTTCCATACGTGAA	MLSB	deactivate
AY272	lnuA_2	*lnuA*	AGAATGAAAAAGAAGCTGAGCTTCTT	AAGGTGGCAATTACGTTTTTCAAA	MLSB	deactivate
AY67	lnuB_1	*lnuB*	TGAACATAATCCCCTCGTTTAAAGAT	TAATTGCCCTGTTTCATCGTAAATAA	MLSB	deactivate
AY68	lnuB_2	*lnuB*	AAAGGAGAAGGTGACCAATACTCTGA	GGAGCTACGTCAAACAACCAGTT	MLSB	deactivate

AY278	lnuC	*lnuC*	TGGTCAATATAACAGATGTAAACCAGATTT	CACCCCAGCCACCATCAA	MLSB	deactivate
AY139	matA/mel	*matA/mel*	TAGTAGGCAAGCTCGGTGTTGA	CCTGTGCTATTTTAAGCCTTGTTTCT	MLSB	efflux
AY146	mefA	*mefA*	CCGTAGCATTGGAACAGCTTTT	AAACGGAGTATAAGAGTGCTGCAA	MLSB	efflux
AY140	mphA_1	*mphA*	CTGACGCGCTCCGTGTT	GGTGGTGCATGGCGATCT	MLSB	deactivate
AY142	mphA_2	*mphA*	TGATGACCCTGCCATCGA	TTCGCGAGCCCCTCTTC	MLSB	deactivate
AY141	mphB	*mphB*	CGCAGCGCTTGATCTTGTAG	TTACTGCATCCATACGCTGCTT	MLSB	deactivate
AY273	mphC	*mphC*	CGTTTGAAGTACCGAATTGGAAA	GCTGCGGGTTTGCCTGTA	MLSB	deactivate
AY149	msrA_1	*msrA*	CTGCTAACACAAGTACGATTCCAAAT	TCAAGTAAAGTTGTCTTACCTACACCATT	MLSB	efflux
AY276	msrA_2	*msrA*	AACGAAATCAAGCGCAACAA	CAACCGTGCCTTTTTCTTTTG	MLSB	efflux
AY138	msrC_1	*msrC*	TCAGACCGGATCGGTTGTC	CCTATTTTTTGGAGTCTTCTCTCTAATGTT	MLSB	efflux
AY150	msrC_2	*msrC*	GAATCACTTGTCCGCAGTTTGTT	CGTACACAACGGTTTCGTCAGA	MLSB	efflux
AY285	oleC	*oleC*	CCCGGAGTCGATGTTCGA	GCCGAAGACGTACACGAACAG	MLSB	efflux
AY289	pikR1	*pikR1*	TCGACATGCGTGACGAGATT	CCGCGAATTAGGCCAGAA	MLSB	protection
AY290	pikR2	*pikR2*	TCGTGGGCCAGGTGAAGA	TTCCCCTTGCCGGTGAA	MLSB	protection
AY229	pncA	*pncA*	GCAATCGAGGCGGTGTTC	TTGCCGCAGCCAATTCA	MLSB	protection
AY224	vatB_1	*vatB*	GGAAAAAGCAACTCCATCTCTTGA	TCCTGGCATAACAGTAACATTCTGA	MLSB	deactivate
AY266	vatB_2	*vatB*	TTGGGAAAAAGCAACTCCATCT	CAATCCACACATCATTTCCAACA	MLSB	deactivate
AY225	vatC_1	*vatC*	CGGAAATTGGGAACGATGTT	GCAATAATAGCCCCGTTTCCTA	MLSB	deactivate
AY267	vatC_2	*vatC*	CGATGTTTGGATTGGACGAGAT	GCTGCAATAATAGCCCCGTTT	MLSB	deactivate
AY73	vatD	*vatD*	TGCAATAGTAGCTGCTAATTCTGTTGTT	TGTTTTATTTCGTTAGCAGGATTTCC	MLSB	deactivate
AY74	vatE_1	*vatE*	GGTGCCATTATCGGAGCAAAT	TTGGATTGCCACCGACAAT	MLSB	deactivate
AY263	vatE_2	*vatE*	GACCGTCCTACCAGGCGTAA	TTGGATTGCCACCGACAATT	MLSB	deactivate

AY226	vgaA_1	*vgaA*	CGAGTATTGTGGAAAGCAGCTAGTT	CCCGTACCGTTAGAGCCGATA	MLSB	efflux
AY265	vgaA_2	*vgaA*	GACGGGTATTGTGGAAAGCAA	TTTCCTGTACCATTAGATCCGATAATT	MLSB	efflux
AY269	vgaA_3	*vgaA*	ATACGAGCTGCCTAATAAAGGATCTT	TGTGAACCACAGGGCATTATCA	MLSB	deactivate
AY227	vgaB_1	*vgaB*	TAAAAGAGAATAAGGCGCAAGGA	TGTTTAGTAGCATGTTGCATTTTCC	MLSB	efflux
AY264	vgaB_2	*vgaB*	GAATGATTAAGCCCCCTTCAAAA	ATTCGTGTTTCCAACGATTTCG	MLSB	efflux
AY144	vgb_1	*vgb*	AGGGAGGGTATCCATGCAGAT	ACCAAATGCGCCCGTTT	MLSB	deactivate
AY145	vgb_2	*vgb*	CCACGATGGCTGCCTTTG	GGCCATGCAGGACGGATAT	MLSB	deactivate
AY268	vgbB	*vgbB*	CAGCCGGATTCTGGTCCTT	TACGATCTCCATTCAATTGGGTAAA	MLSB	deactivate
AY126	qacEdelta1_1	*qacEΔ1*	TCGCAACATCCGCATTAAAA	ATGGATTTCAGAACCAGAGAAAGAAA	MDR	efflux
AY159	qacEdelta1_2	*qacEΔ1*	CCCCTTCCGCCGTTGT	CGACCAGACTGCATAAGCAACA	MDR	efflux
AY351	qacEdelta1_3	*qacEΔ1*	GTCGGTGTTGCTTATGCAGTCT	CAACCAGGCAATGGCTGTAA	MDR	efflux
AY157	bacA_1	*bacA*	CGGCTTCGTGACCTCGTT	ACAATGCGATACCAGGCAAAT	bacitracin	deactivate
AY158	bacA_2	*bacA*	TTCCACGACACGATTAAGTCATTG	CGGCTCTTTCGGCTTCAG	bacitracin	deactivate
AY143	fosB	*fosB*	TCACTGTAACTAATGAAGCATTAGACCAT	CCATCTGGATCTGTAAAGTAAAGAGATC	other	deactivate
AY76	fosX	*fosX*	GATTAAGCCATATCACTTTAATTGTGAAAG	TCTCCTTCCATAATGCAAATCCA	other	deactivate
AY347	gapA	*gapA*	CCGTTGAAGTGAAAGACGGTC	AACCACTTTCTTCGCACCAGC	other	housekeeping
AY346	mdh	*mdh*	AAGAAACGGGCGTACTGACC	GTGGCTGATCTGACCAAACG	other	housekeeping
AY345	rpoB	*rpoB*	CGAACATCGGTCTGATCAACTC	GTTGCATGTTCGCACCCAT	other	housekeeping
AY75	uidA	*uidA*	AACCACGCGTCTGTTGACTG	CCCGGTTGCCAGAGGTG	other	housekeeping
AY331	merA	*merA*	GTGCCGTCCAAGATCATG	GGTGGAAGTCCAGTAGGGTGA	other	deactivate
AY79	nisB_1	*nisB*	GGGAGAGTTGCCGATGTTGTA	AGCCACTCGTTAAAGGGCAAT	other	deactivate
AY348	nisB_2	*nisB*	CGGTTGAACGGCGTGAA	TTTCCACCCAGGTTTGCTACTATT	other	deactivate
AY152	nimE	*nimE*	TGCGCCAAGATAGGGCATA	GTCGTGAATTCGGCAGGTTTA	other	deactivate

AY49	sat4	*sat4*	GAATGGGCAAAGCATAAAAACTTG	CCGATTTTGAAACCACAATTATGATA	other	deactivate
AY16	satA	*satA*	CCGTTGCAAGAAGATTATAGAAAAAA	CAAGCATAAGACCGCATAAATGAT	other	deactivate
AY279	fabK	*fabK*	TTTCAGCTCAGCACTTTGGTCAT	AAGGCATCTTTTTCAGCCAGTTC	other	protection
AY248	qnrA	*qnrA*	AGGATTTCTCACGCCAGGATT	CCGCTTTCAATGAAACTGCAA	Quinolone	protection
AY328	qnrB	*qnrB*	GCGACGTTCAGTGGTTCAGA	GCTGCTCGCCAGTCGAA	Quinolone	protection
AY239	pmrA	*pmrA*	TTTGCAGGTTTTGTTCCTAATGC	GCAGAGCCTGATTTCTCCTTTG	(Flouro)quinilone	efflux
AY208	folA	*folA*	CGAGCAGTTCCTGCCAAAG	CCCAGTCATCCGGTTCATAATC	Sulfonamide	protection
AY280	folP_1	*folP*	CAGGCTCGTAAATTGATAGCAGAAG	CTTTCCTTGCGAATCGCTTT	Sulfonamide	protection
AY281	folP_2	*folP*	GCGATTCGCAAGGAAAGTGA	CACATGGGCCATTTTTTCATC	Sulfonamide	protection
AY282	folP_3	*folP*	CACGGCTTCGGCTCATGT	TGCCATCCTGTGACTAGCTACGT	Sulfonamide	protection
AY178	sul1_1	*sul1*	CAGCGCTATGCGCTCAAG	ATCCCGCTGCGCTGAGT	Sulfonamide	protection
AY363	sul1_2	*sul1*	GCCGATGAGATCAGACGTATTG	CGCATAGCGCTGGGTTTC	Sulfonamide	protection
AY133	sul2_1	*sul2*	TCATCTGCCAAACTCGTCGTTA	GTCAAAGAACGCCGCAATGT	Sulfonamide	protection
AY332	sul2_2	*sul2*	TCCGGTGGAGGCCGGTATCTGG	CGGGAATGCCATCTGCCTTGAG	Sulfonamide	protection
AY333	sul3	*sul3*	TCCGTTCAGCGAATTGGTGCAG	TTCGTTCACGCCTTACACCAGC	Sulfonamide	protection
AY54	tet(32)	*tet(32)*	CCATTACTTCGGACAACGGTAGA	CAATCTCTGTGAGGGCATTTAACA	Tetracycline	protection
AY321	tet(34)	*tet(34)*	CTTAGCGCAAACAGCAATCAGT	CGGTGATACAGCGCGTAAACT	Tetracycline	deactivate
AY320	tet(35)	*tet(35)*	ACCCCATGACGTACCTGTAGAGA	CAACCCACACTGGCTACCAGTT	Tetracycline	efflux
AY22	tet(36)_1	*tet(36)*	AGAATACTCAGCAGAGGTCAGTTCCT	TGGTAGGTCGATAACCCGAAAAT	Tetracycline	protection
AY301	tet(36)_2	*tet(36)*	TGCAGGAAAGACCTCCATTACAG	CTTTGTCCACACTTCCACGTACTATG	Tetracycline	protection
AY302	tet(37)	*tet(37)*	GAGAACGTTGAAAAGGTGGTGAA	AACCAAGCCTGGATCAGTCTCA	Tetracycline	deactivate
AY271	tet(38)	*tet(38)*	TTAATGTGGCGGTATCTGTAGGTATT	TTGCCTGGGAAATTTAATGCTTT	Tetracycline	efflux
AY13	tetA_1	*tetA*	GCTGTTTGTTCTGCCGGAAA	GGTTAAGTTCCTTGAACGCAAACT	Tetracycline	efflux
AY180	tetA_2	*tetA*	CTCACCAGCCTGACCTCGAT	CACGTTGTTATAGAAGCCGCATAG	Tetracycline	efflux

AY55	tetA(P)	*tetA(P)*	AGTTGCAGATGTGTATAGTCGTAAACTATCTATT	TGCTACAAGTACGAAAACAAAACTAGAA	Tetracycline	efflux
AY181	tetA/B_1	*tetA/B*	AGTGCGCTTTGGATGCTGTA	AGCCCCAGTAGCTCCTGTGA	Tetracycline	efflux
AY190	tetA/B_2	*tetA/B*	GCCCAGTGCTGTTGTTGTCAT	TGAAAGCAAACGGCCTAAATACA	Tetracycline	efflux
AY182	tetC_1	*tetC*	CATATCGCAATACATGCGAAAAA	AAAGCCGCGGTAAATAGCAA	Tetracycline	efflux
AY199	tetC_2	*tetC*	ACTGGTAAGGTAAACGCCATTGTC	ATGCATAAACCAGCCATTGAGTAAG	Tetracycline	efflux
AY330	tetC_3	*tetC*	TGCGTTGATGCAATTTCTATGC	GGAATGGTGCATGCAAGGAG	Tetracycline	efflux
AY92	tetD_1	*tetD*	TGCCGCGTTTGATTACACA	CACCAGTGATCCCGGAGATAA	Tetracycline	efflux
AY188	tetD_2	*tetD*	TGTCATCGCGCTGGTGATT	CATCCGCTTCCGGGAGAT	Tetracycline	efflux
AY349	tetD_3	*tetD*	CTGGACGCGATGGGAATT	TCCGCTTCCGGGAGATATT	Tetracycline	efflux
AY291	tetE	*tetE*	TTGGCGCTGTATGCAATGAT	CGACGACCTATGCGATCTGA	Tetracycline	efflux
AY183	tetG_1	*tetG*	TCAACCATTGCCGATTCGA	TGGCCCGGCAATCATG	Tetracycline	efflux
AY189	tetG_2	*tetG*	CATCAGCGCCGGTCTTATG	CCCCATGTAGCCGAACCA	Tetracycline	efflux
AY187	tetH	*tetH*	TTTGGGTCATCTTACCAGCATTAA	TTGCGCATTATCATCGACAGA	Tetracycline	efflux
AY240	tetJ	*tetJ*	GGGTGCCGCATTAGATTACCT	TCGTCCAATGTAGAGCATCCATA	Tetracycline	efflux
AY184	tetK	*tetK*	CAGCAGTCATTGGAAAATTATCTGATTATA	CCTTGTACTAACCTACCAAAAATCAAAATA	Tetracycline	efflux
AY194	tetL_1	*tetL*	AGCCCGATTTATTCAAGGAATTG	CAAATGCTTTCCCCCTGTTCT	Tetracycline	efflux
AY195	tetL_2	*tetL*	ATGGTTGTAGTTGCGCGCTATAT	ATCGCTGGACCGACTCCTT	Tetracycline	efflux
AY186	tetM_1	*tetM*	CATCATAGACACGCCAGGACATAT	CGCCATCTTTTGCAGAAATCA	Tetracycline	protection
AY193	tetM_2	*tetM*	TAATATTGGAGTTTTAGCTCATGTTGATG	CCTCTCTGACGTTCTAAAAGCGTATTAT	Tetracycline	protection
AY329	tetM_3	*tetM*	GCAATTCTACTGATTTCTGC	CTGTTTGATTACAATTTCCGC	Tetracycline	protection
AY179	tetO_1	*tetO*	ATGTGGATACTACAACGCATGAGATT	TGCCTCCACATGATATTTTTCCT	Tetracycline	protection
AY192	tetO_2	*tetO*	CAACATTAACGGAAAGTTTATTGTATACCA	TTGACGCTCCAAATTCATTGTATC	Tetracycline	protection

AY292	tetPB_1	*tetPB*	ACACCTGGACACGCTGATTTT	ACCGTCTAGAACGCGGAATG	Tetracycline	protection
AY293	tetPB_2	*tetPB*	TGATACACCTGGACACGCTGAT	CGTCCAAAACGCGGAATG	Tetracycline	protection
AY294	tetPB_3	*tetPB*	TGGGCGACAGTAGGCTTAGAA	TGACCCTACTGAAACATTAGAAATATACCT	Tetracycline	protection
AY295	tetPB_4	*tetPB*	AGTGGTGCAAATACTGAAAAAGTTGT	TTTGTTCCTTCGTTTTGGACAGA	Tetracycline	protection
AY296	tetPB_5	*tetPB*	CTGAAGTGGAGCGATCATTCC	CCCTCAACGGCAGAAATAACTAA	Tetracycline	protection
AY185	tetQ	*tetQ*	CGCCTCAGAAGTAAGTTCATACACTAAG	TCGTTCATGCGGATATTATCAGAAT	Tetracycline	protection
AY135	tetR_1	*tetR*	ATGAGTTCGGCCAGAATTTCC	GGTTGTGCGCGAAATGATT	Tetracycline	regulator
AY197	tetR_2	*tetR*	CGCGATAGACGCCTTCGA	TCCTGACAACGAGCCTCCTT	Tetracycline	regulator
AY198	tetR_3	*tetR*	CGCGATGGAGCAAAAGTACAT	AGTGAAAAACCTTGTTGGCATAAAA	Tetracycline	regulator
AY200	tetS	*tetS*	TTAAGGACAAACTTTCTGACGACATC	TGTCTCCCATTGTTCTGGTTCA	Tetracycline	protection
AY297	tetT	*tetT*	CCATATAGAGGTTCCACCAAATCC	TGACCCTATTGGTAGTGGTTCTATTG	Tetracycline	protection
AY69	tetU_1	*tetU*	GTGGCAAAGCAACGGATTG	TGCGGGCTTGCAAAACTATC	Tetracycline	unknown
AY70	tetU_2	*tetU*	AACAGCGGGTTAAGTGTGCAA	ATGGTATCATTCAGTTTTCCGACAAT	Tetracycline	unknown
AY228	tetV	*tetV*	GCGGGAACGACGATGTATATC	CCGCTATCTCACGACCATGAT	Tetracycline	efflux
AY191	tetW	*tetW*	ATGAACATTCCCACCGTTATCTTT	ATATCGGCGGAGAGCTTATCC	Tetracycline	protection
AY196	tetX	*tetX*	AAATTTGTTACCGACACGGAAGTT	CATAGCTGAAAAAATCCAGGACAGTT	Tetracycline	deactivate
AY58	dfrA1_1	*dfrA1*	GGAATGGCCCTGATATTCCA	AGTCTTGCGTCCAACCAACAG	Trimethoprim	deactivate
AY334	dfrA1_2	*dfrA1*	TTCAGGTGGTGGGGAGATATAC	TTAGAGGCGAAGTCTTGGGTAA	Trimethoprim	deactivate
AY59	dfrA12	*dfrA12*	CCTCTACCGAACCGTCACACA	GCGACAGCGTTGAAACAACTAC	Trimethoprim	deactivate
AY210	vanA	*vanA*	AAAAGGCTCTGAAAACGCAGTTAT	CGGCCGTTATCTTGTAAAAACAT	Vancomycin	protection
AY211	vanB_1	*vanB*	TTGTCGGCGAAGTGGATCA	AGCCTTTTTCCGGCTCGTT	Vancomycin	protection
AY212	vanB_2	*vanB*	CCGGTCGAGGAACGAAATC	TCCTCCTGCAAAAAAAGATCAAC	Vancomycin	protection

AY354	vanB_3	*vanB*	GGCTGTTTCGGGCTGTGA	AACAACTAACGCGGCACTGTT	Vancomycin	protection
AY66	vanC_1	*vanC*	ACAGGGATTGGCTATGAACCAT	TGACTGGCGATGATTTGACTATG	Vancomycin	protection
AY71	vanC_2	*vanC*	CCTGCCACAATCGATCGTT	CGGCTTCATTCGGCTTGATA	Vancomycin	protection
AY72	vanC_3	*vanC*	AGGCGATAGCGGGTATTGAA	CAATCGTCAATTGCTCATTTCC	Vancomycin	protection
AY303	vanC_4	*vanC*	AAATCAATACTATGCCGGGCTTT	CCGACCGCTGCCATCA	Vancomycin	protection
AY304	vanC2/vanC3	*vanC2/vanC3*	TTTGACTGTCGGTGCTTGTGA	TCAATCGTTTCAGGCAATGG	Vancomycin	protection
AY213	vanD	*vanD*	CAGAGGAACATAATGTTTCGATAAAATCT	GCCGGATTTTGTGATTCCAA	Vancomycin	protection
AY305	vanG	*vanG*	ATTTGAATTGGCAGGTATACAGGTTA	TGATTTGTCTTTGTCCATACATAATGC	Vancomycin	protection
AY215	vanHB	*vanHB*	GAGGTTTCCGAGGCGACAA	CTCTCGGCGGCAGTCGTAT	Vancomycin	protection
AY214	vanHD	*vanHD*	GTGGCCGATTATACCGTCATG	CGCAGGTCATTCAGGCAAT	Vancomycin	protection
AY216	vanRA_1	*vanRA*	CCCTTACTCCCACCGAGTTTT	TTCGTCGCCCCATATCTCAT	Vancomycin	protection
AY217	vanRA_2	*vanRA*	CCACTCCGGCCTTGTCATT	GCTAACCACATTCCCCTTGTTTT	Vancomycin	protection
AY306	vanRB	*vanRB*	GCCCTGTCGGATGACGAA	TTACATAGTCGTCTGCCTCTGCAT	Vancomycin	protection
AY307	vanRC	*vanRC*	TGCGGGAAAAACTGAACGA	CCCCCCATACGGTTTTGATTA	Vancomycin	protection
AY308	vanRC4	*vanRC4*	AGTGCTTTGGCTTATCTCGAAAA	TCCGGCAGCATCACATCTAA	Vancomycin	protection
AY309	vanRD	*vanRD*	TTATAATGGCAAGGATGCACTAAAGT	CGTCTACATCCGGAAGCATGA	Vancomycin	protection
AY218	vanSA	*vanSA*	CGCGTCATGCTTTCAAAATTC	TCCGCAGAAAGCTCAATTTGTT	Vancomycin	protection
AY310	vanSB	*vanSB*	GCGCGGCAAATGACAAC	TTTGCCATTTTATTCGCACTGT	Vancomycin	protection
AY311	vanSC_1	*vanSC*	ATCAACTGCGGGAGAAAAGTCT	TCCGCTGTTCCGCTTCTT	Vancomycin	protection
AY312	vanSC_2	*vanSC*	GCCATCAGCGAGTCTGATGA	CAGCTGGGATCGTTTTTCCTT	Vancomycin	protection
AY313	vanSE	*vanSE*	TGGCCGAAGAAGCAGGAA	CAATAATACTCGTCAAAGGAGTTCTCA	Vancomycin	protection
AY161	vanTC_1	*vanTC*	CACACGCATTTTTTCCCATCTAG	CAGCCAACAGATCATCAAAACAA	Vancomycin	protection
AY314	vanTC_2	*vanTC*	GTGGTGCCAAGGAAGTTGCT	CGTAGCCACCGCAAAAAAAT	Vancomycin	protection
AY315	vanTC_3	*vanTC*	ACAGTTGCCGCTGGTGAAG	CGTGGCTGGTCGATCAAAA	Vancomycin	protection

AY316	vanTG	*vanTG*	CGTGTAGCCGTTCCGTTCTT	CGGCATTACAGGTATATCTGGAAA	Vancomycin	protection
AY220	vanWB	*vanWB*	CGGACAAAGATACCCCCTATAAAG	AAATAGTAAATTGCTCATCTGGCACAT	Vancomycin	protection
AY219	vanWG	*vanWG*	ACATTTTCATTTTGGCAGCTTGTAC	CCGCCATAAGAGCCTACAATCT	Vancomycin	protection
AY221	vanXA	*vanXA*	CGCTAAATATGCCACTTGGGATA	TCAAAAGCGATTCAGCCAACT	Vancomycin	protection
AY223	vanXB	*vanXB*	AGGCACAAAATCGAAGATGCTT	GGGTATGGCTCATCAATCAACTT	Vancomycin	protection
AY222	vanXD	*vanXD*	TAAACCGTGTTATGGGAACGAA	GCGATAGCCGTCCCATAAGA	Vancomycin	protection
AY317	vanYB	*vanYB*	GGCTAAAGCGGAAGCAGAAA	GATATCCACAGCAAGACCAAGCT	Vancomycin	protection
AY318	vanYD_1	*vanYD*	AAGGCGATACCCTGACTGTCA	ATTGCCGGACGGAAGCA	Vancomycin	protection
AY319	vanYD_2	*vanYD*	CAAACGGAAGAGAGGTCACTTACA	CGGACGGTAATAGGGACTGTTC	Vancomycin	protection

Table S2. The SmartChip qPCR results (relative abundances)

Assay name	Spring water	Spring water	Spring water	Rural + Cattle Farm	Rural + Cattle Farm	Rural + Cattle Farm	Chicken Slaughterhouse	Chicken Slaughterhouse	Chicken Slaughterhouse	Hospital	Hospital	Hospital	City	City	City	City Downstream	City Downstream	City Downstream	Estuary_Freshwater	Estuary_Freshwater	Estuary_Freshwater	Estuary_Seawater	Estuary_Seawater	Estuary_Seawater
aac(6')-Ib_1				8.56E-04	4.71E-04	9.51E-04	5.40E-04	1.15E-03	3.21E-04	1.74E-03	2.65E-04	4.85E-04	4.67E-03	3.57E-03	2.65E-03	1.03E-03	2.40E-03	7.05E-04	6.62E-04	1.62E-03	2.57E-03		4.54E-05	
aac(6')-Ib_2				9.09E-04	6.14E-04	6.84E-04	3.83E-04	1.23E-04	2.31E-04	1.15E-03	1.20E-03	2.89E-03	4.62E-03	3.47E-03	4.00E-03	9.60E-04	3.40E-03	7.35E-04						
aac(6')-Ib_3				1.14E-03	1.14E-03	1.16E-03	7.57E-04	1.35E-04	2.79E-04	1.95E-03	2.51E-03	5.23E-03	6.42E-03	4.16E-03	4.30E-03	1.96E-03	6.72E-03	1.89E-03	6.29E-04		2.14E-03			9.96E-05
aac(6')-II				8.85E-05		1.44E-04	2.55E-05	2.47E-05		1.02E-04	1.34E-04	2.10E-04	8.12E-04	7.00E-04	4.93E-04	1.12E-04		1.38E-04						
aacA/aphD									9.65E-05				1.97E-05	1.77E-05										
aacC2							2.79E-05		1.64E-04				7.03E-05	1.28E-04	9.98E-05									
aacC4									9.36E-05															
aadA_1				4.84E-03	4.75E-03	6.54E-03	4.50E-03	1.23E-03	2.10E-03	2.62E-03	2.77E-03	5.79E-03	1.79E-02	1.44E-02	1.08E-02	6.19E-03	1.88E-02	8.91E-03	2.76E-03	9.04E-03	1.41E-02		2.00E-04	4.34E-04
aadA_2				2.29E-03	2.81E-03	2.34E-03	2.39E-03	8.31E-04	1.39E-03	3.24E-03	2.86E-03	2.38E-03	1.31E-02	1.20E-02		1.31E-02	1.46E-02	8.52E-03	5.60E-03	3.18E-03	1.25E-02	5.80E-02	2.94E-04	2.74E-04
aadA_3				3.83E-03	3.06E-03	4.17E-03	2.61E-03	8.73E-04	1.35E-03	2.54E-03	1.81E-03	2.81E-03	1.14E-02	9.61E-03	1.50E-02	6.66E-03	9.22E-03	5.04E-03	1.58E-03	2.52E-03			9.04E-05	7.43E-05

aadA_4				2.75E-03	2.54E-03	2.81E-03	2.78E-03	9.57E-04	1.55E-03	2.46E-03	9.69E-04	3.99E-03		1.45E-02	4.74E-03	2.76E-03	2.05E-02	9.39E-03	1.19E-03	1.21E-02	1.60E-02		2.22E-04	3.15E-04
aadA_5				8.07E-03	6.40E-03	7.31E-03	6.36E-03	4.29E-03	3.34E-03	5.34E-03	6.14E-03	1.19E-02	3.10E-02	1.32E-02	2.77E-02	1.55E-02	8.44E-02	1.16E-02	1.12E-02	3.67E-02	3.04E-02	7.87E-02	2.62E-04	9.55E-04
aadA_6				3.81E-03	3.34E-03	4.42E-03	1.97E-03	1.61E-03	1.08E-03	1.37E-03	1.39E-03	3.94E-03	1.17E-02	5.04E-03	6.58E-03	1.74E-03	1.29E-02	1.09E-03	7.17E-04	1.55E-03				1.61E-04
aadA5_1				2.05E-04	4.27E-04	1.58E-04	5.59E-04	1.51E-04	2.16E-04	3.64E-04	3.33E-04	3.55E-04	1.09E-03	9.92E-04	1.52E-03	7.17E-04	1.02E-03	7.61E-04						
aadA5_2				4.17E-04	3.42E-04	4.95E-04	8.77E-04	1.85E-04	3.64E-04	3.93E-04	3.54E-04	5.87E-04	1.54E-03	1.42E-03	1.51E-03	8.38E-04	6.17E-04	8.62E-04						
aadA9_1																7.06E-05								
aadA9_2				1.14E-04												1.21E-04	1.65E-04							
aadD							3.14E-04	5.71E-05	2.21E-03							5.39E-05		1.17E-04						
aadE				1.61E-03	1.12E-03	1.61E-03	2.82E-04	3.08E-05	2.05E-03			6.05E-05	1.39E-04	4.25E-05	4.64E-05	5.20E-04	1.06E-03	3.77E-04						
aph(2')-Id													1.88E-05											
aphA1/7				1.47E-04			5.63E-04	1.30E-04	7.21E-04	3.81E-05		7.29E-05	1.06E-04	2.62E-05	5.44E-05	4.41E-05	2.69E-04	5.15E-05						
aphA3_1							1.58E-04	1.90E-05	6.93E-04		3.58E-05		7.96E-05	9.59E-05	3.85E-05		4.16E-04	8.60E-05						
aphA3_2							2.46E-04	1.26E-04	7.81E-04	5.08E-05	5.46E-05	1.37E-04	1.95E-04	2.09E-05	8.52E-05	1.17E-04	4.61E-04	8.54E-05						
str							4.35E-05		7.11E-04															
strA									7.93E-05			4.25E-05	3.39E-05	2.43E-05	2.62E-05			6.17E-05						
strB				3.00E-03	2.25E-03	2.57E-03	2.25E-03	5.59E-04	5.47E-03	1.24E-03	1.87E-03	2.77E-03	6.60E-03	6.10E-03	6.92E-03	6.28E-03	7.64E-03	2.86E-03	1.50E-03	3.75E-03	6.14E-03			1.23E-04
catA1												4.43E-05	3.98E-05	1.86E-05	2.05E-05									
catB3				6.34E-04	6.30E-04	8.01E-04	4.04E-04	6.85E-05	2.12E-04	1.90E-04	1.97E-04	3.49E-04	1.16E-03	6.22E-04	6.95E-04	3.46E-04	7.73E-04	1.21E-04						
catB8						1.49E-04	9.92E-05			1.34E-04	1.42E-04	2.24E-04	7.20E-04	4.46E-04	3.06E-04	5.98E-05	4.79E-04	1.31E-04		1.77E-03				
cmlA_2				5.78E-04	6.63E-04	8.70E-04	1.15E-03	3.07E-04	3.52E-04	1.26E-03	1.33E-03	4.22E-03	7.16E-03	6.77E-03	6.50E-03	2.45E-03	1.16E-02	1.64E-03		3.67E-03	2.65E-03		9.82E-05	1.59E-04
cmlA_3				9.37E-04	9.20E-04	1.29E-03	1.24E-03	3.28E-04	2.43E-04	1.96E-03	1.48E-03	4.35E-03	7.02E-03	7.78E-03	6.15E-03	1.81E-03	1.02E-02	3.05E-03	7.93E-04	2.84E-03	1.88E-03		4.09E-05	
cmlA_4				1.01E-03	7.99E-04	7.11E-04	9.36E-04	1.81E-04	1.94E-04	1.16E-03	1.48E-03	4.18E-03	6.82E-03	7.10E-03	6.72E-03	2.20E-03	1.51E-02	1.38E-03	9.73E-04	1.50E-03	2.28E-03		6.89E-05	7.17E-05
cmxA							3.97E-04	4.83E-04	5.45E-04	7.51E-05	1.19E-04	3.45E-04	1.24E-04	6.32E-05	7.95E-05	4.56E-05	3.38E-04		2.35E-03	6.22E-03	7.33E-03			1.43E-04
floR_1				1.80E-03	1.23E-03	1.89E-03	1.14E-03	6.50E-04	7.64E-04	4.22E-04	1.02E-03	1.68E-03	2.42E-03	2.35E-03	1.60E-03	8.47E-04	2.76E-03	8.56E-04	3.11E-03	9.04E-03	1.15E-02	4.60E-02	1.51E-04	3.66E-04
mdtL				1.42E-04		2.32E-04	8.99E-05	2.03E-05	1.61E-04	8.91E-05		2.17E-04	2.88E-04	2.39E-04	2.22E-04	4.72E-05	2.38E-04	9.12E-05						

rarD_2				1.08E-04		1.79E-04			1.28E-04	3.38E-05	3.86E-05	6.64E-05	1.89E-04	5.94E-05	7.86E-05									
ampC_1							5.85E-05		7.41E-05	4.58E-05	5.99E-05	1.85E-04	2.03E-04	1.86E-04	1.58E-04	8.31E-05								
ampC_2							5.59E-05		1.03E-04	3.52E-05		1.56E-04	2.36E-04	1.64E-04	2.66E-04	5.60E-05	1.71E-04	4.94E-05						
ampC_5							3.59E-05						8.43E-05	1.15E-04	1.70E-04	7.92E-05	3.67E-04	8.92E-05						
blaACT_1									7.92E-05															
blaACT_2									5.28E-05				4.26E-03											
blaACT_3				9.10E-04	6.71E-04	6.28E-04			6.85E-05	1.48E-03			3.48E-02	1.64E-05		2.52E-03	8.33E-03	3.14E-03						
blaCMY_1									8.99E-04				1.13E-04	7.82E-05	1.30E-04		2.67E-04							
blaCMY_2									8.97E-04				8.84E-04	9.76E-05	1.29E-04									
blaCMY_3				1.04E-03	6.08E-04	1.05E-03	3.78E-05	4.23E-05		2.09E-04	3.60E-04	5.66E-04	7.14E-03	8.41E-05	1.52E-04		6.64E-04	6.70E-05						
blaCTX-M_2														1.28E-05										
blaCTX-M_4									3.54E-05			4.31E-05												
blaCTX-M_7									3.84E-05															
blaDHA				5.30E-03	4.63E-03	5.58E-03							6.77E-03											
blaFOX							5.72E-05			6.92E-05	9.76E-05	1.29E-04	6.38E-05	3.62E-05	2.07E-04	9.21E-05		9.71E-05					5.48E-05	1.29E-04
blaGES					1.98E-04		3.19E-04	9.03E-05	2.91E-04	2.27E-03	1.02E-03	4.80E-03	5.96E-03	7.16E-03	2.26E-03	2.27E-03	3.95E-02	1.37E-02	4.13E-03	2.20E-02	5.02E-02	7.75E-02	9.89E-04	8.04E-04
blaIMP_3										5.88E-05		5.09E-05	1.57E-05		7.04E-05			4.89E-05						
blaKPC_1								6.71E-05						1.41E-05										
blaL1									4.84E-05															
blaMOX/blaCMY				1.87E-04	4.55E-04	3.42E-04	9.79E-05	5.56E-05	3.97E-05	1.63E-04	2.65E-04	5.67E-04	2.67E-04	2.15E-04	1.11E-04	7.83E-05	5.26E-04	1.05E-04						
blaOXA_1				1.93E-03	1.92E-03	2.20E-03	2.27E-03	4.63E-04	4.64E-04	4.73E-03	2.24E-03	4.91E-03	2.01E-02	2.00E-02	2.43E-02	1.56E-02	1.33E-02	1.55E-02					3.17E-04	2.15E-04
blaOXA_2										7.70E-03	3.19E-03	9.66E-03	3.54E-02		1.38E-02	7.98E-03	4.62E-02	2.17E-02	1.39E-03	1.07E-02	1.30E-02			3.67E-04
blaOXA_3				6.62E-04	6.76E-04	7.70E-04	1.15E-04	2.51E-05		4.25E-04	3.24E-04	3.49E-04	1.18E-03	1.70E-03	2.33E-03	4.69E-04	1.39E-03	2.82E-04						
blaOXA_4				4.14E-04	8.50E-04	8.23E-04	2.81E-04	1.71E-04	7.45E-05		3.66E-05	5.78E-05	1.17E-04	7.09E-05	8.31E-05		2.11E-04							

blaPDC														1.45E-05										
blaPER															2.09E-05									
blaPSE				1.16E-04	1.83E-04		8.92E-05	7.48E-05		1.34E-04	8.50E-05	7.98E-05	5.63E-04	6.00E-04	8.77E-04	2.38E-04	4.28E-04	1.06E-04						
blaROB													2.63E-05	1.44E-05	2.06E-05									
blaSFO													3.19E-05	1.73E-05	4.01E-05	4.33E-05								
blaSHV_1									1.52E-04				2.73E-05	3.36E-05	2.85E-05									
blaSHV_2									5.57E-05				3.91E-05	1.41E-05										
blaTEM				1.89E-04			1.80E-04		1.79E-04	7.06E-05	4.39E-05	2.33E-04	4.45E-04	3.35E-04	5.25E-04	2.34E-04	4.57E-04	8.54E-05						
blaTLA										5.79E-05	3.93E-05		2.49E-04	2.77E-04	2.97E-04	8.33E-04	1.57E-03							
blaVEB						2.15E-04				7.85E-04	5.86E-04	1.56E-03		5.70E-03	4.22E-03	4.95E-04	3.72E-03	8.80E-04		1.31E-03			6.15E-05	8.16E-05
ccrA														2.12E-05										
cepA															2.52E-05									
cfxA					5.60E-03		5.03E-05		6.98E-05	1.67E-04	2.14E-04	3.46E-04	6.01E-04	6.69E-04	7.03E-04	1.58E-04	3.58E-04	2.33E-04						
cphA_1				2.59E-04	3.19E-04	3.46E-04		5.29E-05		1.33E-04	7.74E-05	1.80E-04	1.25E-04	1.50E-04	5.22E-05			1.17E-04	8.21E-04					
cphA_2							4.16E-05	7.83E-05				4.96E-05	5.24E-05	7.75E-05	4.05E-05									
pbp									5.47E-04															
intI1_1				2.02E-03	2.90E-03	3.37E-03	2.99E-03	9.01E-04	8.45E-04	5.98E-03	3.35E-03	9.51E-03	2.34E-02	3.07E-02	9.52E-03	3.73E-03	3.15E-02	9.01E-03	6.87E-03	5.89E-02	7.45E-02	9.26E-02	1.98E-03	1.24E-03
intI1_2				1.99E-03	2.17E-03	1.59E-03	4.00E-03	8.97E-04	5.47E-04			7.85E-03	2.12E-02	4.55E-03	1.14E-02	6.57E-03	2.19E-02	6.11E-03		2.53E-02	3.82E-02		5.37E-04	1.79E-03
intI1_3				1.30E-03	1.80E-03	1.50E-03	3.10E-03	1.29E-03	3.97E-04	2.75E-03		8.15E-03	1.54E-02	3.80E-03	1.12E-02		2.00E-02	4.13E-03	1.09E-02		4.09E-02	5.00E-02		1.42E-03
intI1_4				4.61E-04	4.04E-04	3.79E-04				4.18E-04		9.91E-04	3.16E-03	6.26E-04	1.09E-03	1.13E-03	2.72E-03	9.08E-04		6.66E-03	7.64E-03			1.99E-04
intI2_1				2.33E-04																				
intI2_2													1.38E-05											
intI3_1				3.21E-04	3.25E-04	2.27E-04	1.30E-04	2.51E-05		7.18E-05	5.95E-05	8.41E-05	3.04E-04	1.28E-04	2.29E-04	7.81E-05	2.34E-04		6.00E-03	1.29E-02	1.85E-02	4.88E-02	5.65E-04	7.73E-04
intI3_2				1.58E-04	2.25E-04	2.78E-04				9.74E-05	3.58E-05	1.13E-04	2.91E-04	1.25E-04	2.18E-04				3.79E-03	1.46E-02	1.90E-02		2.38E-04	6.01E-04
acrA_1				9.87E-05					1.19E-04			6.77E-05	1.26E-04	1.68E-04	6.45E-05									

acrA_2						1.22E-04			9.84E-05	5.04E-05		7.39E-05	1.33E-04	1.56E-04	4.44E-05		2.47E-04							
acrA_4							3.51E-05		1.82E-04			5.79E-05	3.05E-05											
acrA_5							4.30E-05					5.04E-05	1.90E-05		3.75E-05									
acrB_1						2.35E-04	4.31E-05		1.76E-04	1.02E-04	3.61E-05	1.61E-04	2.34E-04	2.23E-04	1.94E-04									
acrB_2															2.82E-05									
acrF							5.22E-05		1.15E-04	3.39E-05	3.84E-05	7.60E-05	1.66E-04	1.90E-04	2.26E-04	5.64E-05		9.68E-05						
acrR_1									9.48E-05	2.89E-05			2.53E-05	1.88E-05										
acrR_2				1.39E-04			4.75E-05		8.41E-05	6.66E-05		1.82E-04	2.06E-04	1.90E-04	1.18E-04	7.69E-05	2.36E-04	6.08E-05						
acrR_3							1.79E-04			7.70E-05	4.99E-05	1.65E-04	2.20E-04	2.20E-04	9.48E-05			1.11E-04						
cmr							2.90E-05																	
emrB_2													2.18E-05											
marR_3									4.59E-05															
mdtA									2.40E-04				2.25E-05	3.04E-05		7.62E-05	2.98E-04	8.22E-05						
mdtE						1.38E-04			4.55E-05	6.54E-05	3.96E-05	9.93E-05	2.65E-04	2.85E-04	4.22E-04	1.50E-04	3.34E-04	6.41E-05						
mdtF				1.48E-04	3.54E-04	1.48E-04	9.64E-05		2.11E-04	7.49E-05	6.19E-05	2.57E-04	4.03E-04	3.58E-04	3.94E-04	7.46E-05	2.52E-04	6.25E-05		2.68E-03				
mdtG_1						1.70E-04	4.24E-05		1.33E-04	7.75E-05			1.88E-04	3.68E-04	2.81E-04	5.92E-05	2.09E-04							
mdtH_1							5.42E-05		5.97E-05					2.08E-04										
mdtH_2				2.07E-04	2.81E-04	1.62E-04	6.08E-05	3.00E-05	2.19E-04	7.49E-05			1.88E-04	1.39E-04	1.67E-04	4.77E-05								
mexB													1.61E-05		3.03E-05									
mexE							3.12E-04	1.15E-04	3.97E-05	1.76E-04	2.16E-04	4.27E-04	2.29E-04	2.01E-04	1.86E-04	1.39E-04	3.60E-04	3.06E-04		3.24E-03				
mexF				3.15E-03	3.52E-03		7.10E-04			4.23E-04	1.02E-03	1.14E-03	6.90E-04	4.82E-04	1.45E-03	5.02E-04	2.51E-03	7.22E-04	5.01E-02	8.11E-02	1.63E-01	3.42E-01	1.78E-03	3.80E-03
mtrD_3							8.36E-05	5.05E-05				9.62E-05												
oprD												4.10E-05	5.87E-05	2.97E-05	9.81E-05									
oprJ						9.46E-05		2.64E-05		3.92E-05		4.03E-05	2.41E-05	2.07E-05	5.64E-05					1.99E-03	2.31E-03			
qacH_1				2.14E-04	1.35E-04	1.22E-04	5.36E-04	1.63E-04	3.96E-04	2.23E-04	1.89E-04	5.01E-04	8.88E-04	8.30E-04	8.39E-04	4.65E-04	1.10E-03	5.06E-04	9.37E-04	2.17E-03				

qacH_2				1.49E-04	1.83E-04	1.06E-04	1.01E-03	1.83E-04	5.30E-04	1.46E-03	1.32E-03	2.41E-03	4.32E-03	3.75E-03	3.92E-03	2.45E-03	7.97E-03	2.86E-03	3.91E-03	1.66E-02	1.17E-02	3.52E-02	3.10E-04	2.28E-04
tolC_1				2.13E-04	1.50E-04	9.34E-05	3.62E-05		1.77E-04	4.39E-05	4.04E-05	1.94E-04	2.06E-04	2.66E-04	3.66E-04	1.22E-04								
tolC_2									9.49E-04				3.38E-05		3.29E-05									
tolC_3									8.29E-04			4.05E-05	2.36E-05	1.37E-05	4.51E-05									
IncN_rep							9.52E-05		2.87E-04			1.13E-04	2.22E-04	8.16E-05	1.72E-04			6.54E-05						
IncP_1alpha				3.00E-04	2.05E-04	2.42E-04	6.81E-04	2.49E-04	1.14E-04		7.27E-04	9.03E-04		4.03E-04	1.41E-03	8.07E-04			3.68E-03	7.81E-03	1.31E-02		5.31E-05	
IncP_1beta											4.56E-05	1.28E-04	2.39E-05		4.15E-05									
IncP_oriT				1.09E-04			3.04E-05			2.90E-05		4.16E-05	7.08E-05	3.21E-05	6.17E-05		2.01E-04							
IncQ_oriT													7.56E-05	3.18E-05	6.59E-05									
IS1133									5.15E-05															
IS613				7.36E-04	3.81E-04		3.15E-04	5.27E-05	2.79E-04	1.44E-03	1.42E-03	3.46E-03	3.10E-03	2.12E-03	3.97E-03	6.94E-03	1.18E-02	2.94E-03	9.14E-04					
ISAba3				9.02E-04	1.09E-03	8.40E-04	2.99E-04	1.57E-04	8.01E-05		2.90E-04	2.90E-04	2.86E-04	3.53E-04	2.56E-04	2.22E-04	2.65E-04	2.00E-04						
ISEfm1							7.10E-05	5.01E-05	9.23E-04				2.38E-05	2.27E-05	2.08E-05	7.97E-05								
ISPps				2.52E-03	3.97E-03	2.88E-03	1.07E-02	2.16E-03	4.67E-03	6.39E-03	7.67E-03	1.69E-02	1.73E-02	1.92E-02	1.88E-02	5.98E-03	1.81E-02	8.26E-03	4.40E-02	7.68E-02	1.31E-01	1.65E-01	2.23E-03	1.46E-03
ISSm2				1.15E-03	1.44E-03	1.73E-03	3.40E-03	8.74E-04	3.17E-03	2.39E-03	2.18E-03	5.10E-03	4.43E-03	3.50E-03	3.82E-03	3.01E-03	7.48E-03	2.96E-03	7.48E-02	1.83E-01	2.81E-01	5.91E-01	4.98E-03	5.83E-03
orf39-IS26									9.53E-05				2.64E-05											
repA				1.98E-04	1.55E-04					5.68E-05	1.33E-04	3.58E-04	6.08E-05		6.15E-05		2.60E-04		1.07E-03	3.54E-03	3.58E-03			3.05E-04
tnpA_1				6.40E-03	5.97E-03	5.39E-03	4.63E-03	2.40E-03	5.55E-03	6.82E-03	6.60E-03	1.37E-02	2.66E-02	2.17E-02	2.69E-02	1.07E-02	3.88E-02	9.07E-03	5.50E-03	1.21E-02	1.69E-02	3.44E-02	9.69E-05	3.35E-04
tnpA_2				5.87E-03	5.38E-03	6.66E-03	1.05E-02	2.37E-03	6.35E-03	6.80E-03	7.96E-03	1.59E-02	2.68E-02	2.19E-02	3.30E-02	1.74E-02	4.38E-02	1.57E-02	1.25E-01	1.97E-01	3.62E-01	4.62E-01	4.04E-03	6.22E-03
tnpA_3				8.08E-04	9.44E-04	8.71E-04	8.78E-04	5.79E-04	1.30E-03	5.99E-04	8.01E-04	1.23E-03		1.39E-03		1.04E-03	3.46E-03	7.35E-04		1.45E-03				
tnpA_4						2.40E-04	1.03E-04	3.50E-05		8.74E-05	7.30E-05	3.63E-04	4.37E-04	1.65E-04	4.30E-04	2.92E-04	8.86E-04	2.33E-04						
tnpA_5				1.41E-04	2.13E-04	3.96E-04	1.29E-04	4.46E-05	3.36E-05	2.89E-05	3.61E-05	8.42E-05	2.86E-04	1.34E-04	3.07E-04	1.56E-04	2.91E-04	1.13E-04						
tnpA_6				6.01E-04	3.35E-04	3.76E-04	4.50E-04	6.43E-05	8.68E-03	1.45E-04	2.31E-04	4.95E-04	9.68E-04	4.61E-04	6.19E-04	2.51E-03	6.07E-03	1.46E-03						
tnpA_7				2.69E-04	3.33E-04	5.30E-04	4.36E-05		1.42E-03	4.53E-05	3.65E-05	1.04E-04	5.69E-04	2.07E-04	4.57E-04	1.19E-03	3.50E-03	6.52E-04		1.52E-03				
Tp614				5.20E-04	5.32E-04	6.58E-04	2.94E-05	2.11E-05	1.13E-04				1.46E-04	1.73E-04	1.91E-04	3.49E-04	3.24E-04	1.02E-04						

trfa																				1.91E-03			
ereA				1.72E-04	2.29E-04	1.57E-04	4.89E-04	1.41E-04	3.92E-04	1.22E-03	1.14E-03	1.61E-03	2.11E-03	2.02E-03	3.08E-03	1.23E-03	1.37E-03	8.62E-04	2.23E-03	1.74E-03	5.54E-03		7.30E-05	
ereB													4.28E-05	6.20E-05	2.59E-05									
erm(35)										4.55E-05			8.36E-05	6.76E-05	2.18E-05	8.63E-05								
erm(36)								3.32E-05	1.72E-04															
ermA									3.63E-05															
ermB							3.36E-04	2.87E-05	1.05E-03	4.82E-05	1.02E-04	1.00E-04	6.61E-04	1.56E-04	5.81E-04	2.70E-04	3.17E-04	1.60E-04						
ermC							1.50E-04	8.04E-05	1.53E-03															
ermF							2.40E-04	5.09E-05	5.97E-04	3.26E-04	3.13E-04	7.30E-04	2.04E-03	1.92E-03	1.07E-03	1.03E-03	6.09E-03	7.74E-04						
ermT_1																6.03E-05		6.09E-05						
ermT_2							4.02E-05		4.97E-05							8.17E-05	2.32E-04	1.27E-04						
ermX							4.45E-04	8.93E-05	3.54E-04							9.94E-04	9.45E-04	6.69E-04						
ermY									3.34E-05															
lmrA_1									5.01E-04				1.86E-05											
lnuA_1							9.79E-05	3.08E-05	9.03E-04				5.40E-05	7.50E-05	6.45E-05	6.56E-04	2.65E-04	2.81E-04						
lnuB_1				2.10E-04	1.36E-04	2.84E-04	9.16E-05	7.72E-05	6.64E-04	8.37E-05		1.46E-04	1.02E-03	8.13E-04	9.40E-04	2.13E-03	7.94E-03	1.41E-03						
lnuB_2						1.37E-04	1.23E-04	3.50E-05	5.57E-04	6.49E-05		1.33E-04	8.46E-04	5.08E-04	1.01E-03	1.70E-03	3.94E-03	6.56E-04						
matA/mel				1.14E-04	1.59E-04		1.52E-04	6.26E-05	2.58E-04	3.88E-05		1.37E-04	9.83E-04	6.07E-04	1.16E-03	1.04E-03	1.93E-03	6.81E-04						
mefA				9.55E-04	4.66E-04	9.22E-04	1.67E-04	2.49E-05	3.01E-04			6.17E-05	2.87E-04	5.55E-05	1.70E-04	1.20E-04	6.83E-04	1.10E-04						
mphA_1						3.95E-04				4.91E-05	4.71E-05	5.38E-05	1.48E-04	9.79E-05	2.07E-04	1.55E-04		2.50E-04						
mphA_2				2.60E-04	2.29E-04	2.29E-04	5.54E-05		1.36E-04	3.78E-05	3.99E-05	1.09E-04	1.51E-04	1.52E-04		7.67E-05	3.51E-04	9.95E-05						
msrC_1									5.68E-05															
pncA							4.74E-05	1.97E-05				8.38E-05												
vatE_1							7.05E-05	3.05E-05								9.64E-05	4.12E-04							
vatE_2							9.76E-05	4.51E-05	1.05E-04				1.90E-05			1.54E-04	1.16E-03							

qacEdelta1_1				6.74E-03	5.35E-03	8.67E-03	7.23E-03	1.96E-03	2.53E-03	8.78E-03	7.79E-03	2.01E-02	3.79E-02	2.24E-02	3.06E-02	1.94E-02	6.84E-02	1.56E-02	1.09E-02	3.04E-02	3.78E-02	2.90E-02	2.49E-04	7.88E-04
qacEdelta1_2				7.88E-03	6.93E-03	7.74E-03	7.39E-03	1.78E-03	3.44E-03	1.07E-02	5.22E-03	2.30E-02	4.21E-02	4.12E-02	1.98E-02	1.33E-02	1.29E-01	2.97E-02	2.40E-03	5.75E-02	8.12E-02	1.63E-01	1.09E-03	1.03E-03
qacEdelta1_3				7.05E-03	6.69E-03	6.29E-03	5.92E-03	1.14E-03	2.61E-03	8.55E-03	6.96E-03	1.68E-02	3.21E-02	2.62E-02	1.38E-02	7.76E-03	1.48E-01	1.86E-02	9.52E-03	4.33E-02	6.09E-02	7.54E-02	6.70E-04	1.10E-03
bacA_2				1.47E-04		1.57E-04	2.80E-05		1.15E-04			1.24E-04	2.25E-04	2.77E-04	6.23E-05		1.50E-04							
merA				3.68E-04	3.15E-04	5.55E-04	4.85E-04	3.24E-04	1.12E-04	4.48E-04	6.58E-05	2.83E-04	2.58E-03	3.04E-03	4.09E-03	6.10E-04	1.36E-03	7.25E-04		1.97E-03	2.86E-03			
nisB_2									1.37E-04															
sat4							2.10E-04	5.07E-05	4.65E-04			1.28E-04	1.02E-04	4.52E-05	7.67E-05	1.47E-04	3.32E-04							
qnrA														2.63E-05										
qnrB									3.83E-04															
sul1_2				9.70E-03	8.08E-03	9.55E-03	1.26E-02	4.66E-03	6.92E-03		1.03E-02	1.92E-02	4.22E-02	2.15E-02	2.66E-02	1.59E-02	7.74E-02	1.19E-02	7.97E-02	2.01E-01	1.77E-01	4.33E-01	3.28E-03	8.37E-03
sul2_1				4.22E-03	4.35E-03	4.02E-03	5.47E-03	3.04E-03	3.73E-03	1.84E-03	1.98E-03	3.38E-03	9.89E-03	5.30E-03	6.00E-03	1.40E-02	4.96E-02	1.18E-02	1.75E-02	4.87E-02	4.49E-02	1.41E-01	7.27E-04	2.17E-03
sul2_2				4.85E-04	4.09E-04	5.63E-04	7.27E-04	1.49E-04	3.02E-04			5.19E-04	2.81E-03	2.27E-03	4.66E-03	7.95E-03	6.43E-03	7.16E-03	7.14E-03	1.26E-02	2.44E-02		3.31E-04	
sul3										1.04E-04	5.65E-05	1.07E-04	2.00E-04	1.32E-04	9.61E-05	2.20E-04	6.17E-04	1.29E-04						
tet(32)				1.50E-03	9.77E-04	1.32E-03	1.79E-04	3.03E-05		1.05E-04	9.47E-05	1.67E-04	7.86E-04	7.35E-04	4.30E-04	4.29E-04	1.81E-03	1.98E-04						
tet(36)_1							4.93E-05							1.78E-05			3.68E-04	6.26E-05						
tet(36)_2																7.06E-05								
tetA_2				2.15E-03	1.51E-03	2.02E-03	3.50E-03	1.35E-03	1.13E-03	3.02E-03	3.64E-03	6.00E-03	6.26E-03	5.68E-03	6.68E-03	5.88E-03	1.38E-02	3.76E-03	2.19E-02	4.16E-02	4.96E-02	5.72E-02	3.60E-04	4.37E-04
tetA(P)				1.98E-04									3.04E-05	2.13E-05	2.77E-05	1.70E-04								
tetA/B_1							7.75E-05	3.28E-05	6.25E-05		4.30E-05	6.01E-05	5.25E-05	7.01E-05	3.09E-05		3.30E-04	8.81E-05						
tetA/B_2							1.06E-04		4.01E-05			5.47E-05	6.06E-05	3.44E-05	2.24E-05		2.65E-04							
tetC_1							5.40E-05	3.94E-05		6.50E-05	4.30E-05	1.33E-04	3.67E-05	2.41E-05		8.78E-05	2.53E-04	7.81E-05						
tetC_2							9.62E-05	4.41E-05	4.31E-05	3.05E-05		4.28E-05	3.74E-05	3.58E-05	1.97E-05		2.50E-04							
tetC_3				4.43E-04	7.74E-04	6.05E-04	5.08E-04	9.05E-05	1.03E-04	9.05E-04	5.27E-04	1.26E-03	3.25E-03	1.41E-03	3.63E-03	8.89E-04	1.06E-03	7.90E-04			2.44E-03		4.16E-05	
tetD_1									5.72E-05															
tetD_3									3.73E-05															

tetE				2.41E-04	2.81E-04	2.95E-04	2.07E-04	1.21E-04	3.92E-05	1.43E-04	1.46E-04	1.19E-04	4.89E-04	5.06E-04	7.38E-04	5.53E-04	2.05E-04	2.25E-04						
tetG_1				6.42E-04	5.52E-04	5.17E-04	1.24E-03	3.40E-04	8.51E-04	1.49E-03	9.26E-04	2.33E-03	2.51E-03	1.43E-03	1.84E-03		9.84E-03	1.78E-03	4.46E-03	3.52E-02	2.86E-02	3.16E-02	2.65E-04	4.79E-04
tetG_2				1.95E-03	2.37E-03	2.17E-03	2.78E-03	5.53E-04	1.09E-03	8.30E-04	1.69E-03	2.57E-03	3.97E-03	1.84E-03	2.15E-03	2.08E-03	4.55E-03	1.39E-03	2.52E-03	2.40E-02	1.25E-02		5.16E-04	8.31E-04
tetH					2.55E-04		5.86E-05		1.40E-03				3.81E-04	1.56E-04	2.44E-04	7.55E-05	2.46E-04							
tetJ									9.78E-05															
tetL_2				1.71E-04	2.82E-04	1.03E-04	4.60E-04	1.85E-04	5.81E-03	3.33E-05	3.97E-05	1.99E-04	2.15E-04	1.69E-04	2.69E-04	2.50E-03	2.79E-03	1.29E-03						
tetM_1				2.51E-03	1.60E-03	1.52E-03	4.46E-04	6.55E-05	1.96E-03	1.42E-04	1.52E-04	3.89E-04	2.42E-03	1.15E-03	1.42E-03	2.60E-03	1.34E-02	1.01E-03						
tetM_2				6.30E-04	5.44E-04	5.78E-04	1.79E-04	1.67E-04	9.48E-04	1.24E-04	8.04E-05	1.51E-04	1.68E-03	7.80E-04	1.98E-03	3.56E-03	3.68E-03	2.02E-03						
tetM_3				1.25E-04	1.77E-04	1.43E-04				4.67E-05	7.28E-05		8.72E-04	4.54E-04	9.44E-04	1.74E-03	1.42E-03	1.08E-03						
tetO_1				1.43E-03	1.50E-03	1.95E-03	1.32E-04	2.43E-05	8.79E-05	1.46E-04	1.51E-04	3.72E-04	1.63E-03	1.45E-03	1.92E-03	2.76E-03	5.11E-03	1.22E-03						
tetO_2				1.18E-03	1.15E-03	1.61E-03	8.88E-05	3.51E-05	5.10E-05	1.72E-04	1.09E-04	3.47E-04	1.50E-03	8.01E-04	1.12E-03	1.44E-03	3.43E-03	6.42E-04						
tetPB_3					1.48E-04	1.25E-04	5.71E-05						1.92E-05											
tetQ				2.58E-03	1.78E-03	1.76E-03	2.98E-04	6.49E-05	3.78E-04	2.87E-04	3.63E-04	5.49E-04	2.17E-03	6.24E-04	2.33E-03	9.77E-04	3.64E-03	7.35E-04	6.49E-04					
tetR_2				1.33E-03	1.01E-03	1.15E-03	1.11E-03	1.09E-03	6.31E-04	2.52E-03	1.33E-03	3.96E-03	4.96E-03	4.68E-03	2.14E-03	3.04E-03	1.12E-02	4.55E-03	5.10E-03	5.52E-02	5.99E-02	4.39E-02	1.87E-04	5.00E-04
tetR_3				1.28E-04			9.83E-05	3.57E-05	4.83E-05		3.84E-05		4.27E-05	3.54E-05		6.73E-05	1.53E-04	6.02E-05						
tetS					1.36E-04	1.11E-04			4.22E-04	4.87E-05		4.68E-05	2.02E-04	1.58E-04	3.82E-05	1.30E-04	6.31E-04	1.71E-04						
tetT							3.12E-05									2.43E-04	3.96E-04	1.04E-04						
tetW				3.95E-03	3.32E-03	2.63E-03	8.62E-04	2.33E-04	4.59E-04	1.35E-04	1.98E-04	6.31E-04	1.63E-03	1.32E-03	8.10E-04	2.09E-03	2.70E-03	6.86E-04						
tetX				6.99E-04	8.27E-04	5.03E-04	2.15E-03	4.72E-04	2.42E-03	3.00E-04	1.97E-04	3.37E-04	7.28E-04	5.69E-04	7.61E-04	1.72E-03	1.38E-03	1.27E-03						
dfrA1_1				1.49E-03	8.02E-04	1.06E-03	2.42E-04	8.42E-05	5.56E-05	1.22E-03	8.49E-04	1.09E-03	1.80E-03	2.10E-03	2.82E-03	1.29E-03	1.27E-03	1.27E-03						
dfrA1_2							7.84E-05	3.58E-05	5.58E-05	3.20E-04	1.26E-04	5.79E-04	7.13E-04	1.14E-03	3.19E-04	1.01E-04	9.45E-04	2.69E-04						
dfrA12									3.63E-05				3.17E-05	2.89E-05	5.11E-05									
vanC_1									1.20E-04															
vanC_3							2.95E-05			4.57E-05		5.00E-05	1.62E-05		2.57E-05									
vanC_4									7.94E-05															

vanC2/vanC3									1.44E-04															
vanRC									5.47E-05															
vanSC_1									5.38E-05															
vanSC_2									7.48E-05															
vanTC_1									2.05E-04															
vanTC_3									6.84E-05															
vanYD_1													3.19E-05		4.53E-05									

Table S3. Pairwise comparisons of gamma distribution GLMs of relative abundances of each ARG or MGE between treatment groups

Comparison (X_1-X_2)	Gene	Change	Delta.Estimate (X_1-X_2)(A	Std.error	z-value	p.adjusted	Fold.Change (X_1-X_2)(B	Std.Error of the Fold.Change	log2 Fold.Change	log2 Std.Error of FC
City - Rural	*aac(6)-Ib_1*	increase from rural to city	1.38	0.33	4.18	0.00	3.99	1.39	2.00	0.48
Estuary - Rural	*aac(6)-Ib_1*	decrease from rural to estuary	-6.35	0.33	-19.15	0.00	0.00	1.39	-9.16	0.48
Estuary - City	*aac(6)-Ib_1*	decrease from city to estuary	-7.73	0.33	-23.33	0.00	0.00	1.39	-11.16	0.48
City - Rural	*aac(6)-Ib_2*	increase from rural to city	1.71	0.26	6.63	0.00	5.55	1.29	2.47	0.37
Estuary - Rural	*aac(6)-Ib_2*	decrease from rural to estuary	-6.24	0.26	-24.15	0.00	0.00	1.29	-9.00	0.37
Estuary - City	*aac(6)-Ib_2*	decrease from city to estuary	-7.95	0.26	-30.78	0.00	0.00	1.29	-11.47	0.37
City - Rural	*aac(6)-Ib_3*	increase from rural to city	1.66	0.25	6.70	0.00	5.24	1.28	2.39	0.36

**Estuary - Rural**	*aac(6)-Ib_3*	decrease from rural to estuary	-6.64	0.25	-26.86	0.00	0.00	1.28	-9.57	0.36
**Estuary - City**	*aac(6)-Ib_3*	decrease from city to estuary	-8.29	0.25	-33.56	0.00	0.00	1.28	-11.96	0.36
**City - Rural**	*aac(6)-II*	increase from rural to city	5.92	0.26	22.77	0.00	370.99	1.30	8.54	0.37
**Estuary - City**	*aac(6)-II*	decrease from city to estuary	-5.92	0.26	-22.77	0.00	0.00	1.30	-8.54	0.37
**City - Rural**	*aacA_aphD*	decrease from rural to city	-2.87	0.77	-3.73	0.00	0.06	2.16	-4.15	1.11
**Estuary - Rural**	*aacA_aphD*	decrease from rural to estuary	-2.87	0.77	-3.73	0.00	0.06	2.16	-4.15	1.11
**Estuary - Rural**	*aacC2*	decrease from rural to estuary	-3.39	0.87	-3.89	0.00	0.03	2.39	-4.88	1.26
**Estuary - City**	*aacC2*	decrease from city to estuary	-3.73	0.87	-4.28	0.00	0.02	2.39	-5.38	1.26
**City - Rural**	*aacC4*	decrease from rural to city	-2.82	0.77	-3.68	0.00	0.06	2.16	-4.07	1.11
**Estuary - Rural**	*aacC4*	decrease from rural to estuary	-2.82	0.77	-3.68	0.00	0.06	2.16	-4.07	1.11
**City - Rural**	*aadA_2*	increase from rural to city	1.37	0.50	2.74	0.02	3.93	1.65	1.98	0.72
**Estuary - City**	*aadA_3*	decrease from city to estuary	-1.82	0.62	-2.93	0.01	0.16	1.86	-2.63	0.90
**Estuary - Rural**	*aadA_5*	increase from rural to estuary	1.49	0.45	3.33	0.00	4.42	1.56	2.15	0.64

**Estuary - Rural**	*aadA_6*	decrease from rural to estuary	-4.61	0.84	-5.47	0.00	0.01	2.32	-6.65	1.22
**Estuary - City**	*aadA_6*	decrease from city to estuary	-5.17	0.84	-6.13	0.00	0.01	2.32	-7.46	1.22
**City - Rural**	*aadA5_1*	increase from rural to city	0.91	0.32	2.86	0.01	2.49	1.38	1.31	0.46
**Estuary - Rural**	*aadA5_1*	decrease from rural to estuary	-5.69	0.32	-17.84	0.00	0.00	1.38	-8.21	0.46
**Estuary - City**	*aadA5_1*	decrease from city to estuary	-6.60	0.32	-20.70	0.00	0.00	1.38	-9.52	0.46
**City - Rural**	*aadA5_2*	increase from rural to city	0.63	0.26	2.42	0.04	1.88	1.30	0.91	0.38
**Estuary - Rural**	*aadA5_2*	decrease from rural to estuary	-6.11	0.26	-23.42	0.00	0.00	1.30	-8.81	0.38
**Estuary - City**	*aadA5_2*	decrease from city to estuary	-6.74	0.26	-25.84	0.00	0.00	1.30	-9.72	0.38
**City - Rural**	*aadD*	decrease from rural to city	-6.12	0.68	-9.03	0.00	0.00	1.97	-8.83	0.98
**Estuary - Rural**	*aadD*	decrease from rural to estuary	-6.12	0.68	-9.03	0.00	0.00	1.97	-8.83	0.98
**City - Rural**	*aadE*	decrease from rural to city	-3.27	0.50	-6.47	0.00	0.04	1.66	-4.71	0.73
**Estuary - Rural**	*aadE*	decrease from rural to estuary	-7.03	0.50	-13.93	0.00	0.00	1.66	-10.14	0.73
**Estuary - City**	*aadE*	decrease from city to estuary	-3.76	0.50	-7.46	0.00	0.02	1.66	-5.43	0.73

**Estuary - Rural**	*acrA_1*	decrease from rural to estuary	-3.08	0.86	-3.56	0.00	0.05	2.37	-4.44	1.25
**Estuary - City**	*acrA_1*	decrease from city to estuary	-4.04	0.86	-4.68	0.00	0.02	2.37	-5.83	1.25
**Estuary - Rural**	*acrA_2*	decrease from rural to estuary	-2.89	0.83	-3.50	0.00	0.06	2.28	-4.17	1.19
**Estuary - City**	*acrA_2*	decrease from city to estuary	-4.43	0.83	-5.36	0.00	0.01	2.28	-6.39	1.19
**Estuary - Rural**	*acrA_4*	decrease from rural to estuary	-3.49	1.05	-3.31	0.00	0.03	2.86	-5.03	1.52
**City - Rural**	*acrA_5*	increase from rural to city	1.94	0.70	2.78	0.02	6.99	2.01	2.80	1.01
**Estuary - City**	*acrA_5*	decrease from city to estuary	-1.94	0.70	-2.78	0.02	0.14	2.01	-2.80	1.01
**Estuary - Rural**	*acrB_1*	decrease from rural to estuary	-4.19	0.56	-7.43	0.00	0.02	1.76	-6.05	0.81
**Estuary - City**	*acrB_1*	decrease from city to estuary	-5.06	0.56	-8.97	0.00	0.01	1.76	-7.30	0.81
**Estuary - Rural**	*acrF*	decrease from rural to estuary	-3.04	0.82	-3.70	0.00	0.05	2.28	-4.38	1.19
**Estuary - City**	*acrF*	decrease from city to estuary	-4.76	0.82	-5.78	0.00	0.01	2.28	-6.86	1.19
**City - Rural**	*acrR_1*	decrease from rural to city	-2.86	0.77	-3.71	0.00	0.06	2.16	-4.12	1.11

**Estuary - Rural**	*acrR_1*	decrease from rural to estuary	-2.86	0.77	-3.71	0.00	0.06	2.16	-4.12	1.11
**City - Rural**	*acrR_2*	increase from rural to city	2.09	0.81	2.59	0.03	8.09	2.24	3.02	1.17
**Estuary - Rural**	*acrR_2*	decrease from rural to estuary	-2.74	0.81	-3.39	0.00	0.06	2.24	-3.96	1.17
**Estuary - City**	*acrR_2*	decrease from city to estuary	-4.83	0.81	-5.98	0.00	0.01	2.24	-6.97	1.17
**Estuary - Rural**	*acrR_3*	decrease from rural to estuary	-3.40	0.82	-4.17	0.00	0.03	2.26	-4.91	1.18
**Estuary - City**	*acrR_3*	decrease from city to estuary	-4.93	0.82	-6.04	0.00	0.01	2.26	-7.11	1.18
**City - Rural**	*ampC_1*	increase from rural to city	1.54	0.57	2.69	0.02	4.66	1.77	2.22	0.82
**Estuary - Rural**	*ampC_1*	decrease from rural to estuary	-3.21	0.57	-5.62	0.00	0.04	1.77	-4.63	0.82
**Estuary - City**	*ampC_1*	decrease from city to estuary	-4.75	0.57	-8.31	0.00	0.01	1.77	-6.85	0.82
**City - Rural**	*ampC_2*	increase from rural to city	1.47	0.57	2.57	0.03	4.34	1.77	2.12	0.82
**Estuary - Rural**	*ampC_2*	decrease from rural to estuary	-3.50	0.57	-6.13	0.00	0.03	1.77	-5.05	0.82
**Estuary - City**	*ampC_2*	decrease from city to estuary	-4.96	0.57	-8.70	0.00	0.01	1.77	-7.16	0.82
**City - Rural**	*ampC_5*	increase from rural to city	3.94	0.36	10.79	0.00	51.31	1.44	5.68	0.53

**Estuary - City**	*ampC_5*	decrease from city to estuary	-3.94	0.36	-10.79	0.00	0.02	1.44	-5.68	0.53
**City - Rural**	*aphA1_7*	decrease from rural to city	-1.98	0.60	-3.31	0.00	0.14	1.82	-2.86	0.86
**Estuary - Rural**	*aphA1_7*	decrease from rural to estuary	-5.55	0.60	-9.29	0.00	0.00	1.82	-8.01	0.86
**Estuary - City**	*aphA1_7*	decrease from city to estuary	-3.57	0.60	-5.98	0.00	0.03	1.82	-5.16	0.86
**Estuary - Rural**	*aphA3_1*	decrease from rural to estuary	-5.01	0.75	-6.64	0.00	0.01	2.12	-7.22	1.09
**Estuary - City**	*aphA3_1*	decrease from city to estuary	-3.52	0.75	-4.68	0.00	0.03	2.12	-5.08	1.09
**Estuary - Rural**	*aphA3_2*	decrease from rural to estuary	-5.29	0.61	-8.75	0.00	0.01	1.83	-7.64	0.87
**Estuary - City**	*aphA3_2*	decrease from city to estuary	-4.43	0.61	-7.32	0.00	0.01	1.83	-6.39	0.87
**Estuary - Rural**	*bacA_2*	decrease from rural to estuary	-3.05	0.84	-3.64	0.00	0.05	2.31	-4.39	1.21
**Estuary - City**	*bacA_2*	decrease from city to estuary	-4.73	0.84	-5.65	0.00	0.01	2.31	-6.82	1.21
**City - Rural**	*blaACT_2*	increase from rural to city	6.61	0.82	8.11	0.00	746.15	2.26	9.54	1.18
**Estuary - City**	*blaACT_2*	decrease from city to estuary	-6.61	0.82	-8.11	0.00	0.00	2.26	-9.54	1.18
**City - Rural**	*blaACT_3*	increase from rural to city	2.76	0.84	3.28	0.00	15.74	2.32	3.98	1.21

**Estuary - Rural**	*blaACT_3*	decrease from rural to estuary	-6.01	0.84	-7.15	0.00	0.00	2.32	-8.67	1.21
**Estuary - City**	*blaACT_3*	decrease from city to estuary	-8.77	0.84	-10.43	0.00	0.00	2.32	-12.65	1.21
**Estuary - Rural**	*blaCMY_1*	decrease from rural to estuary	-5.06	0.89	-5.70	0.00	0.01	2.43	-7.30	1.28
**Estuary - City**	*blaCMY_1*	decrease from city to estuary	-4.06	0.89	-4.57	0.00	0.02	2.43	-5.85	1.28
**Estuary - Rural**	*blaCMY_2*	decrease from rural to estuary	-5.06	1.04	-4.88	0.00	0.01	2.82	-7.30	1.49
**Estuary - City**	*blaCMY_2*	decrease from city to estuary	-5.24	1.04	-5.06	0.00	0.01	2.82	-7.56	1.49
**Estuary - Rural**	*blaCMY_3*	decrease from rural to estuary	-6.21	0.76	-8.18	0.00	0.00	2.14	-8.97	1.10
**Estuary - City**	*blaCMY_3*	decrease from city to estuary	-7.30	0.76	-9.61	0.00	0.00	2.14	-10.53	1.10
**Estuary - Rural**	*blaDHA*	decrease from rural to estuary	-7.91	0.89	-8.84	0.00	0.00	2.45	-11.40	1.29
**Estuary - City**	*blaDHA*	decrease from city to estuary	-7.08	0.89	-7.91	0.00	0.00	2.45	-10.21	1.29
**City - Rural**	*blaFOX*	increase from rural to city	2.26	0.76	3.00	0.01	9.62	2.13	3.27	1.09
**Estuary - Rural**	*blaFOX*	decrease from rural to estuary	-2.38	0.76	-3.15	0.01	0.09	2.13	-3.44	1.09

**Estuary - City**	*blaFOX*	decrease from city to estuary	-4.65	0.76	-6.15	0.00	0.01	2.13	-6.70	1.09
**City - Rural**	*blaGES*	increase from rural to city	3.48	0.62	5.59	0.00	32.52	1.86	5.02	0.90
**Estuary - Rural**	*blaGES*	increase from rural to estuary	5.34	0.62	8.58	0.00	209.43	1.86	7.71	0.90
**Estuary - City**	*blaGES*	increase from city to estuary	1.86	0.62	2.99	0.01	6.44	1.86	2.69	0.90
**City - Rural**	*blaIMP_3*	increase from rural to city	2.40	0.74	3.23	0.00	10.97	2.10	3.46	1.07
**Estuary - City**	*blaIMP_3*	decrease from city to estuary	-2.40	0.74	-3.23	0.00	0.09	2.10	-3.46	1.07
**City - Rural**	*blaKPC_1*	decrease from rural to city	-2.70	0.76	-3.54	0.00	0.07	2.14	-3.89	1.10
**Estuary - Rural**	*blaKPC_1*	decrease from rural to estuary	-2.70	0.76	-3.54	0.00	0.07	2.14	-3.89	1.10
**Estuary - Rural**	*blaMOX_blaCMY*	decrease from rural to estuary	-5.31	0.35	-14.98	0.00	0.00	1.43	-7.66	0.51
**Estuary - City**	*blaMOX_blaCMY*	decrease from city to estuary	-5.51	0.35	-15.56	0.00	0.00	1.43	-7.95	0.51
**City - Rural**	*blaOXA_1*	increase from rural to city	1.96	0.61	3.24	0.00	7.10	1.83	2.83	0.87
**Estuary - Rural**	*blaOXA_1*	decrease from rural to estuary	-2.81	0.61	-4.64	0.00	0.06	1.83	-4.05	0.87
**Estuary - City**	*blaOXA_1*	decrease from city to estuary	-4.77	0.61	-7.87	0.00	0.01	1.83	-6.88	0.87

**City - Rural**	*blaOXA_2*	increase from rural to city	9.46	0.58	16.28	0.00	12878.70	1.79	13.65	0.84
**Estuary - Rural**	*blaOXA_2*	increase from rural to estuary	8.40	0.58	14.45	0.00	4446.77	1.79	12.12	0.84
**Estuary - Rural**	*blaOXA_3*	decrease from rural to estuary	-5.95	0.38	-15.52	0.00	0.00	1.47	-8.59	0.55
**Estuary - City**	*blaOXA_3*	decrease from city to estuary	-6.80	0.38	-17.72	0.00	0.00	1.47	-9.81	0.55
**City - Rural**	*blaOXA_4*	decrease from rural to city	-2.44	0.47	-5.18	0.00	0.09	1.60	-3.52	0.68
**Estuary - Rural**	*blaOXA_4*	decrease from rural to estuary	-6.08	0.47	-12.92	0.00	0.00	1.60	-8.77	0.68
**Estuary - City**	*blaOXA_4*	decrease from city to estuary	-3.64	0.47	-7.74	0.00	0.03	1.60	-5.25	0.68
**City - Rural**	*blaPSE*	increase from rural to city	2.30	0.56	4.11	0.00	9.97	1.75	3.32	0.81
**Estuary - Rural**	*blaPSE*	decrease from rural to estuary	-3.46	0.56	-6.19	0.00	0.03	1.75	-5.00	0.81
**Estuary - City**	*blaPSE*	decrease from city to estuary	-5.76	0.56	-10.30	0.00	0.00	1.75	-8.31	0.81
**City - Rural**	*blaSFO*	increase from rural to city	2.59	0.48	5.36	0.00	13.28	1.62	3.73	0.70
**Estuary - City**	*blaSFO*	decrease from city to estuary	-2.59	0.48	-5.36	0.00	0.08	1.62	-3.73	0.70
**Estuary - Rural**	*blaSHV_1*	decrease from rural to estuary	-3.09	0.92	-3.38	0.00	0.05	2.50	-4.46	1.32

**Estuary - City**	*blaSHV_1*	decrease from city to estuary	-2.65	0.92	-2.89	0.01	0.07	2.50	-3.82	1.32
**City - Rural**	*blaSHV_2*	increase from rural to city	2.04	0.71	2.87	0.01	7.67	2.03	2.94	1.02
**Estuary - City**	*blaSHV_2*	decrease from city to estuary	-2.04	0.71	-2.87	0.01	0.13	2.03	-2.94	1.02
**Estuary - Rural**	*blaTEM*	decrease from rural to estuary	-4.26	0.58	-7.29	0.00	0.01	1.79	-6.15	0.84
**Estuary - City**	*blaTEM*	decrease from city to estuary	-5.41	0.58	-9.26	0.00	0.00	1.79	-7.80	0.84
**City - Rural**	*blaTLA*	increase from rural to city	4.83	0.37	13.02	0.00	125.36	1.45	6.97	0.54
**Estuary - City**	*blaTLA*	decrease from city to estuary	-4.83	0.37	-13.02	0.00	0.01	1.45	-6.97	0.54
**City - Rural**	*blaVEB*	increase from rural to city	3.81	0.86	4.41	0.00	45.00	2.37	5.49	1.25
**Estuary - Rural**	*blaVEB*	decrease from rural to estuary	-3.65	0.86	-4.22	0.00	0.03	2.37	-5.26	1.25
**Estuary - City**	*blaVEB*	decrease from city to estuary	-7.46	0.86	-8.63	0.00	0.00	2.37	-10.76	1.25
**City - Rural**	*catA1*	increase from rural to city	2.05	0.71	2.88	0.01	7.79	2.04	2.96	1.03
**Estuary - City**	*catA1*	decrease from city to estuary	-2.05	0.71	-2.88	0.01	0.13	2.04	-2.96	1.03
**Estuary - Rural**	*catB3*	decrease from rural to estuary	-6.18	0.29	-21.57	0.00	0.00	1.33	-8.92	0.41
**Estuary - City**	*catB3*	decrease from city to estuary	-6.40	0.29	-22.32	0.00	0.00	1.33	-9.23	0.41

**City - Rural**	*catB8*	increase from rural to city	2.98	0.81	3.70	0.00	19.66	2.24	4.30	1.16
**Estuary - Rural**	*catB8*	decrease from rural to estuary	-2.90	0.81	-3.60	0.00	0.06	2.24	-4.18	1.16
**Estuary - City**	*catB8*	decrease from city to estuary	-5.88	0.81	-7.30	0.00	0.00	2.24	-8.48	1.16
**Estuary - Rural**	*cfxA*	decrease from rural to estuary	-6.91	0.82	-8.47	0.00	0.00	2.26	-9.97	1.18
**Estuary - City**	*cfxA*	decrease from city to estuary	-6.01	0.82	-7.37	0.00	0.00	2.26	-8.67	1.18
**City - Rural**	*cmlA_2*	increase from rural to city	1.87	0.79	2.37	0.05	6.48	2.20	2.70	1.14
**Estuary - City**	*cmlA_2*	decrease from city to estuary	-1.89	0.79	-2.39	0.04	0.15	2.20	-2.72	1.14
**City - Rural**	*cmlA_3*	increase from rural to city	1.69	0.24	6.91	0.00	5.40	1.28	2.43	0.35
**Estuary - Rural**	*cmlA_3*	decrease from rural to estuary	-6.76	0.24	-27.70	0.00	0.00	1.28	-9.76	0.35
**Estuary - City**	*cmlA_3*	decrease from city to estuary	-8.45	0.24	-34.60	0.00	0.00	1.28	-12.19	0.35
**Estuary - Rural**	*cmlA_4*	decrease from rural to estuary	-3.57	0.82	-4.36	0.00	0.03	2.27	-5.14	1.18
**Estuary - City**	*cmlA_4*	decrease from city to estuary	-5.43	0.82	-6.64	0.00	0.00	2.27	-7.84	1.18
**Estuary - Rural**	*cmxA*	increase from rural to estuary	2.72	0.59	4.57	0.00	15.21	1.81	3.93	0.86

**Estuary - City**	*cmxA*	increase from city to estuary	3.05	0.59	5.13	0.00	21.18	1.81	4.40	0.86
**Estuary - Rural**	*cphA_1*	decrease from rural to estuary	-5.09	0.40	-12.76	0.00	0.01	1.49	-7.34	0.58
**Estuary - City**	*cphA_1*	decrease from city to estuary	-4.78	0.40	-11.98	0.00	0.01	1.49	-6.89	0.58
**Estuary - Rural**	*cphA_2*	decrease from rural to estuary	-2.85	0.92	-3.09	0.01	0.06	2.51	-4.11	1.33
**Estuary - City**	*cphA_2*	decrease from city to estuary	-3.15	0.92	-3.42	0.00	0.04	2.51	-4.55	1.33
**City - Rural**	*dfrA1_1*	increase from rural to city	1.07	0.33	3.19	0.00	2.91	1.40	1.54	0.48
**Estuary - Rural**	*dfrA1_1*	decrease from rural to estuary	-6.34	0.33	-18.92	0.00	0.00	1.40	-9.14	0.48
**Estuary - City**	*dfrA1_1*	decrease from city to estuary	-7.40	0.33	-22.11	0.00	0.00	1.40	-10.68	0.48
**City - Rural**	*dfrA1_2*	increase from rural to city	3.56	0.78	4.59	0.00	35.33	2.18	5.14	1.12
**Estuary - Rural**	*dfrA1_2*	decrease from rural to estuary	-2.68	0.78	-3.45	0.00	0.07	2.18	-3.86	1.12
**Estuary - City**	*dfrA1_2*	decrease from city to estuary	-6.24	0.78	-8.03	0.00	0.00	2.18	-9.01	1.12
**City - Rural**	*dfrA12*	increase from rural to city	3.00	0.35	8.58	0.00	20.16	1.42	4.33	0.50
**Estuary - City**	*dfrA12*	decrease from city to estuary	-3.00	0.35	-8.58	0.00	0.05	1.42	-4.33	0.50

**City - Rural**	*ereA*	increase from rural to city	2.29	0.73	3.16	0.01	9.92	2.07	3.31	1.05
**Estuary - Rural**	*ereA*	increase from rural to estuary	2.03	0.73	2.80	0.02	7.61	2.07	2.93	1.05
**City - Rural**	*ereB*	increase from rural to city	3.34	0.36	9.27	0.00	28.17	1.43	4.82	0.52
**Estuary - City**	*ereB*	decrease from city to estuary	-3.34	0.36	-9.27	0.00	0.04	1.43	-4.82	0.52
**City - Rural**	*erm(35)*	increase from rural to city	3.38	0.50	6.72	0.00	29.40	1.65	4.88	0.73
**Estuary - City**	*erm(35)*	decrease from city to estuary	-3.38	0.50	-6.72	0.00	0.03	1.65	-4.88	0.73
**City - Rural**	*erm(36)*	decrease from rural to city	-3.43	0.79	-4.34	0.00	0.03	2.20	-4.95	1.14
**Estuary - Rural**	*erm(36)*	decrease from rural to estuary	-3.43	0.79	-4.34	0.00	0.03	2.20	-4.95	1.14
**Estuary - Rural**	*ermB*	decrease from rural to estuary	-5.50	0.73	-7.58	0.00	0.00	2.06	-7.93	1.05
**Estuary - City**	*ermB*	decrease from city to estuary	-5.41	0.73	-7.45	0.00	0.00	2.06	-7.80	1.05
**City - Rural**	*ermC*	decrease from rural to city	-5.74	0.68	-8.41	0.00	0.00	1.98	-8.28	0.99
**Estuary - Rural**	*ermC*	decrease from rural to estuary	-5.74	0.68	-8.41	0.00	0.00	1.98	-8.28	0.99
**City - Rural**	*ermF*	increase from rural to city	1.93	0.57	3.35	0.00	6.86	1.78	2.78	0.83

**Estuary - Rural**	*ermF*	decrease from rural to estuary	-5.06	0.57	-8.81	0.00	0.01	1.78	-7.30	0.83
**Estuary - City**	*ermF*	decrease from city to estuary	-6.98	0.57	-12.16	0.00	0.00	1.78	-10.08	0.83
**City - Rural**	*ermX*	decrease from rural to city	-5.12	0.43	-12.03	0.00	0.01	1.53	-7.39	0.61
**Estuary - Rural**	*ermX*	decrease from rural to estuary	-5.12	0.43	-12.03	0.00	0.01	1.53	-7.39	0.61
**Estuary - Rural**	*floR_1*	increase from rural to estuary	1.19	0.46	2.57	0.03	3.28	1.59	1.71	0.67
**Estuary - Rural**	*gapA*	decrease from rural to estuary	-4.29	0.58	-7.45	0.00	0.01	1.78	-6.19	0.83
**Estuary - City**	*gapA*	decrease from city to estuary	-4.85	0.58	-8.42	0.00	0.01	1.78	-7.00	0.83
**Estuary - Rural**	*IncN_rep*	decrease from rural to estuary	-4.21	0.67	-6.29	0.00	0.01	1.95	-6.08	0.97
**Estuary - City**	*IncN_rep*	decrease from city to estuary	-4.57	0.67	-6.82	0.00	0.01	1.95	-6.59	0.97
**Estuary - Rural**	*IncP_1alpha*	increase from rural to estuary	2.82	0.54	5.27	0.00	16.81	1.71	4.07	0.77
**Estuary - City**	*IncP_1alpha*	increase from city to estuary	1.67	0.54	3.11	0.01	5.30	1.71	2.41	0.77
**City - Rural**	*IncP_1beta*	increase from rural to city	3.40	0.60	5.67	0.00	29.85	1.82	4.90	0.86

**Estuary - City**	*IncP_1beta*	decrease from city to estuary	-3.40	0.60	-5.67	0.00	0.03	1.82	-4.90	0.86
**City - Rural**	*IncP_oriT*	increase from rural to city	3.31	0.38	8.60	0.00	27.36	1.47	4.77	0.56
**Estuary - City**	*IncP_oriT*	decrease from city to estuary	-3.31	0.38	-8.60	0.00	0.04	1.47	-4.77	0.56
**City - Rural**	*IncQ_oriT*	increase from rural to city	3.43	0.38	9.05	0.00	30.92	1.46	4.95	0.55
**Estuary - City**	*IncQ_oriT*	decrease from city to estuary	-3.43	0.38	-9.05	0.00	0.03	1.46	-4.95	0.55
**City - Rural**	*intI1_1*	increase from rural to city	1.67	0.43	3.88	0.00	5.31	1.54	2.41	0.62
**Estuary - Rural**	*intI1_1*	increase from rural to estuary	2.89	0.43	6.72	0.00	18.06	1.54	4.17	0.62
**Estuary - City**	*intI1_1*	increase from city to estuary	1.22	0.43	2.84	0.01	3.40	1.54	1.77	0.62
**City - Rural**	*intI1_2*	increase from rural to city	1.47	0.54	2.70	0.02	4.33	1.72	2.11	0.78
**Estuary - Rural**	*intI1_2*	increase from rural to estuary	1.95	0.54	3.60	0.00	7.03	1.72	2.81	0.78
**City - Rural**	*intI1_3*	increase from rural to city	1.50	0.49	3.05	0.01	4.48	1.64	2.16	0.71
**Estuary - Rural**	*intI1_3*	increase from rural to estuary	2.15	0.49	4.37	0.00	8.61	1.64	3.11	0.71
**City - Rural**	*intI1_4*	increase from rural to city	1.67	0.66	2.52	0.03	5.34	1.94	2.42	0.96

**Estuary - Rural**	*intI1_4*	increase from rural to estuary	2.47	0.66	3.72	0.00	11.84	1.94	3.57	0.96
**City - Rural**	*intI2_1*	decrease from rural to city	-3.73	0.80	-4.68	0.00	0.02	2.22	-5.38	1.15
**Estuary - Rural**	*intI2_1*	decrease from rural to estuary	-3.73	0.80	-4.68	0.00	0.02	2.22	-5.38	1.15
**Estuary - Rural**	*intI3_1*	increase from rural to estuary	3.52	0.59	5.98	0.00	33.85	1.80	5.08	0.85
**Estuary - City**	*intI3_1*	increase from city to estuary	3.78	0.59	6.42	0.00	43.81	1.80	5.45	0.85
**Estuary - Rural**	*intI3_2*	increase from rural to estuary	3.98	0.70	5.72	0.00	53.38	2.00	5.74	1.00
**Estuary - City**	*intI3_2*	increase from city to estuary	3.80	0.70	5.46	0.00	44.72	2.00	5.48	1.00
**City - Rural**	*IS613*	increase from rural to city	2.19	0.29	7.60	0.00	8.95	1.33	3.16	0.42
**Estuary - Rural**	*IS613*	decrease from rural to estuary	-5.65	0.29	-19.57	0.00	0.00	1.33	-8.15	0.42
**Estuary - City**	*IS613*	decrease from city to estuary	-7.84	0.29	-27.17	0.00	0.00	1.33	-11.31	0.42
**City - Rural**	*ISAba3*	decrease from rural to city	-0.88	0.27	-3.31	0.00	0.41	1.31	-1.28	0.39
**Estuary - Rural**	*ISAba3*	decrease from rural to estuary	-6.41	0.27	-23.98	0.00	0.00	1.31	-9.24	0.39

**Estuary - City**	*ISAba3*	decrease from city to estuary	-5.52	0.27	-20.67	0.00	0.00	1.31	-7.96	0.39
**City - Rural**	*ISEfm1*	decrease from rural to city	-5.24	0.68	-7.70	0.00	0.01	1.97	-7.56	0.98
**Estuary - Rural**	*ISEfm1*	decrease from rural to estuary	-5.24	0.68	-7.70	0.00	0.01	1.97	-7.56	0.98
**City - Rural**	*ISPps*	increase from rural to city	1.11	0.43	2.62	0.02	3.04	1.53	1.61	0.61
**Estuary - Rural**	*ISPps*	increase from rural to estuary	2.71	0.43	6.37	0.00	14.97	1.53	3.90	0.61
**Estuary - City**	*ISPps*	increase from city to estuary	1.59	0.43	3.75	0.00	4.92	1.53	2.30	0.61
**Estuary - Rural**	*ISSm2*	increase from rural to estuary	4.36	0.39	11.08	0.00	78.25	1.48	6.29	0.57
**Estuary - City**	*ISSm2*	increase from city to estuary	3.87	0.39	9.84	0.00	48.11	1.48	5.59	0.57
**City - Rural**	*lmrA_1*	decrease from rural to city	-4.48	0.81	-5.55	0.00	0.01	2.24	-6.47	1.16
**Estuary - Rural**	*lmrA_1*	decrease from rural to estuary	-4.48	0.81	-5.55	0.00	0.01	2.24	-6.47	1.16
**City - Rural**	*lnuA_1*	decrease from rural to city	-2.18	0.87	-2.49	0.03	0.11	2.39	-3.14	1.26
**Estuary - Rural**	*lnuA_1*	decrease from rural to estuary	-5.17	0.87	-5.92	0.00	0.01	2.39	-7.46	1.26
**Estuary - City**	*lnuA_1*	decrease from city to estuary	-2.99	0.87	-3.43	0.00	0.05	2.39	-4.32	1.26

**Estuary - Rural**	*lnuB_1*	decrease from rural to estuary	-5.46	0.47	-11.63	0.00	0.00	1.60	-7.88	0.68
**Estuary - City**	*lnuB_1*	decrease from city to estuary	-6.21	0.47	-13.24	0.00	0.00	1.60	-8.96	0.68
**Estuary - Rural**	*lnuB_2*	decrease from rural to estuary	-4.75	0.74	-6.40	0.00	0.01	2.10	-6.85	1.07
**Estuary - City**	*lnuB_2*	decrease from city to estuary	-5.94	0.74	-8.01	0.00	0.00	2.10	-8.57	1.07
**City - Rural**	*matA_mel*	increase from rural to city	1.56	0.55	2.83	0.01	4.78	1.74	2.26	0.80
**Estuary - Rural**	*matA_mel*	decrease from rural to estuary	-4.44	0.55	-8.03	0.00	0.01	1.74	-6.41	0.80
**Estuary - City**	*matA_mel*	decrease from city to estuary	-6.01	0.55	-10.85	0.00	0.00	1.74	-8.67	0.80
**Estuary - Rural**	*mdh*	decrease from rural to estuary	-4.68	0.55	-8.49	0.00	0.01	1.74	-6.75	0.80
**Estuary - City**	*mdh*	decrease from city to estuary	-4.81	0.55	-8.73	0.00	0.01	1.74	-6.94	0.80
**Estuary - Rural**	*mdtA*	decrease from rural to estuary	-3.75	1.05	-3.58	0.00	0.02	2.86	-5.42	1.52
**City - Rural**	*mdtE*	increase from rural to city	5.15	0.23	22.25	0.00	171.97	1.26	7.43	0.33
**Estuary - City**	*mdtE*	decrease from city to estuary	-5.15	0.23	-22.25	0.00	0.01	1.26	-7.43	0.33

Estuary - Rural	*mdtF*	decrease from rural to estuary	-4.75	0.49	-9.62	0.00	0.01	1.64	-6.86	0.71
Estuary - City	*mdtF*	decrease from city to estuary	-5.49	0.49	-11.11	0.00	0.00	1.64	-7.92	0.71
Estuary - Rural	*mdtG_1*	decrease from rural to estuary	-3.18	0.84	-3.78	0.00	0.04	2.32	-4.59	1.21
Estuary - City	*mdtG_1*	decrease from city to estuary	-4.90	0.84	-5.83	0.00	0.01	2.32	-7.07	1.21
Estuary - Rural	*mdtH_1*	decrease from rural to estuary	-2.81	1.10	-2.54	0.03	0.06	3.02	-4.05	1.59
Estuary - City	*mdtH_1*	decrease from city to estuary	-3.61	1.10	-3.27	0.00	0.03	3.02	-5.21	1.59
Estuary - Rural	*mdtH_2*	decrease from rural to estuary	-4.48	0.58	-7.67	0.00	0.01	1.79	-6.46	0.84
Estuary - City	*mdtH_2*	decrease from city to estuary	-4.70	0.58	-8.06	0.00	0.01	1.79	-6.78	0.84
Estuary - Rural	*mdtL*	decrease from rural to estuary	-4.44	0.45	-9.87	0.00	0.01	1.57	-6.41	0.65
Estuary - City	*mdtL*	decrease from city to estuary	-5.25	0.45	-11.65	0.00	0.01	1.57	-7.57	0.65
City - Rural	*mefA*	decrease from rural to city	-1.72	0.55	-3.13	0.01	0.18	1.73	-2.48	0.79
Estuary - Rural	*mefA*	decrease from rural to estuary	-6.16	0.55	-11.22	0.00	0.00	1.73	-8.88	0.79

**Estuary - City**	*mefA*	decrease from city to estuary	-4.44	0.55	-8.09	0.00	0.01	1.73	-6.41	0.79
**City - Rural**	*merA*	increase from rural to city	1.33	0.30	4.42	0.00	3.76	1.35	1.91	0.43
**Estuary - Rural**	*merA*	decrease from rural to estuary	-5.95	0.30	-19.83	0.00	0.00	1.35	-8.58	0.43
**Estuary - City**	*merA*	decrease from city to estuary	-7.27	0.30	-24.25	0.00	0.00	1.35	-10.49	0.43
**Estuary - Rural**	*mexF*	increase from rural to estuary	4.33	0.60	7.27	0.00	75.57	1.81	6.24	0.86
**Estuary - City**	*mexF*	increase from city to estuary	4.76	0.60	7.99	0.00	116.36	1.81	6.86	0.86
**Estuary - Rural**	*mphA_1*	decrease from rural to estuary	-4.25	0.89	-4.79	0.00	0.01	2.43	-6.13	1.28
**Estuary - City**	*mphA_1*	decrease from city to estuary	-4.20	0.89	-4.74	0.00	0.01	2.43	-6.07	1.28
**Estuary - Rural**	*mphA_2*	decrease from rural to estuary	-4.75	0.53	-8.95	0.00	0.01	1.70	-6.85	0.77
**Estuary - City**	*mphA_2*	decrease from city to estuary	-4.14	0.53	-7.80	0.00	0.02	1.70	-5.97	0.77
**Estuary - Rural**	*mtrD_3*	decrease from rural to estuary	-3.27	0.94	-3.49	0.00	0.04	2.55	-4.72	1.35
**Estuary - City**	*mtrD_3*	decrease from city to estuary	-3.32	0.94	-3.54	0.00	0.04	2.55	-4.79	1.35
**City - Rural**	*nisB_2*	decrease from rural to city	-3.21	0.78	-4.10	0.00	0.04	2.19	-4.63	1.13

**Estuary - Rural**	*nisB_2*	decrease from rural to estuary	-3.21	0.78	-4.10	0.00	0.04	2.19	-4.63	1.13
**City - Rural**	*oprD*	increase from rural to city	3.21	0.50	6.37	0.00	24.90	1.66	4.64	0.73
**Estuary - City**	*oprD*	decrease from city to estuary	-3.21	0.50	-6.37	0.00	0.04	1.66	-4.64	0.73
**City - Rural**	*oprJ*	increase from rural to city	2.19	0.73	3.02	0.01	8.95	2.07	3.16	1.05
**Estuary - City**	*oprJ*	decrease from city to estuary	-2.19	0.73	-3.02	0.01	0.11	2.07	-3.16	1.05
**City - Rural**	*pbp*	decrease from rural to city	-4.57	0.81	-5.65	0.00	0.01	2.24	-6.59	1.17
**Estuary - Rural**	*pbp*	decrease from rural to estuary	-4.57	0.81	-5.65	0.00	0.01	2.24	-6.59	1.17
**City - Rural**	*qacEdelta1_1*	increase from rural to city	1.29	0.48	2.69	0.02	3.65	1.62	1.87	0.69
**City - Rural**	*qacEdelta1_2*	increase from rural to city	1.33	0.47	2.85	0.01	3.79	1.59	1.92	0.67
**Estuary - Rural**	*qacEdelta1_2*	increase from rural to estuary	1.94	0.47	4.16	0.00	6.96	1.59	2.80	0.67
**City - Rural**	*qacEdelta1_3*	increase from rural to city	1.21	0.44	2.72	0.02	3.35	1.56	1.74	0.64
**Estuary - Rural**	*qacEdelta1_3*	increase from rural to estuary	1.78	0.44	4.00	0.00	5.92	1.56	2.57	0.64
**City - Rural**	*qacH_1*	increase from rural to city	1.15	0.44	2.58	0.03	3.14	1.56	1.65	0.64

**Estuary - Rural**	*qacH_1*	decrease from rural to estuary	-5.28	0.44	-11.90	0.00	0.01	1.56	-7.62	0.64
**Estuary - City**	*qacH_1*	decrease from city to estuary	-6.43	0.44	-14.48	0.00	0.00	1.56	-9.28	0.64
**City - Rural**	*qacH_2*	increase from rural to city	2.08	0.58	3.62	0.00	8.03	1.78	3.01	0.83
**Estuary - Rural**	*qacH_2*	increase from rural to estuary	2.67	0.58	4.65	0.00	14.49	1.78	3.86	0.83
**City - Rural**	*qnrA*	increase from rural to city	1.82	0.68	2.66	0.02	6.17	1.98	2.63	0.99
**Estuary - City**	*qnrA*	decrease from city to estuary	-1.82	0.68	-2.66	0.02	0.16	1.98	-2.63	0.99
**City - Rural**	*qnrB*	decrease from rural to city	-4.22	0.80	-5.24	0.00	0.01	2.24	-6.08	1.16
**Estuary - Rural**	*qnrB*	decrease from rural to estuary	-4.22	0.80	-5.24	0.00	0.01	2.24	-6.08	1.16
**Estuary - Rural**	*rarD_2*	decrease from rural to estuary	-3.14	0.98	-3.21	0.00	0.04	2.66	-4.53	1.41
**Estuary - City**	*rarD_2*	decrease from city to estuary	-3.81	0.98	-3.89	0.00	0.02	2.66	-5.49	1.41
**City - Rural**	*repA*	increase from rural to city	4.66	0.87	5.38	0.00	105.61	2.38	6.72	1.25
**Estuary - Rural**	*repA*	increase from rural to estuary	6.51	0.87	7.53	0.00	673.46	2.38	9.40	1.25

**Estuary - Rural**	*rpoB*	increase from rural to estuary	2.81	0.39	7.29	0.00	16.61	1.47	4.05	0.56
**Estuary - City**	*rpoB*	increase from city to estuary	2.47	0.39	6.42	0.00	11.88	1.47	3.57	0.56
**Estuary - Rural**	*sat4*	decrease from rural to estuary	-4.86	0.59	-8.24	0.00	0.01	1.80	-7.01	0.85
**Estuary - City**	*sat4*	decrease from city to estuary	-4.09	0.59	-6.93	0.00	0.02	1.80	-5.90	0.85
**City - Rural**	*str*	decrease from rural to city	-4.83	0.81	-5.96	0.00	0.01	2.25	-6.97	1.17
**Estuary - Rural**	*str*	decrease from rural to estuary	-4.83	0.81	-5.96	0.00	0.01	2.25	-6.97	1.17
**City - Rural**	*strA*	decrease from rural to city	-2.69	0.76	-3.53	0.00	0.07	2.14	-3.88	1.10
**Estuary - Rural**	*strA*	decrease from rural to estuary	-2.69	0.76	-3.53	0.00	0.07	2.14	-3.88	1.10
**Estuary - Rural**	*sul1_2*	increase from rural to estuary	2.87	0.40	7.22	0.00	17.69	1.49	4.15	0.57
**Estuary - City**	*sul1_2*	increase from city to estuary	2.02	0.40	5.07	0.00	7.53	1.49	2.91	0.57
**Estuary - Rural**	*sul2_1*	increase from rural to estuary	2.28	0.41	5.55	0.00	9.76	1.51	3.29	0.59
**Estuary - City**	*sul2_1*	increase from city to estuary	2.16	0.41	5.25	0.00	8.63	1.51	3.11	0.59

**Estuary - Rural**	*sul2_2*	increase from rural to estuary	2.60	0.55	4.72	0.00	13.49	1.73	3.75	0.79
**Estuary - City**	*sul2_2*	increase from city to estuary	1.50	0.55	2.72	0.02	4.47	1.73	2.16	0.79
**City - Rural**	*sul3*	increase from rural to city	4.82	0.18	26.04	0.00	123.56	1.20	6.95	0.27
**Estuary - City**	*sul3*	decrease from city to estuary	-4.82	0.18	-26.04	0.00	0.01	1.20	-6.95	0.27
**Estuary - Rural**	*tet(32)*	decrease from rural to estuary	-6.53	0.45	-14.68	0.00	0.00	1.56	-9.43	0.64
**Estuary - City**	*tet(32)*	decrease from city to estuary	-5.86	0.45	-13.17	0.00	0.00	1.56	-8.46	0.64
**City - Rural**	*tetA_2*	increase from rural to city	0.95	0.35	2.72	0.02	2.58	1.42	1.37	0.50
**Estuary - Rural**	*tetA_2*	increase from rural to estuary	2.69	0.35	7.71	0.00	14.71	1.42	3.88	0.50
**Estuary - City**	*tetA_2*	increase from city to estuary	1.74	0.35	4.99	0.00	5.70	1.42	2.51	0.50
**Estuary - Rural**	*tetA_B_1*	decrease from rural to estuary	-2.67	0.91	-2.93	0.01	0.07	2.48	-3.85	1.31
**Estuary - City**	*tetA_B_1*	decrease from city to estuary	-3.10	0.91	-3.40	0.00	0.05	2.48	-4.47	1.31
**Estuary - Rural**	*tetA_B_2*	decrease from rural to estuary	-2.77	0.92	-3.00	0.01	0.06	2.51	-3.99	1.33
**Estuary - City**	*tetA_B_2*	decrease from city to estuary	-2.85	0.92	-3.09	0.01	0.06	2.51	-4.11	1.33

**City - Rural**	*tetA(P)*	increase from rural to city	2.15	0.72	2.98	0.01	8.58	2.06	3.10	1.04
**Estuary - City**	*tetA(P)*	decrease from city to estuary	-2.15	0.72	-2.98	0.01	0.12	2.06	-3.10	1.04
**Estuary - Rural**	*tetC_1*	decrease from rural to estuary	-2.53	0.87	-2.92	0.01	0.08	2.38	-3.64	1.25
**Estuary - City**	*tetC_1*	decrease from city to estuary	-3.79	0.87	-4.37	0.00	0.02	2.38	-5.46	1.25
**Estuary - Rural**	*tetC_2*	decrease from rural to estuary	-3.42	0.69	-4.93	0.00	0.03	2.00	-4.93	1.00
**Estuary - City**	*tetC_2*	decrease from city to estuary	-2.60	0.69	-3.75	0.00	0.07	2.00	-3.75	1.00
**City - Rural**	*tetC_3*	increase from rural to city	1.38	0.31	4.48	0.00	3.97	1.36	1.99	0.44
**Estuary - Rural**	*tetC_3*	decrease from rural to estuary	-6.11	0.31	-19.84	0.00	0.00	1.36	-8.81	0.44
**Estuary - City**	*tetC_3*	decrease from city to estuary	-7.49	0.31	-24.32	0.00	0.00	1.36	-10.80	0.44
**Estuary - Rural**	*tetE*	decrease from rural to estuary	-5.11	0.32	-16.06	0.00	0.01	1.37	-7.37	0.46
**Estuary - City**	*tetE*	decrease from city to estuary	-5.83	0.32	-18.30	0.00	0.00	1.37	-8.40	0.46
**Estuary - Rural**	*tetG_1*	increase from rural to estuary	2.79	0.50	5.58	0.00	16.32	1.65	4.03	0.72
**Estuary - City**	*tetG_1*	increase from city to estuary	1.87	0.50	3.75	0.00	6.51	1.65	2.70	0.72

**Estuary - Rural**	*tetG_2*	increase from rural to estuary	1.37	0.51	2.69	0.02	3.94	1.67	1.98	0.74
**Estuary - City**	*tetG_2*	increase from city to estuary	1.20	0.51	2.35	0.05	3.32	1.67	1.73	0.74
**Estuary - Rural**	*tetH*	decrease from rural to estuary	-5.55	0.87	-6.35	0.00	0.00	2.39	-8.00	1.26
**Estuary - City**	*tetH*	decrease from city to estuary	-4.85	0.87	-5.55	0.00	0.01	2.39	-6.99	1.26
**City - Rural**	*tetJ*	decrease from rural to city	-2.89	0.77	-3.74	0.00	0.06	2.16	-4.16	1.11
**Estuary - Rural**	*tetJ*	decrease from rural to estuary	-2.89	0.77	-3.74	0.00	0.06	2.16	-4.16	1.11
**City - Rural**	*tetL_2*	decrease from rural to city	-2.27	0.77	-2.96	0.01	0.10	2.16	-3.28	1.11
**Estuary - Rural**	*tetL_2*	decrease from rural to estuary	-7.03	0.77	-9.16	0.00	0.00	2.16	-10.15	1.11
**Estuary - City**	*tetL_2*	decrease from city to estuary	-4.76	0.77	-6.20	0.00	0.01	2.16	-6.87	1.11
**Estuary - Rural**	*tetM_1*	decrease from rural to estuary	-7.18	0.38	-18.91	0.00	0.00	1.46	-10.36	0.55
**Estuary - City**	*tetM_1*	decrease from city to estuary	-6.84	0.38	-18.02	0.00	0.00	1.46	-9.87	0.55
**Estuary - Rural**	*tetM_2*	decrease from rural to estuary	-6.27	0.39	-16.01	0.00	0.00	1.48	-9.05	0.57

Estuary - City	*tetM_2*	decrease from city to estuary	-6.66	0.39	-17.00	0.00	0.00	1.48	-9.61	0.57
City - Rural	*tetM_3*	increase from rural to city	5.91	0.39	15.26	0.00	368.29	1.47	8.52	0.56
Estuary - City	*tetM_3*	decrease from city to estuary	-5.91	0.39	-15.26	0.00	0.00	1.47	-8.52	0.56
Estuary - Rural	*tetO_1*	decrease from rural to estuary	-6.79	0.44	-15.57	0.00	0.00	1.55	-9.80	0.63
Estuary - City	*tetO_1*	decrease from city to estuary	-6.84	0.44	-15.69	0.00	0.00	1.55	-9.87	0.63
Estuary - Rural	*tetO_2*	decrease from rural to estuary	-6.66	0.45	-14.82	0.00	0.00	1.57	-9.61	0.65
Estuary - City	*tetO_2*	decrease from city to estuary	-6.51	0.45	-14.47	0.00	0.00	1.57	-9.39	0.65
City - Rural	*tetPB_3*	decrease from rural to city	-2.51	0.75	-3.35	0.00	0.08	2.12	-3.63	1.08
Estuary - Rural	*tetPB_3*	decrease from rural to estuary	-2.51	0.75	-3.35	0.00	0.08	2.12	-3.63	1.08
Estuary - Rural	*tetQ*	decrease from rural to estuary	-7.09	0.41	-17.46	0.00	0.00	1.50	-10.23	0.59
Estuary - City	*tetQ*	decrease from city to estuary	-6.96	0.41	-17.13	0.00	0.00	1.50	-10.04	0.59
Estuary - Rural	*tetR_2*	increase from rural to estuary	2.72	0.48	5.66	0.00	15.12	1.62	3.92	0.69
Estuary - City	*tetR_2*	increase from city to estuary	1.65	0.48	3.43	0.00	5.19	1.62	2.38	0.69

**Estuary - Rural**	*tetR_3*	decrease from rural to estuary	-2.89	1.05	-2.74	0.02	0.06	2.87	-4.17	1.52
**Estuary - Rural**	*tetS*	decrease from rural to estuary	-4.31	0.90	-4.78	0.00	0.01	2.46	-6.22	1.30
**Estuary - City**	*tetS*	decrease from city to estuary	-4.34	0.90	-4.81	0.00	0.01	2.46	-6.26	1.30
**City - Rural**	*tetW*	decrease from rural to city	-0.92	0.37	-2.47	0.04	0.40	1.45	-1.32	0.54
**Estuary - Rural**	*tetW*	decrease from rural to estuary	-7.60	0.37	-20.46	0.00	0.00	1.45	-10.96	0.54
**Estuary - City**	*tetW*	decrease from city to estuary	-6.68	0.37	-17.99	0.00	0.00	1.45	-9.64	0.54
**City - Rural**	*tetX*	decrease from rural to city	-1.09	0.29	-3.81	0.00	0.34	1.33	-1.57	0.41
**Estuary - Rural**	*tetX*	decrease from rural to estuary	-7.13	0.29	-24.96	0.00	0.00	1.33	-10.29	0.41
**Estuary - City**	*tetX*	decrease from city to estuary	-6.04	0.29	-21.15	0.00	0.00	1.33	-8.72	0.41
**Estuary - Rural**	*tolC_1*	decrease from rural to estuary	-3.46	0.81	-4.25	0.00	0.03	2.26	-4.99	1.17
**Estuary - City**	*tolC_1*	decrease from city to estuary	-5.05	0.81	-6.20	0.00	0.01	2.26	-7.28	1.17
**City - Rural**	*tolC_2*	decrease from rural to city	-3.21	1.07	-3.00	0.01	0.04	2.91	-4.63	1.54

**Estuary - Rural**	*tolC_2*	decrease from rural to estuary	-5.12	1.07	-4.79	0.00	0.01	2.91	-7.38	1.54
**City - Rural**	*tolC_3*	decrease from rural to city	-4.98	0.81	-6.14	0.00	0.01	2.25	-7.19	1.17
**Estuary - Rural**	*tolC_3*	decrease from rural to estuary	-4.98	0.81	-6.14	0.00	0.01	2.25	-7.19	1.17
**City - Rural**	*Tp614*	decrease from rural to city	-1.61	0.50	-3.25	0.00	0.20	1.64	-2.33	0.72
**Estuary - Rural**	*Tp614*	decrease from rural to estuary	-5.91	0.50	-11.91	0.00	0.00	1.64	-8.52	0.72
**Estuary - City**	*Tp614*	decrease from city to estuary	-4.29	0.50	-8.65	0.00	0.01	1.64	-6.19	0.72
**Estuary - Rural**	*uidA*	decrease from rural to estuary	-4.55	0.62	-7.31	0.00	0.01	1.86	-6.57	0.90
**Estuary - City**	*uidA*	decrease from city to estuary	-4.32	0.62	-6.93	0.00	0.01	1.86	-6.23	0.90
**City - Rural**	*vanC_1*	decrease from rural to city	-3.08	0.78	-3.96	0.00	0.05	2.18	-4.45	1.12
**Estuary - Rural**	*vanC_1*	decrease from rural to estuary	-3.08	0.78	-3.96	0.00	0.05	2.18	-4.45	1.12
**City - Rural**	*vanC_4*	decrease from rural to city	-2.69	0.76	-3.53	0.00	0.07	2.14	-3.88	1.10
**Estuary - Rural**	*vanC_4*	decrease from rural to estuary	-2.69	0.76	-3.53	0.00	0.07	2.14	-3.88	1.10

**City - Rural**	*vanC2_vanC3*	decrease from rural to city	-3.35	0.79	-4.25	0.00	0.04	2.20	-4.83	1.14
**Estuary - Rural**	*vanC2_vanC3*	decrease from rural to estuary	-3.35	0.79	-4.25	0.00	0.04	2.20	-4.83	1.14
**City - Rural**	*vanSC_2*	decrease from rural to city	-2.63	0.76	-3.47	0.00	0.07	2.13	-3.80	1.09
**Estuary - Rural**	*vanSC_2*	decrease from rural to estuary	-2.63	0.76	-3.47	0.00	0.07	2.13	-3.80	1.09
**City - Rural**	*vanTC_1*	decrease from rural to city	-3.37	0.79	-4.27	0.00	0.03	2.20	-4.86	1.14
**Estuary - Rural**	*vanTC_1*	decrease from rural to estuary	-3.37	0.79	-4.27	0.00	0.03	2.20	-4.86	1.14
**City - Rural**	*vanTC_3*	decrease from rural to city	-2.96	0.77	-3.83	0.00	0.05	2.17	-4.28	1.12
**Estuary - Rural**	*vanTC_3*	decrease from rural to estuary	-2.96	0.77	-3.83	0.00	0.05	2.17	-4.28	1.12
**City - Rural**	*vanYD_1*	increase from rural to city	2.59	0.48	5.38	0.00	13.30	1.62	3.73	0.69
**Estuary - City**	*vanYD_1*	decrease from city to estuary	-2.59	0.48	-5.38	0.00	0.08	1.62	-3.73	0.69
**City - Rural**	*vatE_1*	decrease from rural to city	-2.58	0.75	-3.42	0.00	0.08	2.13	-3.72	1.09
**Estuary - Rural**	*vatE_1*	decrease from rural to estuary	-2.58	0.75	-3.42	0.00	0.08	2.13	-3.72	1.09
**City - Rural**	*vatE_2*	decrease from rural to city	-3.82	0.38	-10.00	0.00	0.02	1.46	-5.51	0.55

**Estuary - Rural**	*vatE_2*	decrease from rural to estuary	-3.82	0.38	-10.00	0.00	0.02	1.46	-5.51	0.55
**Estuary - Rural**	*tnpA-05/IS26*	decrease from rural to estuary	-4.64	0.62	-7.50	0.00	0.01	1.86	-6.70	0.89
**Estuary - City**	*tnpA-05/IS26*	decrease from city to estuary	-4.84	0.62	-7.82	0.00	0.01	1.86	-6.99	0.89
**City - Rural**	*tnpA-04/IS6100*	increase from rural to city	2.60	0.80	3.27	0.00	13.52	2.22	3.76	1.15
**Estuary - Rural**	*tnpA-04/IS6100*	decrease from rural to estuary	-2.93	0.80	-3.68	0.00	0.05	2.22	-4.23	1.15
**Estuary - City**	*tnpA-04/IS6100*	decrease from city to estuary	-5.54	0.80	-6.95	0.00	0.00	2.22	-7.99	1.15
**City - Rural**	*tnpA-02/IS4*	increase from rural to city	0.98	0.39	2.51	0.03	2.67	1.48	1.42	0.56
**Estuary - Rural**	*tnpA-02/IS4*	increase from rural to estuary	3.40	0.39	8.70	0.00	30.00	1.48	4.91	0.56
**Estuary - City**	*tnpA-02/IS4*	increase from city to estuary	2.42	0.39	6.19	0.00	11.24	1.48	3.49	0.56
**City - Rural**	*tnpA-01/IS21*	increase from rural to city	1.19	0.45	2.62	0.02	3.28	1.57	1.71	0.65
**Estuary - Rural**	*tnpA-07/ISEcp1B*	decrease from rural to estuary	-5.96	0.65	-9.20	0.00	0.00	1.91	-8.60	0.93
**Estuary - City**	*tnpA-07/ISEcp1B*	decrease from city to estuary	-5.31	0.65	-8.20	0.00	0.00	1.91	-7.67	0.93

**Estuary - Rural**	*tnpA-06/IS1216*	decrease from rural to estuary	-7.54	0.67	-11.31	0.00	0.00	1.95	-10.88	0.96
**Estuary - City**	*tnpA-06/IS1216*	decrease from city to estuary	-6.17	0.67	-9.25	0.00	0.00	1.95	-8.90	0.96
**City - Rural**	*tnpA-03/IS6*	increase from rural to city	0.47	0.18	2.56	0.03	1.61	1.20	0.68	0.27
**Estuary - Rural**	*tnpA-03/IS6*	decrease from rural to estuary	-6.87	0.18	-37.12	0.00	0.00	1.20	-9.90	0.27
**Estuary - City**	*tnpA-03/IS6*	decrease from city to estuary	-7.34	0.18	-39.69	0.00	0.00	1.20	-10.59	0.27

a) Delta Estimates were calculated using glht function in the multcomp package in R. The glht function calculates the estimates by subtracting the estimate of X2 (second sampling area in the column "Comparison") from the estimate of X1 (first sampling area in the column "Comparison"). Estimates for each treatment group were obtained with Gamma glm models, which were used as the input for the glht function. See the R-script in https://github.com/sjmuurine/CodeRiverIn for the procedure. b) Fold Changes were calculated by taking the exponential function from the Delta Estimate (due to the logarithmic link function that was used in with Gamma glm models). This was done in R using the command exp(Delta.Estimate). See the provided R-script in (https://github.com/sjmuurine/CodeRiverIn for the procedure.

Table S4. Summary of comparison samples presented in Figure 6

Sample type	Cut-off Ct	Number of primers targeting ARGs and MGEs	Reference
Spring Water	27	382	This study
Code River Rural			
Code River City			
Code River Estuary			
FIN Agriculture Ditch Water	27	382	23
FIN Agriculture Soil			
FIN Agriculture Manure			
Antarctic Soil Far From Station	29	382	53
Antarctic Soil Gondwana Station			
Antarctic Soil Jang Bogo Station			

FIN Aquaculture Sea Outside<br>FIN Aquaculture Fish Farm 1<br>FIN Aquaculture Fish Farm 2	27	382	24
CHN Pig Farms Soil<br>CHN Pig Farms Compost<br>CHN Pig Farms Manure	27	313	22
